# The Murine PSE/TATA-Dependent Transcriptome: Evidence of Functional Homologies with Its Human Counterpart

**DOI:** 10.3390/ijms131114813

**Published:** 2012-11-13

**Authors:** Maria Jessica Bruzzone, Paola Gavazzo, Sara Massone, Carolina Balbi, Federico Villa, Anastasia Conti, Giorgio Dieci, Ranieri Cancedda, Aldo Pagano

**Affiliations:** 1 Department of Experimental Medicine (DIMES), University of Genoa, 16132 Genoa, Italy; E-Mails: je.bruzzone@hotmail.com (M.J.B.); sara.massone@gmail.com (S.M.); carolina_balbi@hotmail.it (C.B.); fed_villa@libero.it (F.V.); ranieri.cancedda@unige.it (R.C.); 2 Institute of Biophysics, National Council of Research (CNR), Via De Marini 6, 16149 Genoa, Italy; E-Mail: gavazzo@ge.ibf.cnr.it; 3 Department of Biosciences, University of Parma, Parco Area delle Scienze 23/A, 43124 Parma, Italy; E-Mails: anastasia.conti@hotmail.it (A.C.); giorgio.dieci@unipr.it (G.D.); 4 IRCCS-AOU San Martino-IST, Largo Rosanna Benzi 10, 16132 Genoa, Italy

**Keywords:** RNA polymerase III, alternative splicing, non-coding RNA, potassium channel interacting protein (KCNIP4)

## Abstract

A series of recent studies demonstrated an unexpectedly high frequency of intronic RNA polymerase (pol) III transcription units spread throughout the human genome. The investigation of a subset of these transcripts revealed their tissue/cell-specific transcription together with the involvement in relevant physiopathological pathways. Despite this evidence, these transcripts did not seem to have murine orthologs, based on their nucleotide sequence, resulting in a limitation of the experimental approaches aimed to study their function. In this work, we have extended our investigation to the murine genome identifying 121 pairs of mouse/human transcripts displaying syntenic subchromosomal localization. The analysis *in silico* of this set of putative noncoding (nc)RNAs suggest their association with alternative splicing as suggested by recent experimental evidence. The investigation of one of these pairs taken as experimental model in mouse hippocampal neurons provided evidence of a human/mouse functional homology that does not depend on underlying sequence conservation. In this light, the collection of transcriptional units here reported can be considered as a novel source for the identification and the study of novel regulatory elements involved in relevant biological processes.

## 1. Introduction

RNA polymerase (pol) III drives transcription from three different promoter types referred to as type 1, type 2 and type 3. Type 1 (only found in 5S rRNA genes) and type 2 promoters (typical of tRNA genes) are endowed with transcription control elements located within the transcribed portion of the genes. On the contrary, in type 3 promoters (such as U6 and 7SK) the regulatory elements are positioned upstream to the transcription start site (TSS). Indeed, the type 3-associated Proximal Sequence Element (PSE) maps between −66 and −46, whereas the TATA box maps around −30 in the case of the prototypical human U6 snRNA gene [1]. This peculiar promoter structure, its recently proposed cell type-specific transcription and the novel evidence for an unexpectedly high frequency of this motif throughout the human genome [1,2] make pol III type 3 promoters somehow similar to their pol II-readable counterparts [3,4].

Our first screening *in silico* of the human genome, aimed to identify genomic regions possibly acting as substrates for pol III transcription, identified 30 putative pol III type 3 transcriptional units. Notably, the subsequent detailed study of a selected subset of these units disclosed their tissue-specific expression and their involvement as key actors in relevant physiological and/or pathological processes, such as the regulation of alternative splicing, ion channel gating, GABA A function and restriction of tumor malignancy [2,5–7].

According to our results, more recent studies identified a remarkable number of pol III-transcribed ncRNAs by genome-wide location analysis of pol III machinery contributing to the growing appreciation of the widespread involvement of pol III in the expression of mammalian genomes [8–12]. Interestingly, based on their nucleotide sequence, the vast majority of the pol III type 3 transcriptional units identified so far does not seem to have murine orthologs, a condition that significantly limits the possible experimental approaches aimed to study their function.

In this work, we extend our PSE/DSE (Distal Sequence Elements)-based approach previously used in humans to the identification of the pol III type 3 transcriptome in mice with the final aim to identify pairs of mouse/human transcripts that display syntenic subchromosomal localization and, possibly, to find experimental evidence of their functional homology. To this aim, we searched for a comprehensive panel of murine pol III type 3 transcriptional units taking advantage of a recent bioinformatic algorithm able to screen the murine genome in search of genomic sequence stretches harboring the functional consensus sequences of type 3 promoters.

We identified 702 putative murine pol III transcriptional units whose analysis *in silico* supports the possible involvement in alternative splicing regulation and in the physio-pathology of the nervous system. Interestingly, by comparing this mouse collection with its human counterpart we also identified a set of 121 human/murine pairs of pol III type 3 transcriptional units that map in the corresponding subchromosomal localization within the specific hortolog genes, thus suggesting a possible functional homology. Exploiting a single pair of those identified as an experimental model, here we provide evidence for the mouse/human functional homology of two PSE/TATA-dependent transcriptional units with different nucleotide sequences.

## 2. Results and Discussion

### 2.1. The Screening *in silico* of the Mouse Genome Discloses a Large Number of Putative snRNA-Like Transcriptional Units That Map Preferentially in Intronic Regions of Protein-Coding Genes

**Figure 1:**
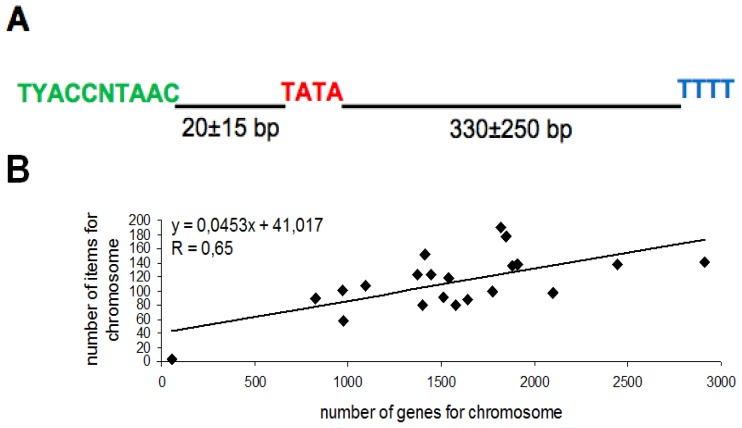
(**A**) 38B transcriptional unit and its functional elements: TYACCNTAAC, putative PSE; TATA, putative TATA box; (**B**) Significant positive correlation between the number of pol II-transcribed protein-coding genes and the novel transcriptional units in the murine chromosomes.

In order to design a PSE consensus sequence suitable for a bioinformatic search of type 3 promoters in the mouse genome, we aligned the PSE consensus sequences of three well-assessed human pol III-transcribed elements (H1, U6 and 7SK) with their murine counterparts. Since these consensus sequences are extremely conserved between the two species, to screen the mouse genome we used the human PSE consensus template together with the other parameters previously used for the search of the type 3 promoters in humans such as: (a) a PSE consensus sequence (TYACCNTAAC, obtained aligning H1, U6 and 7SK PSE sequences); (b) a PSE/TATA spacer (35 ± 25 nt); (c) a TATA box consensus sequence (TATA); (d) a transcribed portion length (350 ± 200 nt); and (e) a mammalian pol III transcription termination signal (TTTT) (Figure 1A). To perform our analysis we took advantage of COMPASSS, a recent software able to identify genomic regions characterized by the simultaneous occurrence of PSE, TATA and transcriptional termination sequences within specific nucleotide distances [13]. We found 2333 putative transcriptional units uniformly spread throughout the mouse genome (Table S1).

In order to verify the possible correlation between the presence of protein-coding genes and novel pol III transcriptional units, we crossed the number of items in the single chromosomes with the number of protein-coding genes that it contains. We found a significant positive correlation between the occurrence in the same genomic regions of pol II-transcribed protein-coding genes and pol III type 3 transcriptional units that overlap spatially (linear regression coefficient (*R*) = 0.65, degrees of freedom 19; *p* < 0.01) suggesting that protein-coding genes and pol III transcription units might be spatially correlated (Figure 1B, Table S2).

Next, we selected the subset of items that map in intronic portions of coding genes and, by using BLAST algorithm, we analyzed their subchromosomal localization. We found that 702 transcriptional units (out of 2333, (30.1%); Table S3) maps preferentially in protein-coding genes rather than in intergenic regions. This result suggests a functional correlation between the pol III type 3 transcriptional units and their protein-coding “host” genes (Chi-square test, *p* < 0.001; details on Chi-square test are shown in Tables S4 and S5).

This set of transcriptional units was further restricted since the elements were grouped in intronic and exonic transcription units by the means of their localization within the genomic portions of the protein-coding genes using the algorithm BLAST. We found that 61 out of 702 transcriptional units (8.7%) map in exons whereas 641 (91.3%) map in introns (results for each chromosome are shown in Table S6). Since the analysis by the Chi-square test revealed a positive correlation between introns and the novel transcription units (*p* < 0.005) (Table S7), we propose that the novel elements map preferentially within the intronic portions of the coding genes. Therefore, our analysis identified 702 possible transcription units that map in 531 coding genes (several protein-coding genes harbor more then one pol III transcriptional unit) whose possible functional activities deserve further investigation (Tables S8 and S9).

### 2.2. Significant Association to “Alternative Splicing” and “Brain” Functional Annotations of Coding Genes Hosting Pol III Transcriptional Units

Besides the subchromosomal localization we also tested our collection for possible recurrent functional annotations associated to the protein-coding genes that contain pol III transcriptional units. To this aim, we took advantage of the algorithm DAVID that allowed us to assess the possible enrichment in genes involved in the same cellular processes/molecular pathways and/or characterized by the same tissue specificity of expression [14,15]. Results, based on the most recurrent functional annotations obtained, demonstrated that the set of protein-coding genes hosting a pol III type 3 transcription units is particularly enriched in genes involved in or subjected to alternative splicing and expressed in brain (Tables 1 and S10). This result is relevant in light of our recent finding that several human pol III type 3 elements actively participate in alternative splicing regulation of the corresponding pol II-transcribed genes and are involved in processes that take place in the central nervous system [6,7]. Thus, considering the above results together with our recent findings, we propose an active role of pol III type 3 transcripts in the regulation of alternative splicing of the protein-coding genes in which they map, possibly contributing to physiopathological processes occurring in the nervous system.

### 2.3. 38B ncRNA Is the Murine Functional Homolog of the Human 38A ncRNA

By comparing the list of mouse intronic pol III transcriptional units with the corresponding human dataset [16] we found 121 protein-coding genes that host one or more pol III transcriptional units both in humans and in mice, suggesting that 17% of the human pol III type 3 transcripts may have a possible functional homolog in mouse (Table S11).

**Figure 2:**
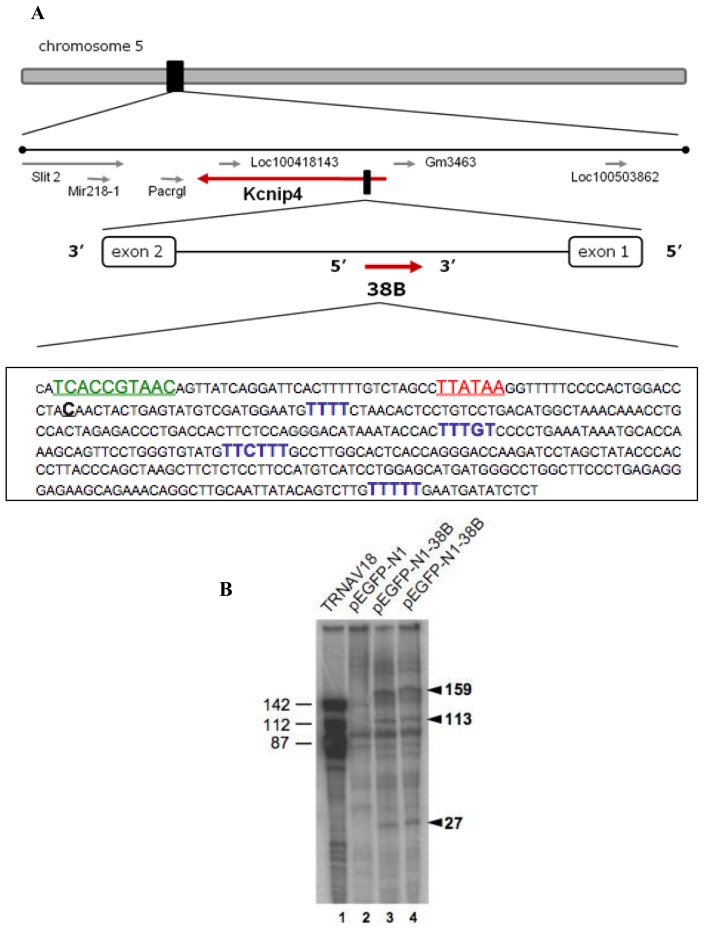
(**A**) Subchromosomal localization of the mouse 38B transcription unit. The functional consensus sequences are indicated in bold. Green, Proximal Sequence Element. Red, TATA box. Blue, Transcription Termination signals. The putative transcription 38B start site, located 29 bp downstream of the start of the TATA element, is also in bold and underlined; (**B**) *In vitro* transcription products of 38B. A HeLa cell nuclear extract was programmed with either pEGFP-N1 vector (lane 2), the same vector carrying the 38B transcription unit (lanes 3,4) or the human tRNA gene TRNAV18 carried by pGEM-T-Easy vector (lane 1). The length of 38B-specific transcripts (indicated by arrowheads on the right) was estimated on the basis of the migration positions of the three main TRNAV18 transcripts, generated by termination at different termination signals [17].

Next, we focused on the possible functional homology between human and murine pol III type3 transcriptional units. To test this hypothesis, we selected 38A (human) and 38B (murine) pol III-transcribed RNAs as possible pair with homology of function. 38A is a well characterized ncRNA that maps within intron 1 of KCNIP4 (Potassium Channel Interacting Protein 4) and plays a role in alternative splicing regulation of this protein in brain [6]. Similarly, 38B transcriptional unit maps within intron 1 of KCNIP4 (Potassium Channel Interacting Protein 4) gene in antisense configuration recapitulated the subchromosomal localization of its functionally active human counterpart 38A (Figure 2A). As shown in Figure 2B, *in vitro* transcription of plasmid-borne 38B by a HeLa cell nuclear extract, under conditions known to abolish pol II while preserving pol III transcription activity, produced three main specific transcripts (compare lanes 3–4 with lane 2) whose sizes, estimated on the basis of migration on the same gel of pre-tRNA^Val^ (AAC) transcripts of known size [17], perfectly matched those predicted for 38B transcripts starting at the A residue located 29 bp downstream of the TATA (underlined in Figure 2A) and ending at one of the first three termination signals (either canonical or noncanonical, in blue in Figure 2A[17]. The pol III-specific promoter and terminator elements of 38B thus appear to be efficiently recognized by the human pol III trasnscription machinery, with the production of ncRNAs with sizes ranging from 27 to 159 nucleotides. Notably, we have recently demonstrated that 38A, whose expression is upregulated by IL1-α, drives the synthesis of an alternative splicing form of KCNIP4. The production of this protein variant (variant C) is detrimental to brain physiology as it triggers the blockade of the fast kinetics of type A, voltage-dependent potassium channels, needed for brain physiology [18,19]. The analysis of the sequences of KCNIP4 alternative protein variants showed a very high homology between human and mouse thus suggesting that the splicing regulation driven by 38A RNA in humans might occur in mice driven by 38B RNA (Table S12).

### 2.4. The Expression of 38B RNA Is Specifically Associated to the Brain

**Figure 3:**
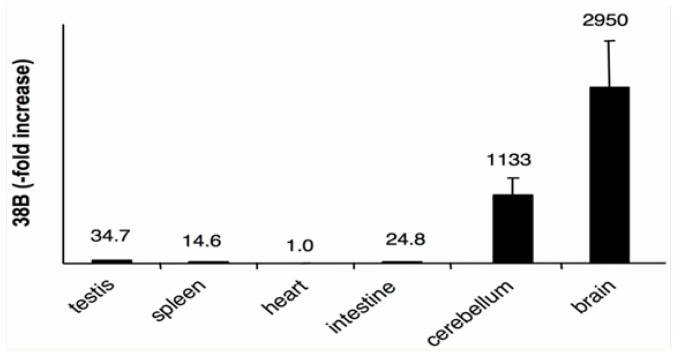
38B ncRNA expression in different mouse tissues. Results were normalized to the heart sample.

In order to test the expression of 38B ncRNA we measured by Real Time RT-PCR its amount in different tissues obtained from a pool of C57/BL6 mice. Results (after normalization to the heart sample) showed that 38B is poorly expressed in testis, intestine and spleen whereas its synthesis is strongly increased in cerebellum and in total brain extracts, matching with the expression pattern of KCNIP4 protein [20] (Figure 3). Therefore, the overlap of 38B tissue expression profile with KCNIP4 protein further suggest their possible synergistic action in mouse, as documented in humans.

### 2.5. 38B Expression Leads to the Synthesis of KCNIP4 Alternative Variant C in Mouse

**Figure 4:**
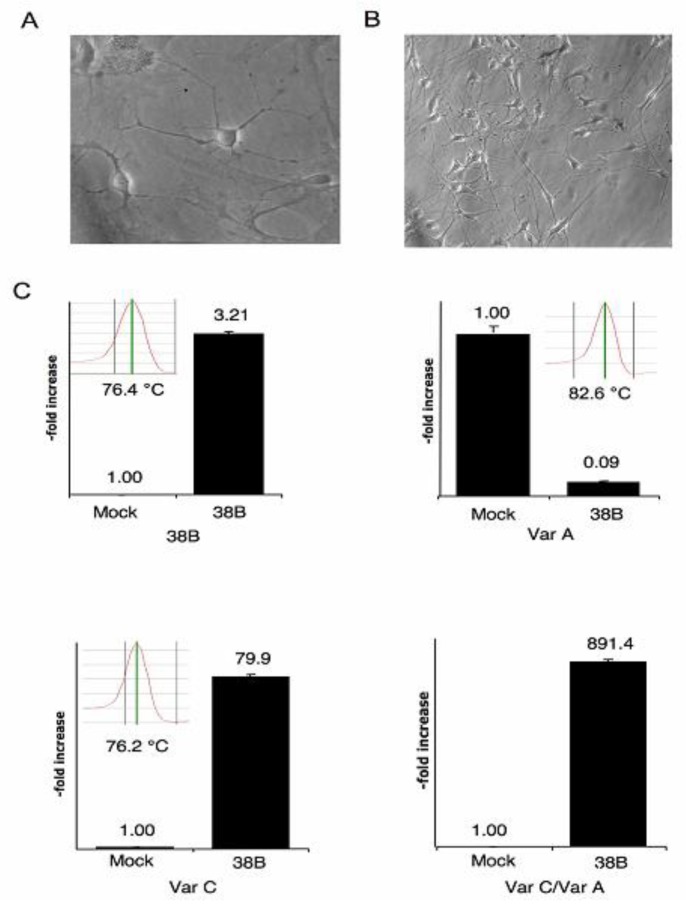
Hippocampal neurons after 6 (**A**) and 12 (**B**) days of *in vitro* culture; (**C**) The overexpression of 38B promotes the synthesis of an alternative KCNIP4 protein form as detected by Real Time RT-PCR analysis. Mock, pMock-transfected cells, 38B, p38B-transfected cells. The dissociation curves of the single amplification products are reported in the insets to demonstrate the single molecular species amplified.

In order to assess if the synthesis of 38B drives to KCNIP4 alternative splicing (thus recapitulating its human counterpart 38A) we analyzed the possibly increased expression of the alternative KCNIP4 variant C in hippocampal neurons overexpressing 38B RNA. To this aim, we generated a plasmid construct harboring the whole 38B transcription unit (hereafter referred to as p38B-EGFP-N1) together with a GFP cassette. The plasmids (and the empty vector pMock control) were transfected in primary cultures of hippocampal neurons and the amount of 38B ncRNA, KCNIP4 variants A and C were measured by Real Time RT-PCR in both samples (Figure 4A–C). Results showed that the overexpression of 38B drives to an unconventional unbalance of the amount of the two protein variants as the variant A (the ortholog of the human KCNIP4 canonical variant I) is strongly inhibited by the overexpression of 38B RNA, whereas the alternative variant C (the ortholog of the human alternative protein variant IV) is strongly increased in the same condition (Figure 4C). These data demonstrate that in primary cultures of murine hippocampal neurons, the overexpression of 38B intronic pol III-transcribed ncRNA in antisense configuration with respect to Kcnip4 intron 1 favors a splicing shift leading to the maturation of an alternative form of KCNIP4 endowed with a peculiar *N*-terminal fragment. Therefore, the effect of 38B expression on splicing regulation in mice recapitulates that of 38A in humans, and similar biological consequences are expected by its overexpression.

### 2.6. The Increased Transcription of 38B Affects Fast Inactivation of Mouse A-Type K^+^ Channels

In a previous work we demonstrated that the synthesis of the human KCNIP4 alternatively spliced Variant IV protein (promoted by the over expression of 38A RNA) in human neuroblastoma cells exerts a deep effect on their excitatory properties, abolishing the fast inactivation of A-type potassium channels. Indeed, in that work, we transfected transiently human cells with a plasmid construct expressing 38A ncRNA detecting the alternative splicing of KCNIP4 and the consequent abolishment of type A voltage-dependent current [6]. Since the alteration of these excitatory properties has been repeatedly associated to neurodegenerative processes [21] we here hypothesize that it may also drive to the perturbation of the excitatory properties of synapses in neurons.

**Figure 5:**
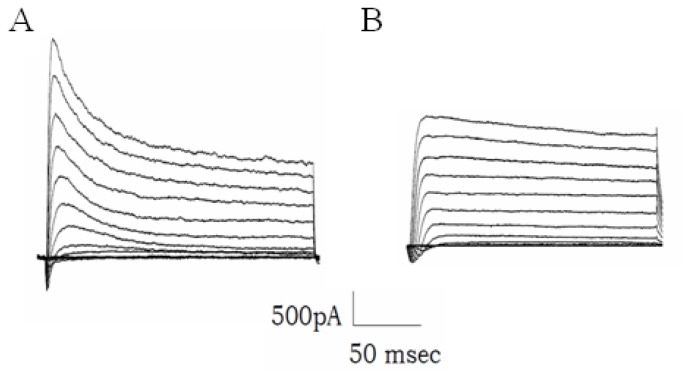
Expression of 38B eliminates fast inactivation of outward K^+^ current. Traces were obtained from a control neuron (**A**) and from a neuron transfected with 38B ncRNA (**B**). Current were evoked by 200 ms long voltage steps from −50 mV to +50 mV in 10 mV increments, after a prepulse at −100mV, starting from a holding potential of −100 mV

Therefore, in order to reproduce these results in a neuron, we measured the inactivation kinetics of A-type K channels in 38B-transfected primary cultures of mouse hippocampal cells and/or in pMock controls. Figure 5A,B show the outward potassium current elicited in a mock and in a 38B-transfected cell, respectively, as obtained in response to voltage steps from −50 mV to +50 mV starting from a holding potential of −100 mV. The fast inactivating component of the outward K current, representing about 30% in the mock K current, was decreased in the transfected cell, whereas the inactivation time constant estimated at +50 mV was found to be around 250 ms in the 38B cell, as opposed to the 55 msec of the control cell current. Similar results have been obtained in another six transfected cells, suggesting that 38B exerts in mice the same effect elicited by 38A in humans, and thereby strengthening the hypothesis of a mouse/human functional homology of type 3 ncRNAs.

## 3. Experimental Section

### 3.1. Screening of the Mouse Genome with COMPASSS and Identification of Pol III Type 3 Transcriptional Units

The screening of the mouse genome to search for putative pol III type 3 transcriptional units was carried out by means of the algorithm COMPASSS (COMplex Pattern of Sequence Search Software) [13]. The input sequences were the contigs of the 21 murine chromosomes obtained by the National Centre for Biotechnology Information (NCBI). In order to identify the pol III type 3 that map in pol II genes we used the algorithm Basic Local Alignment Search Tool (BLAST) under “Nucleotide”, database “Mouse genomic + transcript”, program “Megablast” [22].

### 3.2. Statistical Analysis

Data on genes number for each chromosome and data used in attended calculation in Chi-square test (Table S4) were all obtained by the NCBI databases.

### 3.3. Gene Annotations

Gene annotations were obtained through the Database for Annotation, Visualization, and Integrated Discovery (DAVID) [14,15]; tool “functional annotation chart”, background *Mus musculus*, threshold EASE 0,001. Gene list submitted was composed by the 531 genes containing pol III transcription units and 507 of them were recognized by DAVID as DAVID IDs.

### 3.4. 38B Plasmid Constructs Generation

In order to clone 38B transcriptional unit into pEGFP-N1 vector, the insert containing 38B (composed by the type 3 TATA/PSE-dependent promoter and the transcribed portion of 38B) was obtained amplifying 1442 bp of the murine genome with Forward oligo 5′-AGTCATTAATCCTAGATATCAAAAGGATGG-3′ and as reverse oligo 5′-AGTCATTAATTACAGATTTAGCATTATACTAA-3′. The insert was then digested with *Ase*I (New Labs) and subcloned into pEGFP-N1 plasmid vector (Clontech) following molecular cloning procedures described elsewhere [23].

### 3.5. *In vitro* Transcription

*In vitro* transcription reactions were carried out using a HeLa cell nuclear extract exactly as described [6], using 1.5 μg of template DNA in a final volume of 25 μL.

### 3.6. Hippocampal Neurons Preparation and Transfection

Hippocampal neurons were isolated from four seven-day-old mice C57/BL6. Hippocampi were removed, placed in 4 mL HBSS (bicarbonate-free Hank’s balanced salt solution, Invitrogen, cat.no.14185-052, Carlsbad, CA, USA) and 0.5 mL of 2.5% trypsin (Invitrogen, cat.no.15090-046, Carlsbad, CA, USA) and incubated for 15 min in a water bath at 37 °C. Next, trypsin solution was removed and the hippocampi washed gently two times for 5 min at room temperature. Hippocampi were dissociated by repeated pipetting in a Pasteur pipette and put in culture immediately onto 20 μg/mL polylysine-treated glass coverslip (for patch clamp experiments) and/or in a pretreated six-well plate (for RNA extraction). Neurons were plated in MEM, supplemented with glucose (0.6%) and containing 10% horse serum; after 1 day, once the cells were attached, the coverslip was transferred to a new plate with maintenance medium Neurobasal/B27, prepared according to the manufacturer’s instructions (supplementing Neurobasal medium (Invitrogen, cat.no.21103-049, Carlsbad, CA, USA) with l-Glutammine and B27 (Invitrogen, cat.no.17504-044, Carlsbad, CA, USA)). After 8 days of culture, the neurons were transfected using Lipofectamine 2000 (Invitrogen, cat.no.11668, Carlsbad, CA, USA) with pEGFPN1 (mock) and pEGFPN1-38B (38B) according to manufacturer’s instructions. The ratio used for transfection were (6:1:1) (μL lipofectamine: μg DNA: μL Plus reagent) in Neurobasal (1:200) (μg DNA: μL medium). The medium was removed after 7 h and replaced with complete Neurobasal/B27. 48 h after transfection neurons were analyzed by patch clamp and the RNA was extracted with TRIzol^®^ reagent (Invitrogen, Carlsbad, CA, USA) according manufacturer’s instructions.

### 3.7. Real Time Quantitative RT PCR Analysis

Total RNAs from samples (hippocampal neurons and mouse tissues (obtained by adult male C57BL/6 mice)) were extracted using TRIzol^®^ reagent (Invitrogen, Carlsbad, CA, USA) as described elsewhere [23] and subjected to reverse transcription by Transcriptor First Strand cDNA Synthesis Kit (Roche Applied Science, Mannheim, Germany) following manufacturer’s instructions. The total RNA from samples was measured by real-time quantitative RT-PCR using PE ABI PRISM@ 7700 Sequence Detection System (Perkin Elmer, Wellesley, MA, USA) and Sybr Green method following procedures previously described [24]. The sequences for mouse G3PDH (mG3PDH) forward and reverse primers were: 5′-TGTGTCCGTCGTGGATCTG-3′ and 5′-GATGCCTGCTTCACCACCTT-3′. The sequences for 38B forward and reverse primers were 5′-TGAGTATGTCGATGGAATG TTTTCTAA-3′ and 5′-GGGTCTCTAGTGGCAGGTTTGT-3′. The sequences for mouse KCNIP4 (Var A) forward and reverse primers were: 5′-GTGAGAAGGGTGGAAAGCATCT-3′ and 5′-AATGCT GCGCTTGGTGTTG-3′. The sequences for mouse KCNIP4 (Var C) forward and reverse primers were: 5′-GGAACAGTTTGGGCTGATTGA-3′ and 5′-GTAGCCATCTCCAGCTCATCTTC-3′. Relative transcript levels were determined from the relative standard curve constructed from stock cDNA dilutions, and divided by the target quantity of the calibrator as described elsewhere [23].

### 3.8. Electrophysiology

Total membrane currents were measured in the whole-cell configuration of the patch-clamp method as previously described [25]. Whole-cell voltage-clamp recordings were made using an Axopatch 200A amplifier (Axon Molecular Devices, Sunnyvale, CA, USA). Stimulation and acquisition were performed by a PC through a Digidata 1440 interface and Pclamp10 software (Molecular Devices). Traces were sampled at 5 kHz and low-pass filtered at 2 kHz. The standard extracellular bath solution contained (in mM): 140 NaCl, 5.4 KCl, 2 CaCl_2_, 1 MgCl_2_, 10 Glucose, 5 4-(2)hydrohyethyl)1-piperazineethanesulphonic acid (HEPES). The pH was adjusted at 7.35 with NaOH. The intracellular solution for contained (in mM): 142 KCl, 10 HEPES, 2 EGTA, 2 MgCl_2_ pH 7.3 with KOH.

The external solution was applied to the cell bath by gravity flow perfusion. The potassium currents were elicited from a holding potential of −100 mV by applying voltage pulses 200 ms long from −50 mV to +50 mV in 10 mV increments. The depolarizing voltage pulses of 200 ms duration was applied every 5 s. A best-fit exponential function was used to determine the time constant of the current inactivation.

Traces were analyzed by pClamp10 software (Clampfit) or by ANA, software developed by Michael Pusch, as previously described [25]. All subsequent analysis work and graphing was performed using Sigma Plot (SPSS Science, Chicago, IL, USA) sofware. Data are shown as mean ± standard error (SE).

## 4. Conclusions

One of the most relevant discoveries of the postgenomic era is that the mammalian genomes are almost entirely transcribed producing a panoply of different classes of noncoding RNAs with regulatory features [26–28]. In our previous works, we suggested that an unexpectedly high fraction of the human transcriptome might be synthesized by pol III, thus being subjected to pol III-specific regulatory features [2,3]. Our research approach was based on the identification of genomic regions endowed with pol III type 3 regulatory consensus sequences in specific configurations that can virtually compose functional pol III type 3 promoters. This first step was then followed by the experimental determination of the transcriptional activities of the promoters and by the detailed study of the function of the molecules of interest. As a proof of validity of this approach, our recent experimental studies demonstrated that all the ncRNAs so far studied were biologically active and involved in relevant regulatory processes of the cell [5–7]. In this work, taking advantage of recent software, we expand our analysis to the mouse genome, providing a comprehensive list of 702 murine transcriptional units. The bioinformatic analysis of their characteristics suggested functional homologies between the same transcripts in the two species. Interestingly, our results show that a high fraction of pol III transcriptional units map within introns of protein-coding genes whose biological functions are associated to the nervous system and, in particular, to brain physio-pathology. This finding is even more relevant in light of a second peculiar feature of these ncRNAs revealed by our recent experiments, such as their involvement in alternative splicing regulation [6,7]. Indeed, the concomitant enrichment of “splicing” and “brain” annotations in protein-coding genes that host pol III transcriptional units of our collection is in line with other works that indicate brain as a major site of alternative splicing events [29,30] and associates an active role of pol III type 3 transcription units with this process. Altogether, these findings suggest a novel scenario where RNA polymerase III affects the synthesis of proteins involved in alternative splicing, thus acting as an upstream regulator of gene expression. In this setting, even the fact that we identified at least one putative transcriptional unit in the 1.6% of murine protein-coding genes supports their possible splicing regulated by the novel pol III-transcribed ncRNAs.

A further relevant aspect of our work is the isolation and functional characterization of 38B in hippocampal neurons. Indeed, these experiments demonstrated that the pol III-dependent regulation of alternative splicing occurring in human KCNIP4 gene takes also place in mice, supporting the use of our promoter-based approach to identify mouse functional homologs of human type 3 transcripts also in the lack of sequence homology between human and mouse ncRNAs. This lack of homology, in spite of functional similarity, might be related to the intronic location of the pol III transcriptional units, and is in agreement with the recent suggestion that extended regions of human and mouse introns are involved in conserved functional links that do not depend on underlying sequence conservation [31].

Therefore, the experiments reported here show that our promoter-based approach is suitable for the identification of mouse functional homologs of human pol III-transcripts allowing the generation of mouse experimental models for the study of pol III elements.

In conclusion, besides the above mentioned functional studies, the mouse collection of pol III transcriptional units here provided is a valuable source for (1) the identification of novel small RNAs that affect splicing regulation and, (2) in light of the increasing evidence supporting the cell type/stage-specific regulation of pol III transcription, as a novel tool for gene-silencing presidium in therapy and research.

## Supplementary Materials



## Figures and Tables

**Table 1:** The most recurrent functional annotation genes hosting pol III transcriptional units. The table represent a schematic view of the output obtained by DAVID analysis. “Category” indicates the original database or resource where the terms orient; “Term” column lists the enriched terms associated with the gene list; “Count” and “%” are the number/percentage of the genes enriched in a term; “*p*-Value” indicates the statistical significance of the enrichment; “List total” is the number of the genes recognized by the Category; “Pop Hits” is the number of genes enriched by a term in a Category; “Pop Total” is the total number of genes in a Category; “Bonferroni” and “Benjamini” are two different coefficients that indicate the statistical significance of the enrichement respect to the Category.

Category	Term	Count	%	*p*-Value	List Total	Pop Hits	Pop Total	Bonferroni	Benjamini
SP_PIR_KEYWORDS	alternative splicing	227	44.77	4.64 × 10^−29^	464	4481	17854	1.48 × 10^−26^	1.48 × 10^−26^
UP_SEQ_FEATURE	splice variant	223	43.98	1.46 × 10^−24^	442	4448	16021	2.37 × 10^−21^	2.37 × 10^−21^
UP_TISSUE	Brain	273	53.85	1.44 × 10^−17^	493	7313	19868	2.28 × 10^−15^	2.28 × 10^−15^
UP_SEQ_FEATURE	domain: EGF-like 1	21	4.14	8.94 × 10^−12^	442	106	16021	1.45 × 10^−8^	7.23 × 10^−9^
SP_PIR_KEYWORDS	phosphoprotein	229	45.17	3.63 × 10^−10^	464	6311	17854	1.15 × 10^−7^	5.77 × 10^−8^
UP_SEQ_FEATURE	domain: EGF-like 2	17	3.35	3.65 × 10^−10^	442	79	16021	5.89 × 10^−7^	1.96 × 10^−7^
GOTERM_BP_FAT	GO:0007155 ~cell adhesion	43	8.48	5.00 × 10^−10^	352	561	13588	8.32 × 10^−7^	8.32 × 10^−7^
GOTERM_BP_FAT	GO:0022610 ~biological adhesion	43	8.48	5.30 × 10^−10^	352	562	13588	8.83 × 10^−7^	4.41 × 10^−7^
SP_PIR_KEYWORDS	egf-like domain	26	5.13	7.54 × 10^−10^	464	222	17854	2.40 × 10^−7^	7.99 × 10^−8^
UP_TISSUE	Cerebellum	80	15.78	1.33 × 10^−9^	493	1580	19868	2.10 × 10^−7^	1.05E × 10^−7^
UP_SEQ_FEATURE	compositionally biased region: Ser-rich	34	6.71	5.78 × 10^−9^	442	380	16021	9.35 × 10^−6^	2.34 × 10^−6^
