# Supplementary material for: The Murine PSE/TATA-Dependent Transcriptome: Evidence of Functional Homologies with Its Human Counterpart
Source: Int J Mol Sci. 2012 Nov 13;13(11):14813–27. doi: 10.3390/ijms131114813 (PMC3509611; doi:10.3390/ijms131114813)
Supplement: Supplementary file 1 [file ijms-13-14813-s001.pdf]

Putative pol III type 3 transcription units identified by COMPASSS

CHR	PROMOTERS	Direction	position in Contig	from	to	Position in chromosome	Length	TATA distance	poly-T distance
			NT_039169.7 (1)						
1	0	5'-3'	811630	811630	812004	3.811.630	374	10	364
	1	5'-3'	1590858	1590858	1591301	4.590.858	443	24	419
	2	5'-3'	1994160	1994160	1994497	4.994.160	337	6	327
	3	5'-3'	6527800	6527800	6528025	9.527.800	225	10	215
	4	5'-3'	8682780	8682780	8683043	11.682.780	263	16	253
	5	5'-3'	8745619	8745619	8745996	11.745.619	377	13	367
	6	5'-3'	11727442	11727442	11728016	14.727.442	574	28	564
	7	5'-3'	14263375	14263375	14263763	17.263.375	388	12	378
	8	5'-3'	16442270	16442270	16442708	19.442.270	438	12	428
	9	5'-3'	18841135	18841135	18841497	21.841.135	362	26	352
	10	3'-5'	2775186	2775382	2775819	5.775.186	437	20	427
	11	3'-5'	3993842	3994000	3994475	6.993.842	475	24	465
	12	3'-5'	6596021	6596163	6596654	9.596.021	491	8	481
	13	3'-5'	7061605	7061823	7062238	10.061.605	415	6	405
	14	3'-5'	7105421	7105466	7106054	10.105.421	588	21	578
	15	3'-5'	13717998	13718323	13718631	16.717.998	308	18	298
	16	3'-5'	16915979	16916131	16916612	19.915.979	481	9	471
	17	3'-5'	16942929	16943195	16943562	19.942.929	367	27	357
	18	3'-5'	17101314	17101456	17101947	20.101.314	491	30	481
			NT_039170.7 (2)						
	19	5'-3'	2321295	2321295	2321704	24.794.644	409	20	399
	20	5'-3'	2528703	2528703	2529112	25.002.052	409	10	399
	21	5'-3'	5830337	5830337	5830706	28.303.686	369	15	359
	22	5'-3'	5958024	5958024	5958404	28.431.373	380	21	370
	23	5'-3'	6171558	6171558	6171882	28.644.907	324	10	314
	24	5'-3'	6908469	6908469	6908796	29.381.818	327	29	317
	25	5'-3'	7205589	7205589	7205841	29.678.938	252	11	242
	26	5'-3'	13983077	13983077	13983552	36.456.426	475	14	465
	27	5'-3'	15852248	15852248	15852385	38.325.597	137	20	127
	28	5'-3'	18627231	18627231	18627507	41.100.580	276	27	266
	29	5'-3'	24419418	24419418	24419808	46.892.767	390	18	380

30	5'-3'	25983641	25983641	25983995	48.456.990	354	24	344
31	5'-3'	26669680	26669680	26670120	49.143.029	440	12	430
32	5'-3'	28431149	28431149	28431527	50.904.498	378	27	368
33	5'-3'	28758146	28758146	28758471	51.231.495	325	7	315
34	5'-3'	30187775	30187775	30188240	52.661.124	465	8	455
35	5'-3'	30350985	30350985	30351229	52.824.334	244	16	234
36	5'-3'	32095500	32095500	32095893	54.568.849	393	12	383
37	5'-3'	35865919	35865919	35866135	58.339.268	216	8	206
38	5'-3'	36902354	36902354	36902770	59.375.703	416	28	406
39	5'-3'	43027177	43027177	43027573	65.500.526	396	30	386
40	5'-3'	46302959	46302959	46303204	68.776.308	245	17	235
41	5'-3'	46550222	46550222	46550594	69.023.571	372	28	362
42	5'-3'	47391807	47391807	47392125	69.865.156	318	21	308
43	5'-3'	48135554	48135554	48135906	70.608.903	352	16	342
44	5'-3'	51000544	51000544	51000878	73.473.893	334	6	324
45	5'-3'	52611028	52611028	52611561	75.084.377	533	25	523
46	3'-5'	733065	733348	733698	23.206.414	350	8	340
47	3'-5'	1287475	1287756	1288108	23.760.824	352	9	342
48	3'-5'	2979625	2979823	2980258	25.452.974	435	7	425
49	3'-5'	3016427	3016673	3017060	25.489.776	387	13	377
50	3'-5'	6436601	6436869	6437234	28.909.950	365	21	355
51	3'-5'	7071324	7071373	7071957	29.544.673	584	12	574
52	3'-5'	9696594	9696842	9697227	32.169.943	385	32	375
53	3'-5'	10282017	10282408	10282650	32.755.366	242	11	232
54	3'-5'	15798536	15798799	15799169	38.271.885	370	32	360
55	3'-5'	17070954	17071413	17071587	39.544.303	174	15	164
56	3'-5'	19957482	19957740	19958115	42.430.831	375	26	365
57	3'-5'	23377182	23377417	23377815	45.850.531	398	26	388
58	3'-5'	25395628	25395990	25396261	47.868.977	271	22	261
59	3'-5'	26530408	26530420	26531041	49.003.757	621	16	611
60	3'-5'	27896063	27896345	27896696	50.369.412	351	30	341
61	3'-5'	29834518	29834826	29835151	52.307.867	325	15	315
62	3'-5'	33343144	33343409	33343777	55.816.493	368	27	358
63	3'-5'	35580167	35580564	35580800	58.053.516	236	34	226
64	3'-5'	36114049	36114100	36114682	58.587.398	582	20	572
65	3'-5'	38261440	38261772	38262073	60.734.789	301	17	291
66	3'-5'	38784565	38784843	38785198	61.257.914	355	11	345

67	3'-5'	43504822	43505085	43505455	65.978.171	370	20	360
68	3'-5'	46555208	46555446	46555841	69.028.557	395	30	385
		NT_039173.7 (3)						
69	5'-3'	5437836	5437836	5438165	80.555.966	329	18	319
70	5'-3'	5965367	5965367	5965771	81.083.497	404	23	394
71	3'-5'	1290490	1290735	1291123	76.408.620	388	29	378
72	3'-5'	1561011	1561099	1561644	76.679.141	545	16	535
73	3'-5'	6899658	6899899	6900291	82.017.788	392	33	382
74	3'-5'	8171212	8171510	8171845	83.289.342	335	25	325
75	3'-5'	8503212	8503341	8503845	83.621.342	504	25	494
76	3'-5'	8969319	8969787	8969952	84.087.449	165	17	155
77	3'-5'	9073329	9073513	9073962	84.191.459	449	10	439
		NT_078297.6 (4)						
78	5'-3'	3018007	3018007	3018392	90.361.685	385	9	375
79	5'-3'	11135600	11135600	11135948	98.479.278	348	26	338
80	5'-3'	11142270	11142270	11142653	98.485.948	383	19	373
81	5'-3'	11796126	11796126	11796480	99.139.804	354	17	344
82	5'-3'	13530919	13530919	13531318	100.874.597	399	7	389
83	5'-3'	16982707	16982707	16983089	104.326.385	382	6	372
84	5'-3'	17466868	17466868	17467266	104.810.546	398	14	388
85	5'-3'	20279056	20279056	20279479	107.622.734	423	16	413
86	5'-3'	24249506	24249506	24249866	111.593.184	360	10	350
87	5'-3'	25354694	25354694	25355031	112.698.372	337	18	327
88	5'-3'	26430227	26430227	26430607	113.773.905	380	27	370
89	5'-3'	29015781	29015781	29016309	116.359.459	528	26	518
90	5'-3'	30090610	30090610	30091082	117.434.288	472	14	462
91	5'-3'	31168397	31168397	31168792	118.512.075	395	9	385
92	5'-3'	31662105	31662105	31662454	119.005.783	349	7	339
93	5'-3'	39253637	39253637	39253916	126.597.315	279	23	269
94	5'-3'	42402246	42402246	42402461	129.745.924	215	26	205
95	5'-3'	44161218	44161218	44161595	131.504.896	377	16	367
96	5'-3'	44344146	44344146	44344545	131.687.824	399	17	389
97	5'-3'	46091036	46091036	46091336	133.434.714	300	16	290
98	5'-3'	57318427	57318427	57318842	144.662.105	415	11	405
99	5'-3'	59384901	59384901	59385248	146.728.579	347	25	337

100	5'-3'	60198016	60198016	60198365	147.541.694	349	6	339
101	5'-3'	60586519	60586519	60586740	147.930.197	221	18	211
102	5'-3'	61219018	61219018	61219404	148.562.696	386	24	376
103	5'-3'	61781610	61781610	61782037	149.125.288	427	7	417
104	5'-3'	67124785	67124785	67125070	154.468.463	285	6	275
105	5'-3'	67268125	67268125	67268513	154.611.803	388	8	378
106	3'-5'	807011	807262	807644	88.150.689	382	17	372
107	3'-5'	1149767	1150025	1150400	88.493.445	375	24	365
108	3'-5'	2068443	2068559	2069076	89.412.121	517	12	507
109	3'-5'	7831391	7831645	7832024	95.175.069	379	33	369
110	3'-5'	10583143	10583364	10583776	97.926.821	412	16	402
111	3'-5'	12274852	12275195	12275485	99.618.530	290	14	280
112	3'-5'	15008747	15008994	15009380	102.352.425	386	34	376
113	3'-5'	16341922	16342159	16342555	103.685.600	396	19	386
114	3'-5'	16759953	16760109	16760586	104.103.631	477	27	467
115	3'-5'	17188436	17188692	17189069	104.532.114	377	26	367
116	3'-5'	20700743	20701065	20701376	108.044.421	311	25	301
117	3'-5'	22525041	22525297	22525674	109.868.719	377	9	367
118	3'-5'	22529493	22529746	22530126	109.873.171	380	27	370
119	3'-5'	23014579	23014909	23015212	110.358.257	303	7	293
120	3'-5'	23628453	23628709	23629086	110.972.131	377	22	367
121	3'-5'	24070316	24070484	24070949	111.413.994	465	11	455
122	3'-5'	24138116	24138372	24138749	111.481.794	377	21	367
123	3'-5'	25682715	25683036	25683348	113.026.393	312	6	302
124	3'-5'	26999357	26999589	26999990	114.343.035	401	28	391
125	3'-5'	31066775	31067075	31067408	118.410.453	333	6	323
126	3'-5'	31771799	31771864	31772432	119.115.477	568	11	558
127	3'-5'	32212262	32212474	32212895	119.555.940	421	24	411
128	3'-5'	35377252	35377411	35377885	122.720.930	474	22	464
129	3'-5'	36193419	36193675	36194052	123.537.097	377	28	367
130	3'-5'	37305811	37306086	37306444	124.649.489	358	13	348
131	3'-5'	38292184	38292657	38292817	125.635.862	160	24	150
132	3'-5'	38529533	38529624	38530166	125.873.211	542	6	532
133	3'-5'	43621838	43622025	43622471	130.965.516	446	17	436
134	3'-5'	44117705	44117941	44118338	131.461.383	397	21	387
135	3'-5'	48917795	48918044	48918428	136.261.473	384	22	374
136	3'-5'	59860433	59860679	59861066	147.204.111	387	15	377

137	3'-5'	59896121	59896408	59896754	147.239.799	346	9	336
138	3'-5'	60774502	60774761	60775135	148.118.180	374	26	364
139	3'-5'	61042288	61042662	61042921	148.385.966	259	21	249
140	3'-5'	62136759	62137043	62137392	149.480.437	349	20	339
141	3'-5'	63584931	63585226	63585564	150.928.609	338	22	328
142	3'-5'	65170791	65170916	65171424	152.514.469	508	11	498
143	3'-5'	65484878	65485171	65485511	152.828.556	340	19	330
144	3'-5'	66413318	66413427	66413951	153.756.996	524	18	514
145	3'-5'	67202457	67202796	67203090	154.546.135	294	17	284
		NT_039185.7 (5)						
146	5'-3'	2468491	2468491	2468813	160.567.364	322	22	312
147	5'-3'	2776505	2776505	2776874	160.875.378	369	30	359
148	5'-3'	3345383	3345383	3345665	161.444.256	282	22	272
149	5'-3'	4925889	4925889	4926211	163.024.762	322	8	312
150	5'-3'	7442654	7442654	7443074	165.541.527	420	19	410
151	5'-3'	11498234	11498234	11498815	169.597.107	581	12	571
152	5'-3'	14964037	14964037	14964345	173.062.910	308	31	298
153	5'-3'	15586549	15586549	15587072	173.685.422	523	10	513
154	5'-3'	15683228	15683228	15683608	173.782.101	380	10	370
155	5'-3'	17056439	17056439	17056916	175.155.312	477	9	467
156	5'-3'	17099038	17099038	17099368	175.197.911	330	24	320
157	5'-3'	17925122	17925122	17925369	176.023.995	247	21	237
158	5'-3'	18068331	18068331	18068753	176.167.204	422	23	412
159	5'-3'	18894849	18894849	18895211	176.993.722	362	9	352
160	5'-3'	19947673	19947673	19948045	178.046.546	372	9	362
161	5'-3'	20231069	20231069	20231504	178.329.942	435	12	425
162	5'-3'	21743378	21743378	21743783	179.842.251	405	7	395
163	5'-3'	22059497	22059497	22059726	180.158.370	229	13	219
164	5'-3'	22227748	22227748	22228189	180.326.621	441	13	431
165	5'-3'	23323055	23323055	23323443	181.421.928	388	28	378
166	3'-5'	1364423	1364656	1365056	159.463.296	400	8	390
167	3'-5'	1574783	1574986	1575416	159.673.656	430	12	420
168	3'-5'	1841968	1842351	1842601	159.940.841	250	28	240
169	3'-5'	2142679	2142690	2143312	160.241.552	622	28	612
170	3'-5'	3345686	3345948	3346319	161.444.559	371	30	361
171	3'-5'	4159283	4159558	4159916	162.258.156	358	15	348

	172	3'-5'	5403637	5403775	5404270	163.502.510	495	30	485
	173	3'-5'	8040034	8040263	8040667	166.138.907	404	6	394
	174	3'-5'	12749459	12749742	12750092	170.848.332	350	34	340
	175	3'-5'	17478722	17478997	17479355	175.577.595	358	30	348
	176	3'-5'	18253598	18253775	18254231	176.352.471	456	20	446
	177	3'-5'	18275142	18275347	18275775	176.374.015	428	23	418
	178	3'-5'	23477539	23477936	23478172	181.576.412	236	22	226
	179	3'-5'	23898896	23899134	23899529	181.997.769	395	23	385
	180	3'-5'	25435080	25435364	25435713	183.533.953	349	24	339
			NT_039189.7 (6)						
	181	5'-3'	218012	218012	218442	185.545.822	430	9	420
	182	5'-3'	926385	926385	926846	186.254.195	461	13	451
	183	5'-3'	5012865	5012865	5013189	190.340.675	324	23	314
	184	3'-5'	92925	93066	93558	185.420.735	492	27	482
	185	3'-5'	3105530	3105783	3106163	188.433.340	380	16	370
	186	3'-5'	3132476	3132788	3133109	188.460.286	321	21	311
	187	3'-5'	4906910	4907138	4907543	190.234.720	405	7	395
			NT_039190.7 (7)						
	188	5'-3'	974477	974477	974693	194.850.134	216	24	206
	189	3'-5'	1602604	1602805	1603237	195.478.261	432	17	422
2			NT_039202.7 (1)					tata Distance	olyt Distanc
	0	5'-3'	1170630	1170630	1171221	4170630	591	23	581
	1	5'-3'	1885355	1885355	1885755	4885355	400	24	390
	2	5'-3'	6617614	6617614	6617775	9617614	161	29	151
	3	5'-3'	7671395	7671395	7671745	10671395	350	28	340
	4	5'-3'	8910816	8910816	8911326	11910816	510	7	500
	5	5'-3'	9247296	9247296	9247595	12247296	299	30	289
	6	5'-3'	9579109	9579109	9579464	12579109	355	14	345
	7	5'-3'	11488978	11488978	11489450	14488978	472	26	462
	8	5'-3'	11963053	11963053	11963307	14963053	254	14	244
	9	5'-3'	17355591	17355591	17355929	20355591	338	11	328
	10	3'-5'	3275100	3275274	3275733	6275100	459	28	449
	11	3'-5'	4347002	4347256	4347635	7347002	379	34	369
	12	3'-5'	5424072	5424360	5424705	8424072	345	29	335

13	3'-5'	5441498	5441746	5442131	8441498	385	9	375
14	3'-5'	5625799	5626153	5626432	8625799	279	19	269
15	3'-5'	12463920	12464184	12464553	15463920	369	26	359
16	3'-5'	13332982	13333208	13333615	16332982	407	11	397
17	3'-5'	13894723	13894942	13895356	16894723	414	28	404
18	3'-5'	13896540	13896795	13897173	16896540	378	33	368
19	3'-5'	15412400	15412719	15413033	18412400	314	14	304
20	3'-5'	18122616	18122862	18123249	21122616	387	32	377
21	3'-5'	18981057	18981287	18981690	21981057	403	11	393
		NT_039206.7 (2)						
22	5'-3'	429346	429346	429697	22826598	351	26	341
23	5'-3'	11643382	11643382	11643836	34040634	454	27	444
24	5'-3'	11982916	11982916	11983213	34380168	297	21	287
25	5'-3'	12675385	12675385	12675751	35072637	366	27	356
26	5'-3'	16627156	16627156	16627630	39024408	474	20	464
27	5'-3'	21011867	21011867	21012266	43409119	399	10	389
28	5'-3'	24283655	24283655	24283945	46680907	290	29	280
29	5'-3'	24433576	24433576	24433950	46830828	374	11	364
30	5'-3'	24516869	24516869	24517302	46914121	433	22	423
31	5'-3'	25022558	25022558	25023110	47419810	552	30	542
32	5'-3'	25566796	25566796	25567125	47964048	329	12	319
33	5'-3'	31388397	31388397	31388808	53785649	411	7	401
34	5'-3'	33593125	33593125	33593511	55990377	386	30	376
35	5'-3'	35391493	35391493	35391820	57788745	327	22	317
36	3'-5'	4903447	4903872	4904080	27300699	208	30	198
37	3'-5'	8178324	8178356	8178957	30575576	601	18	591
38	3'-5'	13938937	13939178	13939570	36336189	392	31	382
39	3'-5'	16966224	16966290	16966857	39363476	567	34	557
40	3'-5'	20320821	20321163	20321454	42718073	291	17	281
41	3'-5'	20383332	20383630	20383965	42780584	335	31	325
42	3'-5'	20646212	20646493	20646845	43043464	352	32	342
43	3'-5'	24011581	24011762	24012214	46408833	452	27	442
44	3'-5'	24501459	24501757	24502092	46898711	335	11	325
45	3'-5'	27032085	27032363	27032718	49429337	355	12	345
46	3'-5'	28128630	28128664	28129263	50525882	599	29	589

47	3'-5'	29674453	29674764	29675086	52071705	322	29	312
48	3'-5'	32119633	32119928	32120266	54516885	338	12	328
		NT_039207.7 (3)						
49	5'-3'	2352594	2352594	2352996	61311292	402	16	392
50	5'-3'	6896480	6896480	6896745	65855178	265	8	255
51	5'-3'	7714540	7714540	7714911	66673238	371	30	361
52	5'-3'	11242540	11242540	11242925	70201238	385	19	375
53	5'-3'	11597094	11597094	11597344	70555792	250	24	240
54	5'-3'	13217594	13217594	13217975	72176292	381	9	371
55	5'-3'	18864873	18864873	18865359	77823571	486	16	476
56	5'-3'	19214284	19214284	19214679	78172982	395	11	385
57	5'-3'	24201512	24201512	24202009	83160210	497	19	487
58	5'-3'	26690966	26690966	26691177	85649664	211	9	201
59	5'-3'	28223413	28223413	28223793	87182111	380	20	370
60	5'-3'	28455454	28455454	28455880	87414152	426	30	416
61	5'-3'	30050000	30050000	30050391	89008698	391	13	381
62	5'-3'	30820532	30820532	30820978	89779230	446	29	436
63	5'-3'	33273327	33273327	33273522	92232025	195	22	185
64	5'-3'	39136879	39136879	39137236	98095577	357	6	347
65	5'-3'	40863135	40863135	40863466	99821833	331	6	321
66	5'-3'	43047480	43047480	43047813	102006178	333	9	323
67	5'-3'	45313693	45313693	45313998	104272391	305	11	295
68	5'-3'	47580897	47580897	47581285	106539595	388	10	378
69	5'-3'	48272733	48272733	48273160	107231431	427	15	417
70	5'-3'	48894075	48894075	48894493	107852773	418	12	408
71	5'-3'	50212604	50212604	50212994	109171302	390	17	380
72	5'-3'	50429210	50429210	50429496	109387908	286	12	276
73	5'-3'	52198414	52198414	52198870	111157112	456	19	446
74	5'-3'	56602457	56602457	56602740	115561155	283	29	273
75	5'-3'	57869351	57869351	57869708	116828049	357	16	347
76	5'-3'	59954530	59954530	59954944	118913228	414	15	404
77	5'-3'	60423891	60423891	60424265	119382589	374	10	364
78	5'-3'	62133947	62133947	62134330	121092645	383	15	373
79	5'-3'	64758203	64758203	64758576	123716901	373	21	363
80	5'-3'	69769192	69769192	69769567	128727890	375	15	365

81	5'-3'	72032379	72032379	72032622	130991077	243	18	233
82	5'-3'	81887753	81887753	81888152	140846451	399	17	389
83	5'-3'	84374992	84374992	84375382	143333690	390	9	380
84	5'-3'	84508006	84508006	84508197	143466704	191	14	181
85	5'-3'	85570454	85570454	85570610	144529152	156	8	146
86	5'-3'	95978399	95978399	95978792	154937097	393	17	383
87	5'-3'	99469120	99469120	99469590	158427818	470	9	460
88	5'-3'	99525716	99525716	99526041	158484414	325	26	315
89	5'-3'	102626423	102626423	102626953	161585121	530	17	520
90	3'-5'	1952148	1952155	1952781	60910846	626	26	616
91	3'-5'	3116018	3116254	3116651	62074716	397	30	387
92	3'-5'	7587468	7587783	7588101	66546166	318	7	308
93	3'-5'	7595198	7595422	7595831	66553896	409	25	399
94	3'-5'	7717751	7717944	7718384	66676449	440	20	430
95	3'-5'	9416256	9416519	9416889	68374954	370	34	360
96	3'-5'	9503896	9504128	9504529	68462594	401	11	391
97	3'-5'	11384628	11384925	11385261	70343326	336	32	326
98	3'-5'	15677270	15677523	15677903	74635968	380	32	370
99	3'-5'	18568574	18568856	18569207	77527272	351	26	341
100	3'-5'	18602623	18602771	18603256	77561321	485	20	475
101	3'-5'	22546040	22546345	22546673	81504738	328	16	318
102	3'-5'	22625413	22625774	22626046	81584111	272	14	262
103	3'-5'	22995857	22996096	22996490	81954555	394	19	384
104	3'-5'	26800572	26800921	26801205	85759270	284	11	274
105	3'-5'	36239784	36239800	36240417	95198482	617	33	607
106	3'-5'	37535118	37535343	37535751	96493816	408	12	398
107	3'-5'	41085494	41085768	41086127	100044192	359	22	349
108	3'-5'	42620570	42620814	42621203	101579268	389	11	379
109	3'-5'	42933211	42933541	42933844	101891909	303	7	293
110	3'-5'	47002297	47002517	47002930	105960995	413	20	403
111	3'-5'	49125723	49125936	49126356	108084421	420	18	410
112	3'-5'	55323935	55324201	55324568	114282633	367	8	357
113	3'-5'	55901788	55902059	55902421	114860486	362	6	352
114	3'-5'	60269706	60269999	60270339	119228404	340	16	330
115	3'-5'	60814048	60814315	60814681	119772746	366	15	356
116	3'-5'	62564282	62564511	62564915	121522980	404	23	394
117	3'-5'	64114577	64114842	64115210	123073275	368	16	358

	118	3'-5'	70309400	70309827	70310033	129268098	206	6	196
	119	3'-5'	74442054	74442407	74442687	133400752	280	10	270
	120	3'-5'	75063892	75064321	75064525	134022590	204	7	194
	121	3'-5'	75579627	75580080	75580260	134538325	180	12	170
	122	3'-5'	83395519	83395809	83396152	142354217	343	10	333
	123	3'-5'	84002067	84002473	84002700	142960765	227	7	217
	124	3'-5'	93350031	93350337	93350664	152308729	327	33	317
	125	3'-5'	98133889	98134170	98134522	157092587	352	22	342
	126	3'-5'	98542697	98543004	98543330	157501395	326	26	316
	127	3'-5'	100939374	100939638	100940007	159898072	369	8	359
	128	3'-5'	102699207	102699353	102699840	161657905	487	32	477
	129	3'-5'	103590927	103591178	103591560	162549625	382	20	372
	130	3'-5'	104502665	104502903	104503298	163461363	395	22	385
	131	3'-5'	106504414	106504608	106505047	165463112	439	28	429
	132	3'-5'	109684511	109684714	109685144	168643209	430	15	420
	133	3'-5'	109890591	109890735	109891224	168849289	489	21	479
	134	3'-5'	111097488	111097670	111098121	170056186	451	15	441
	135	3'-5'	112560930	112561189	112561563	171519628	374	8	364
	136	3'-5'	114533195	114533464	114533828	173491893	364	22	354
			NT_039212.6 (6)						
	137	3'-5'	3327692	3328026	3328325	180776484	299	17	289
3			NT_078380.6 (1)						
	0	5'-3'	1835075	1835075	1835454	4835075	379	20	369
	1	5'-3'	2290623	2290623	2291126	5290623	503	27	493
	2	5'-3'	4530446	4530446	4530645	7530446	199	24	189
	3	5'-3'	5042316	5042316	5042915	8042316	599	20	589
	4	5'-3'	6598377	6598377	6598525	9598377	148	29	138
	5	3'-5'	767816	768070	768449	3767816	379	10	369
	6	3'-5'	2324427	2324651	2325060	5324427	409	22	399
	7	3'-5'	3500564	3500793	3501197	6500564	404	21	394
	8	3'-5'	5464982	5465381	5465615	8464982	234	34	224
	9	3'-5'	8841511	8841842	8842144	11841511	302	18	292
	10	3'-5'	12418056	12418556	12418689	15418056	133	17	123
	11	3'-5'	12549665	12550165	12550298	15549665	133	17	123

		NT_162143.3 (2)					
12	5'-3'	1701355	1701355	1701807	17362045	452	18	442
13	5'-3'	2241892	2241892	2242277	17902582	385	7	375
14	5'-3'	2285654	2285654	2286033	17946344	379	9	369
15	5'-3'	5081682	5081682	5082016	20742372	334	21	324
16	5'-3'	7051224	7051224	7051481	22711914	257	31	247
17	5'-3'	16617583	16617583	16618047	32278273	464	11	454
18	5'-3'	17808798	17808798	17809318	33469488	520	9	510
19	5'-3'	19534384	19534384	19534731	35195074	347	7	337
20	5'-3'	20894310	20894310	20894754	36555000	444	13	434
21	5'-3'	21198679	21198679	21198900	36859369	221	25	211
22	5'-3'	23738106	23738106	23738467	39398796	361	29	351
23	5'-3'	24273160	24273160	24273488	39933850	328	17	318
24	3'-5'	1345597	1345984	1346230	17006287	246	24	236
25	3'-5'	2095751	2096071	2096384	17756441	313	32	303
26	3'-5'	3818087	3818502	3818720	19478777	218	27	208
27	3'-5'	6797041	6797304	6797674	22457731	370	21	360
28	3'-5'	9129332	9129571	9129965	24790022	394	17	384
29	3'-5'	9909809	9910101	9910442	25570499	341	9	331
30	3'-5'	12038390	12038657	12039023	27699080	366	34	356
31	3'-5'	13449272	13449530	13449905	29109962	375	7	365
32	3'-5'	14410990	14411251	14411623	30071680	372	18	362
33	3'-5'	17546497	17546811	17547130	33207187	319	16	309
34	3'-5'	18360713	18361216	18361346	34021403	130	22	120
35	3'-5'	19996347	19996561	19996980	35657037	419	26	409
36	3'-5'	21593376	21593396	21594009	37254066	613	12	603
37	3'-5'	21959513	21959840	21960146	37620203	306	24	296
38	3'-5'	22725972	22726042	22726605	38386662	563	30	553
39	3'-5'	23251920	23252186	23252553	38912610	367	13	357
40	3'-5'	23482926	23482957	23483559	39143616	602	7	592
41	3'-5'	23561117	23561359	23561750	39221807	391	19	381
		NT_039229.7 (3)						
42	5'-3'	1454384	1454384	1454763	41908923	379	8	369
43	5'-3'	4133616	4133616	4133956	44588155	340	15	330
44	5'-3'	6691979	6691979	6692358	47146518	379	13	369
45	5'-3'	8370419	8370419	8370788	48824958	369	24	359

46	3'-5'	179983	180229	180616	40634522	387	17	377
47	3'-5'	2378422	2378682	2379055	42832961	373	28	363
48	3'-5'	3164413	3164669	3165046	43618952	377	17	367
49	3'-5'	6018765	6019013	6019398	46473304	385	21	375
50	3'-5'	7616749	7616990	7617382	48071288	392	20	382
51	3'-5'	8499997	8500264	8500630	48954536	366	19	356
		NT_166285.1 (5)						
52	5'-3'	2400538	2400538	2400689	133526974	151	16	141
53	5'-3'	2421254	2421254	2421847	133547690	593	20	583
54	5'-3'	4496009	4496009	4496400	135622445	391	24	381
55	5'-3'	6270778	6270778	6271221	137397214	443	17	433
56	5'-3'	8420990	8420990	8421434	139547426	444	13	434
57	5'-3'	8648866	8648866	8649266	139775302	400	14	390
58	5'-3'	10450262	10450262	10450615	141576698	353	6	343
59	5'-3'	16448991	16448991	16449381	147575427	390	8	380
60	5'-3'	23341363	23341363	23341812	154467799	449	15	439
61	5'-3'	24679586	24679586	24680005	155806022	419	15	409
62	5'-3'	25289552	25289552	25289891	156415988	339	10	329
63	5'-3'	27629202	27629202	27629584	158755638	382	28	372
64	3'-5'	1585626	1585799	1586259	132712062	460	22	450
65	3'-5'	2748472	2748721	2749105	133874908	384	26	374
66	3'-5'	4514938	4515246	4515571	135641374	325	16	315
67	3'-5'	8086253	8086551	8086886	139212689	335	29	325
68	3'-5'	9385971	9386184	9386604	140512407	420	25	410
69	3'-5'	12591903	12592165	12592536	143718339	371	31	361
70	3'-5'	16807045	16807261	16807678	147933481	417	26	407
71	3'-5'	17554439	17554753	17555072	148680875	319	13	309
72	3'-5'	18034711	18034891	18035344	149161147	453	21	443
73	3'-5'	18226216	18226376	18226849	149352652	473	23	463
74	3'-5'	18941272	18941461	18941905	150067708	444	19	434
75	3'-5'	21300220	21300606	21300853	152426656	247	24	237
76	3'-5'	21707400	21707618	21708033	152833836	415	13	405
77	3'-5'	23084258	23084616	23084891	154210694	275	7	265
78	3'-5'	24412298	24412792	24412931	155538734	139	15	129
79	3'-5'	27338927	27339170	27339560	158465363	390	15	380

4			NT_039258.7 (1)					
	0	5'-3'	2256910	2256910	2257209	5256910	299	16	289
	1	5'-3'	3884978	3884978	3885414	6884978	436	10	426
	2	5'-3'	4754488	4754488	4754882	7754488	394	11	384
	3	5'-3'	14682726	14682726	14683083	17682726	357	22	347
	4	5'-3'	15760572	15760572	15760923	18760572	351	8	341
	5	5'-3'	17029298	17029298	17029728	20029298	430	9	420
	6	5'-3'	18967773	18967773	18968311	21967773	538	15	528
	7	5'-3'	19322567	19322567	19322948	22322567	381	6	371
	8	5'-3'	19767633	19767633	19767993	22767633	360	24	350
	9	5'-3'	20056938	20056938	20057345	23056938	407	24	397
	10	5'-3'	22327974	22327974	22328323	25327974	349	30	339
	11	5'-3'	23427886	23427886	23428276	26427886	390	26	380
	12	5'-3'	25358641	25358641	25358927	28358641	286	10	276
	13	3'-5'	3706981	3707207	3707614	6706981	407	11	397
	14	3'-5'	5855017	5855366	5855650	8855017	284	25	274
	15	3'-5'	8842376	8842487	8843009	11842376	522	29	512
	16	3'-5'	11679121	11679325	11679754	14679121	429	20	419
	17	3'-5'	14283304	14283742	14283937	17283304	195	17	185
	18	3'-5'	15453431	15453711	15454064	18453431	353	20	343
	19	3'-5'	20848070	20848320	20848703	23848070	383	26	373
	20	3'-5'	21556591	21556839	21557224	24556591	385	27	375
	21	3'-5'	22280936	22281101	22281569	25280936	468	9	458
	22	3'-5'	23378806	23379149	23379439	26378806	290	13	280
	23	3'-5'	23884716	23884818	23885349	26884716	531	30	521
	24	3'-5'	24178087	24178406	24178720	27178087	314	28	304
	25	3'-5'	24908281	24908633	24908914	27908281	281	6	271
	26	3'-5'	25821935	25822046	25822568	28821935	522	19	512
	27	3'-5'	26492443	26492706	26493076	29492443	370	18	360
	28	3'-5'	26915577	26915864	26916210	29915577	346	30	336
	29	3'-5'	27360534	27360812	27361167	30360534	355	31	345
	30	3'-5'	27542471	27542781	27543104	30542471	323	7	313
			NT_166289.1 (2)						
	31	5'-3'	1035044	1035044	1035498	32584745	454	10	444
	32	5'-3'	4596053	4596053	4596439	36145754	386	12	376
	33	5'-3'	7754482	7754482	7754797	39304183	315	27	305

34	5'-3'	7963809	7963809	7964115	39513510	306	30	296
35	3'-5'	4993614	4993916	4994247	36543315	331	17	321
		NT_109315.4 (3)						
36	5'-3'	2768309	2768309	2768667	44929234	358	21	348
37	5'-3'	5855311	5855311	5855761	48016236	450	16	440
38	5'-3'	8633740	8633740	8634121	50794665	381	18	371
39	5'-3'	9210525	9210525	9210820	51371450	295	20	285
40	5'-3'	10960810	10960810	10961332	53121735	522	6	512
41	5'-3'	12717035	12717035	12717366	54877960	331	11	321
42	5'-3'	15992849	15992849	15993266	58153774	417	12	407
43	3'-5'	8491359	8491445	8491992	50652284	547	24	537
44	3'-5'	9846699	9846973	9847332	52007624	359	27	349
45	3'-5'	10361131	10361214	10361764	52522056	550	33	540
46	3'-5'	13219433	13219629	13220066	55380358	437	26	427
47	3'-5'	14206685	14206836	14207318	56367610	482	22	472
		NT_039260.7 (4)						
48	5'-3'	1769055	1769055	1769353	62140440	298	24	288
49	5'-3'	1824076	1824076	1824460	62195461	384	22	374
50	5'-3'	1869903	1869903	1870414	62241288	511	11	501
51	5'-3'	3457760	3457760	3458268	63829145	508	11	498
52	5'-3'	6459320	6459320	6459667	66830705	347	8	337
53	5'-3'	9106470	9106470	9106954	69477855	484	13	474
54	5'-3'	9106509	9106509	9106954	69477894	445	24	435
55	5'-3'	11424440	11424440	11424843	71795825	403	24	393
56	5'-3'	12020658	12020658	12020976	72392043	318	12	308
57	5'-3'	13437970	13437970	13438495	73809355	525	27	515
58	5'-3'	13588582	13588582	13589056	73959967	474	7	464
59	5'-3'	14767424	14767424	14767847	75138809	423	31	413
60	5'-3'	14815188	14815188	14815509	75186573	321	19	311
61	5'-3'	17205660	17205660	17206137	77577045	477	9	467
62	5'-3'	17669392	17669392	17669729	78040777	337	30	327
63	5'-3'	19237439	19237439	19237931	79608824	492	13	482
64	5'-3'	21264144	21264144	21264600	81635529	456	11	446
65	5'-3'	21362091	21362091	21362570	81733476	479	24	469
66	5'-3'	24414002	24414002	24414312	84785387	310	19	300

67	5'-3'	24535500	24535500	24535747	84906885	247	29	237
68	3'-5'	3418829	3418882	3419462	63790214	580	10	570
69	3'-5'	3935513	3935642	3936146	64306898	504	27	494
70	3'-5'	4011750	4012054	4012383	64383135	329	9	319
71	3'-5'	5844308	5844564	5844941	66215693	377	34	367
72	3'-5'	8092692	8092940	8093325	68464077	385	27	375
73	3'-5'	12112238	12112442	12112871	72483623	429	8	419
74	3'-5'	12437002	12437283	12437635	72808387	352	21	342
75	3'-5'	12976648	12976902	12977281	73348033	379	17	369
76	3'-5'	15369632	15369875	15370265	75741017	390	26	380
77	3'-5'	16425660	16426000	16426293	76797045	293	27	283
78	3'-5'	16600902	16601063	16601535	76972287	472	7	462
79	3'-5'	17894202	17894476	17894835	78265587	359	11	349
80	3'-5'	17987263	17987754	17987896	78358648	142	8	132
81	3'-5'	18191502	18191736	18192135	78562887	399	18	389
82	3'-5'	18245905	18246126	18246538	78617290	412	10	402
83	3'-5'	18543087	18543383	18543720	78914472	337	6	327
84	3'-5'	19286841	19287064	19287474	79658226	410	7	400
85	3'-5'	19819114	19819351	19819747	80190499	396	24	386
86	3'-5'	22889298	22889445	22889931	83260683	486	12	476
87	3'-5'	23823959	23824032	23824592	84195344	560	32	550
88	3'-5'	24620225	24620566	24620858	84991610	292	14	282
89	3'-5'	24934721	24934933	24935354	85306106	421	34	411
90	3'-5'	25228179	25228458	25228812	85599564	354	9	344
91	3'-5'	28140924	28141209	28141557	88512309	348	25	338
		NT_039280.5 (5)						
92	5'-3'	2811347	2811347	2811747	91824055	400	8	390
93	5'-3'	2986103	2986103	2986464	91998811	361	20	351
94	5'-3'	3255187	3255187	3255564	92267895	377	31	367
95	5'-3'	5290293	5290293	5290638	94303001	345	31	335
96	5'-3'	6109010	6109010	6109198	95121718	188	9	178
97	5'-3'	6559599	6559599	6559919	95572307	320	15	310
98	5'-3'	9530919	9530919	9531215	98543627	296	16	286
99	5'-3'	9572904	9572904	9573233	98585612	329	29	319
100	3'-5'	256731	256924	257364	89269439	440	9	430
101	3'-5'	1489310	1489607	1489943	90502018	336	16	326

102	3'-5'	2744908	2744944	2745541	91757616	597	8	587
103	3'-5'	4306620	4306803	4307253	93319328	450	11	440
104	3'-5'	4478477	4478834	4479110	93491185	276	12	266
		NT_039264.6 (6)						
105	5'-3'	1832269	1832269	1832644	101381070	375	12	365
106	5'-3'	2328131	2328131	2328534	101876932	403	19	393
107	5'-3'	7011333	7011333	7011846	106560134	513	11	503
108	5'-3'	9873848	9873848	9874239	109422649	391	16	381
109	5'-3'	10672098	10672098	10672587	110220899	489	26	479
110	5'-3'	14627046	14627046	14627458	114175847	412	28	402
111	5'-3'	17333156	17333156	17333297	116881957	141	13	131
112	3'-5'	1662796	1663098	1663429	101211597	331	9	321
113	3'-5'	3889983	3890259	3890616	103438784	357	17	347
114	3'-5'	5327137	5327469	5327770	104875938	301	20	291
115	3'-5'	7844270	7844507	7844903	107393071	396	33	386
116	3'-5'	8966191	8966410	8966824	108514992	414	15	404
117	3'-5'	9607988	9608249	9608621	109156789	372	17	362
118	3'-5'	10810792	10811221	10811425	110359593	204	29	194
119	3'-5'	11275029	11275457	11275662	110823830	205	9	195
120	3'-5'	11744151	11744385	11744784	111292952	399	16	389
121	3'-5'	12855907	12856159	12856540	112404708	381	9	371
122	3'-5'	14838867	14839137	14839500	114387668	363	9	353
123	3'-5'	21753253	21753699	21753886	121302054	187	22	177
		NT_109316.2 (7)						
124	3'-5'	280599	280902	281232	121779355	330	15	320
		NT_109317.2 (8)						
125	5'-3'	650116	650116	650643	122735021	527	9	517
126	5'-3'	4188616	4188616	4188864	126273521	248	22	238
127	5'-3'	7179245	7179245	7179667	129264150	422	15	412
128	3'-5'	3017289	3017336	3017922	125102194	586	15	576
129	3'-5'	4712916	4713146	4713549	126797821	403	27	393
130	3'-5'	4927943	4928437	4928576	127012848	139	13	129
		NT_039267.7 (9)						

	131	5'-3'	1477506	1477506	1477847	131597772	341	27	331
	132	5'-3'	4208093	4208093	4208535	134328359	442	29	432
			NT_162293.1 (10)						
	133	5'-3'	782043	782043	782282	142859844	239	27	229
	134	3'-5'	120475	120731	121108	142198276	377	26	367
			NT_039268.5 (11)						
	135	5'-3'	2449624	2449624	2450013	147989502	389	19	379
5			NT_039299.7 (1)						
	0	5'-3'	4256606	4256606	4257081	7256606	475	9	465
	1	5'-3'	4648394	4648394	4648756	7648394	362	10	352
	2	5'-3'	6221649	6221649	6222084	9221649	435	20	425
	3	5'-3'	6301822	6301822	6302190	9301822	368	20	358
	4	5'-3'	9619663	9619663	9619923	12619663	260	20	250
	5	5'-3'	9782389	9782389	9782640	12782389	251	12	241
	6	5'-3'	11705456	11705456	11705832	14705456	376	28	366
	7	3'-5'	88118	88370	88751	3088118	381	14	371
	8	3'-5'	3031157	3031574	3031790	6031157	216	7	206
	9	3'-5'	3055254	3055513	3055887	6055254	374	31	364
	10	3'-5'	3275416	3275463	3276049	6275416	586	29	576
	11	3'-5'	6622988	6623090	6623621	9622988	531	18	521
	12	3'-5'	7109645	7109746	7110278	10109645	532	22	522
	13	3'-5'	9819538	9819744	9820171	12819538	427	30	417
	14	3'-5'	11200804	11201021	11201437	14200804	416	20	406
	15	3'-5'	11722143	11722402	11722776	14722143	374	11	364
			NT_165760.2 (2)						
	16	5'-3'	798098	798098	798517	15910764	419	29	409
	17	5'-3'	4425886	4425886	4426276	19538552	390	29	380
	18	5'-3'	4667912	4667912	4668298	19780578	386	23	376
	19	5'-3'	6007740	6007740	6008127	21120406	387	7	377
	20	5'-3'	6313417	6313417	6313809	21426083	392	23	382
	21	5'-3'	7798378	7798378	7798656	22911044	278	29	268
	22	3'-5'	443994	444212	444627	15556660	415	7	405
	23	3'-5'	454161	454423	454794	15566827	371	18	361

24	3'-5'	902471	902769	903104	16015137	335	8	325
25	3'-5'	3702825	3703094	3703458	18815491	364	13	354
26	3'-5'	6314291	6314619	6314924	21426957	305	21	295
		NT_166300.1 (4)						
27	3'-5'	1394307	1394592	1394940	32036303	348	29	338
28	3'-5'	2352799	2352996	2353432	32994795	436	22	426
		NT_039302.7 (5)						
29	5'-3'	3897479	3897479	3897895	36963847	416	26	406
		NT_039305.7 (6)						
30	5'-3'	4067452	4067452	4067780	41119347	328	11	318
31	5'-3'	4216285	4216285	4216782	41268180	497	24	487
32	5'-3'	7084414	7084414	7084795	44136309	381	31	371
33	5'-3'	8012533	8012533	8012997	45064428	464	7	454
34	5'-3'	11456685	11456685	11457019	48508580	334	26	324
35	5'-3'	12107769	12107769	12108144	49159664	375	30	365
36	5'-3'	18025269	18025269	18025668	55077164	399	29	389
37	5'-3'	20162996	20162996	20163392	57214891	396	23	386
38	5'-3'	21273846	21273846	21274097	58325741	251	13	241
39	5'-3'	22939143	22939143	22939481	59991038	338	16	328
40	5'-3'	23336643	23336643	23336892	60388538	249	9	239
41	5'-3'	25920185	25920185	25920646	62972080	461	31	451
42	5'-3'	27131499	27131499	27131900	64183394	401	17	391
43	5'-3'	27496362	27496362	27496736	64548257	374	20	364
44	5'-3'	27496412	27496412	27496736	64548307	324	20	314
45	5'-3'	28010211	28010211	28010626	65062106	415	22	405
46	5'-3'	32323264	32323264	32323643	69375159	379	27	369
47	5'-3'	34738180	34738180	34738553	71790075	373	10	363
48	5'-3'	35509226	35509226	35509675	72561121	449	6	439
49	5'-3'	35818830	35818830	35819454	72870725	624	8	614
50	3'-5'	4495830	4496070	4496463	41547725	393	32	383
51	3'-5'	5172961	5173263	5173594	42224856	331	27	321
52	3'-5'	11716651	11717000	11717284	48768546	284	16	274
53	3'-5'	12722535	12722773	12723168	49774430	395	11	385
54	3'-5'	16341878	16342160	16342511	53393773	351	6	341
55	3'-5'	17745591	17745834	17746224	54797486	390	23	380
56	3'-5'	21470678	21470900	21471311	58522573	411	6	401

57	3'-5'	25789224	25789475	25789857	62841119	382	20	372
58	3'-5'	26098248	26098365	26098881	63150143	516	17	506
59	3'-5'	26901317	26901577	26901950	63953212	373	34	363
60	3'-5'	29264936	29265311	29265569	66316831	258	31	248
61	3'-5'	32550692	32550800	32551325	69602587	525	34	515
62	3'-5'	32684727	32685040	32685360	69736622	320	8	310
63	3'-5'	33193213	33193324	33193846	70245108	522	31	512
64	3'-5'	34856346	34856580	34856979	71908241	399	16	389
		NT_039306.7 (7)						
65	3'-5'	347991	348099	348624	74731248	525	12	515
		NT_109320.4 (8)						
66	5'-3'	141065	141065	141442	76668944	377	23	367
67	5'-3'	2003159	2003159	2003642	78531038	483	15	473
68	5'-3'	3529974	3529974	3530381	80057853	407	17	397
69	5'-3'	3922883	3922883	3923167	80450762	284	31	274
70	5'-3'	4046354	4046354	4046806	80574233	452	24	442
71	5'-3'	4343074	4343074	4343449	80870953	375	6	365
72	5'-3'	6749566	6749566	6749963	83277445	397	8	387
73	3'-5'	2068161	2068332	2068794	78596040	462	21	452
74	3'-5'	2718513	2718693	2719146	79246392	453	22	443
75	3'-5'	3085670	3086001	3086303	79613549	302	15	292
76	3'-5'	4658791	4659066	4659424	81186670	358	31	348
77	3'-5'	7510819	7511080	7511452	84038698	372	7	362
		NT_078458.6 (9)						
78	5'-3'	3133425	3133425	3133655	117119419	230	11	220
79	5'-3'	10384267	10384267	10384607	124370261	340	18	330
80	3'-5'	2846146	2846373	2846779	116832140	406	17	396
81	3'-5'	5162017	5162286	5162650	119148011	364	34	354
		NT_039313.7 (10)						
82	3'-5'	3456269	3456535	3456902	128646608	367	34	357
		NT_039314.7 (11)						
83	5'-3'	1037568	1037568	1037956	131358154	388	22	378

	84	5'-3'	3259002	3259002	3259599	133579588	597	16	587
	85	5'-3'	4127077	4127077	4127503	134447663	426	23	416
	86	3'-5'	774127	774354	774760	131094713	406	25	396
	87	3'-5'	1927156	1927414	1927789	132247742	375	16	365
	88	3'-5'	2613234	2613469	2613867	132933820	398	30	388
	89	3'-5'	3556554	3556826	3557187	133877140	361	33	351
			NT_039316.7 (13)						
	90	3'-5'	2017478	2017959	2018111	141176358	152	26	142
			NT_081055.6 (15)						
	91	5'-3'	1083300	1083300	1083920	144495051	620	8	610
	92	3'-5'	27092	27511	27725	143438843	214	29	204
			NT_039324.7 (17)						
	93	5'-3'	1427686	1427686	1428109	147900661	423	25	413
	94	5'-3'	1458552	1458552	1458873	147931527	321	22	311
	95	3'-5'	1867701	1867957	1868334	148340676	377	6	367
	96	3'-5'	3461081	3461327	3461714	149934056	387	34	377
			NT_039325.7 (18)						
	97	5'-3'	1000613	1000613	1000997	151115811	384	19	374
	98	3'-5'	1299615	1299803	1300248	151414813	445	30	435
6			NT_039340.7 (1)						
	0	5'-3'	1347104	1347104	1347559	4347104	455	27	445
	1	5'-3'	1365691	1365691	1366017	4365691	326	11	316
	2	5'-3'	3545508	3545508	3545887	6545508	379	17	369
	3	5'-3'	7064069	7064069	7064402	10064069	333	22	323
	4	5'-3'	7555589	7555589	7555975	10555589	386	10	376
	5	5'-3'	8363995	8363995	8364348	11363995	353	17	343
	6	5'-3'	11435061	11435061	11435610	14435061	549	31	539
	7	5'-3'	11461511	11461511	11461879	14461511	368	28	358
	8	5'-3'	12660909	12660909	12661205	15660909	296	27	286
	9	5'-3'	14422612	14422612	14422923	17422612	311	17	301
	10	5'-3'	17213196	17213196	17213567	20213196	371	21	361
	11	5'-3'	19513270	19513270	19513757	22513270	487	9	477

12	5'-3'	21103218	21103218	21103584	24103218	366	27	356
13	5'-3'	22366477	22366477	22366903	25366477	426	14	416
14	5'-3'	23246539	23246539	23246940	26246539	401	22	391
15	5'-3'	23270369	23270369	23270724	26270369	355	28	345
16	5'-3'	23364355	23364355	23364739	26364355	384	21	374
17	5'-3'	24423429	24423429	24423821	27423429	392	30	382
18	5'-3'	26292277	26292277	26292664	29292277	387	26	377
19	3'-5'	4826628	4826854	4827261	7826628	407	31	397
20	3'-5'	6227329	6227551	6227962	9227329	411	22	401
21	3'-5'	6653118	6653368	6653751	9653118	383	18	373
22	3'-5'	7134407	7134757	7135040	10134407	283	9	273
23	3'-5'	10949105	10949533	10949738	13949105	205	13	195
24	3'-5'	13041723	13042034	13042356	16041723	322	7	312
25	3'-5'	14705603	14705865	14706236	17705603	371	12	361
26	3'-5'	16191334	16191463	16191967	19191334	504	6	494
27	3'-5'	18107654	18107852	18108287	21107654	435	31	425
28	3'-5'	19221053	19221214	19221686	22221053	472	14	462
29	3'-5'	24158956	24159249	24159589	27158956	340	22	330
30	3'-5'	25035853	25036148	25036486	28035853	338	24	328
31	3'-5'	25151464	25151594	25152097	28151464	503	19	493
32	3'-5'	27084241	27084436	27084874	30084241	438	24	428
		NT_039341.7 (2)						
33	5'-3'	453218	453218	453683	31206029	465	30	455
34	5'-3'	1946692	1946692	1947304	32699503	612	7	602
35	5'-3'	2364322	2364322	2364531	33117133	209	13	199
36	5'-3'	3748164	3748164	3748485	34500975	321	16	311
37	5'-3'	6143216	6143216	6143803	36896027	587	8	577
38	5'-3'	8085870	8085870	8086257	38838681	387	15	377
39	5'-3'	10774896	10774896	10775395	41527707	499	31	489
40	5'-3'	11565533	11565533	11565863	42318344	330	17	320
41	5'-3'	13935069	13935069	13935394	44687880	325	20	315
42	5'-3'	15571352	15571352	15571711	46324163	359	21	349
43	3'-5'	683523	683972	684156	31436334	184	26	174
44	3'-5'	3940925	3941152	3941558	34693736	406	12	396
45	3'-5'	7973093	7973466	7973726	38725904	260	13	250
46	3'-5'	8407819	8408195	8408452	39160630	257	20	247

47	3'-5'	8938654	8938914	8939287	39691465	373	34	363
48	3'-5'	9145270	9145471	9145903	39898081	432	26	422
49	3'-5'	10212621	10212868	10213254	40965432	386	25	376
50	3'-5'	11136089	11136304	11136722	41888900	418	15	408
51	3'-5'	12808679	12808940	12809312	43561490	372	31	362
		NT_039353.7 (3)						
52	5'-3'	2539423	2539423	2539791	50226461	368	14	358
53	5'-3'	3267975	3267975	3268444	50955013	469	17	459
54	5'-3'	4118471	4118471	4119062	51805509	591	14	581
55	5'-3'	4255629	4255629	4255873	51942667	244	26	234
56	5'-3'	4939324	4939324	4939762	52626362	438	30	428
57	5'-3'	8636914	8636914	8637191	56323952	277	7	267
58	5'-3'	9915493	9915493	9915902	57602531	409	13	399
59	5'-3'	13756207	13756207	13756656	61443245	449	26	439
60	5'-3'	14959707	14959707	14960040	62646745	333	29	323
61	5'-3'	22559184	22559184	22559583	70246222	399	30	389
62	5'-3'	24762350	24762350	24762756	72449388	406	19	396
63	5'-3'	29260196	29260196	29260644	76947234	448	11	438
64	5'-3'	29400439	29400439	29400861	77087477	422	25	412
65	5'-3'	37538364	37538364	37538517	85225402	153	19	143
66	5'-3'	44631506	44631506	44631715	92318544	209	11	199
67	5'-3'	47299829	47299829	47300332	94986867	503	27	493
68	5'-3'	48760533	48760533	48760905	96447571	372	14	362
69	5'-3'	48931038	48931038	48931288	96618076	250	9	240
70	5'-3'	51534325	51534325	51534843	99221363	518	30	508
71	5'-3'	53997891	53997891	53998188	101684929	297	27	287
72	5'-3'	57872032	57872032	57872297	105559070	265	21	255
73	5'-3'	58754899	58754899	58755295	106441937	396	9	386
74	5'-3'	59028401	59028401	59028815	106715439	414	27	404
75	5'-3'	60803170	60803170	60803319	108490208	149	13	139
76	5'-3'	62703332	62703332	62703697	110390370	365	9	355
77	5'-3'	62826761	62826761	62826911	110513799	150	16	140
78	5'-3'	68345955	68345955	68346368	116032993	413	22	403
79	5'-3'	72325203	72325203	72325459	120012241	256	23	246
80	5'-3'	72891211	72891211	72891652	120578249	441	23	431
81	5'-3'	73400636	73400636	73401012	121087674	376	16	366

82	3'-5'	941379	941850	942012	48628417	162	18	152
83	3'-5'	4148052	4148265	4148685	51835090	420	32	410
84	3'-5'	6866657	6866833	6867290	54553695	457	30	447
85	3'-5'	9171121	9171300	9171754	56858159	454	7	444
86	3'-5'	11557411	11557665	11558044	59244449	379	8	369
87	3'-5'	12299331	12299533	12299964	59986369	431	11	421
88	3'-5'	13039410	13039666	13040043	60726448	377	9	367
89	3'-5'	14037327	14037744	14037960	61724365	216	32	206
90	3'-5'	19698901	19699152	19699534	67385939	382	9	372
91	3'-5'	19804846	19805023	19805479	67491884	456	12	446
92	3'-5'	22151094	22151350	22151727	69838132	377	32	367
93	3'-5'	25546980	25547119	25547613	73234018	494	21	484
94	3'-5'	26370701	26370883	26371334	74057739	451	17	441
95	3'-5'	28537566	28537668	28538199	76224604	531	32	521
96	3'-5'	30296483	30296764	30297116	77983521	352	6	342
97	3'-5'	30777144	30777395	30777777	78464182	382	29	372
98	3'-5'	32329120	32329428	32329753	80016158	325	29	315
99	3'-5'	32963551	32963905	32964184	80650589	279	27	269
100	3'-5'	33333772	33333904	33334405	81020810	501	19	491
101	3'-5'	33426598	33426753	33427231	81113636	478	12	468
102	3'-5'	36246599	36246971	36247232	83933637	261	11	251
103	3'-5'	37158665	37158927	37159298	84845703	371	15	361
104	3'-5'	42037991	42038195	42038624	89725029	429	11	419
105	3'-5'	47423597	47423796	47424230	95110635	434	23	424
106	3'-5'	48521326	48521613	48521959	96208364	346	24	336
107	3'-5'	49967472	49967728	49968105	97654510	377	22	367
108	3'-5'	50317597	50317787	50318230	98004635	443	24	433
109	3'-5'	51017659	51017993	51018292	98704697	299	29	289
110	3'-5'	51257823	51257982	51258456	98944861	474	17	464
111	3'-5'	52537026	52537326	52537659	100224064	333	30	323
112	3'-5'	54977355	54977761	54977988	102664393	227	6	217
113	3'-5'	55127910	55127968	55128543	102814948	575	26	565
114	3'-5'	55373204	55373417	55373837	103060242	420	30	410
115	3'-5'	61670979	61671170	61671612	109358017	442	16	432
116	3'-5'	63058605	63058922	63059238	110745643	316	14	306
117	3'-5'	65576796	65576869	65577429	113263834	560	8	550
118	3'-5'	65601390	65601573	65602023	113288428	450	27	440

	119	3'-5'	67621552	67621836	67622185	115308590	349	26	339
	120	3'-5'	67632940	67633242	67633573	115319978	331	10	321
	121	3'-5'	67685383	67685463	67686016	115372421	553	14	543
	122	3'-5'	68368124	68368492	68368757	116055162	265	18	255
	123	3'-5'	71698931	71699409	71699564	119385969	155	7	145
	124	3'-5'	74914462	74914707	74915095	122601500	388	28	378
	125	3'-5'	82672416	82672580	82673049	130359454	469	32	459
	126	3'-5'	83971945	83972230	83972578	131658983	348	26	338
	127	3'-5'	84856225	84856467	84856858	132543263	391	19	381
	128	3'-5'	85125709	85126132	85126342	132812747	210	26	200
	129	3'-5'	87540714	87541013	87541347	135227752	334	9	324
			NT_039360.7 (4)						
	130	5'-3'	1884596	1884596	1884953	141546028	357	6	347
	131	5'-3'	3312882	3312882	3313081	142974314	199	14	189
	132	3'-5'	1264376	1264648	1265009	140925808	361	14	351
	133	3'-5'	1672519	1672650	1673152	141333951	502	14	492
	134	3'-5'	5126861	5126993	5127494	144788293	501	19	491
			Nt_039361.6 (5)						
	135	5'-3'	981793	981793	982161	147000322	368	8	358
	136	5'-3'	2827905	2827905	2828206	148846434	301	25	291
	137	3'-5'	520196	520452	520829	146538725	377	34	367
7			NT_039413.7 (1)						
	0	5'-3'	124148	124148	124489	3124148	341	14	331
	1	5'-3'	3552464	3552464	3552888	6552464	424	29	414
	2	5'-3'	4065305	4065305	4065674	7065305	369	9	359
	3	5'-3'	5258601	5258601	5258991	8258601	390	11	380
	4	5'-3'	5570852	5570852	5571239	8570852	387	11	377
	5	5'-3'	5758557	5758557	5758944	8758557	387	11	377
	6	5'-3'	5960473	5960473	5960861	8960473	388	11	378
	7	5'-3'	6058179	6058179	6058569	9058179	390	11	380
	8	5'-3'	6213863	6213863	6214251	9213863	388	11	378
	9	5'-3'	6257906	6257906	6258293	9257906	387	11	377
	10	5'-3'	6364502	6364502	6364892	9364502	390	11	380
	11	5'-3'	6644408	6644408	6644798	9644408	390	11	380

12	5'-3'	6716000	6716000	6716387	9716000	387	11	377
13	5'-3'	6933134	6933134	6933522	9933134	388	11	378
14	5'-3'	6977237	6977237	6977624	9977237	387	11	377
15	5'-3'	7083850	7083850	7084240	10083850	390	11	380
16	5'-3'	7104553	7104553	7104943	10104553	390	11	380
17	5'-3'	9452470	9452470	9452855	12452470	385	22	375
18	5'-3'	17138548	17138548	17138808	20138548	260	17	250
19	5'-3'	17157627	17157627	17157880	20157627	253	22	243
20	5'-3'	23292602	23292602	23292963	26292602	361	22	351
21	5'-3'	29265322	29265322	29265922	32265322	600	13	590
22	5'-3'	32177667	32177667	32178062	35177667	395	20	385
23	5'-3'	33985417	33985417	33985862	36985417	445	30	435
24	3'-5'	1175805	1176161	1176438	4175805	277	12	267
25	3'-5'	1551693	1552123	1552326	4551693	203	32	193
26	3'-5'	2478045	2478290	2478678	5478045	388	11	378
27	3'-5'	4139387	4139617	4140020	7139387	403	12	393
28	3'-5'	4276756	4276999	4277389	7276756	390	11	380
29	3'-5'	4426056	4426301	4426689	7426056	388	11	378
30	3'-5'	4539016	4539259	4539649	7539016	390	11	380
31	3'-5'	4635348	4635593	4635981	7635348	388	11	378
32	3'-5'	4840959	4841205	4841592	7840959	387	11	377
33	3'-5'	5074941	5075187	5075574	8074941	387	11	377
34	3'-5'	7273138	7273384	7273771	10273138	387	11	377
35	3'-5'	7413694	7413937	7414327	10413694	390	11	380
36	3'-5'	7526833	7527079	7527466	10526833	387	11	377
37	3'-5'	7570853	7571098	7571486	10570853	388	11	378
38	3'-5'	7684412	7684654	7685045	10684412	391	11	381
39	3'-5'	7911494	7911742	7912127	10911494	385	25	375
40	3'-5'	10043378	10043673	10044011	13043378	338	20	328
41	3'-5'	10927170	10927556	10927803	13927170	247	16	237
42	3'-5'	21452258	21452508	21452891	24452258	383	10	373
43	3'-5'	27066980	27067461	27067613	30066980	152	7	142
44	3'-5'	29430354	29430507	29430987	32430354	480	6	470
45	3'-5'	31240126	31240351	31240759	34240126	408	27	398
46	3'-5'	35816985	35817008	35817618	38816985	610	22	600

NT_039424.7 (2)

47	5'-3'	4913713	4913713	4914274	51269907	561	25	551
48	5'-3'	8465502	8465502	8465842	54821696	340	26	330
49	5'-3'	8511565	8511565	8511905	54867759	340	26	330
50	5'-3'	8867418	8867418	8867617	55223612	199	31	189
51	5'-3'	9478770	9478770	9479116	55834964	346	17	336
52	5'-3'	14749689	14749689	14750053	61105883	364	29	354
53	5'-3'	15962634	15962634	15962995	62318828	361	24	351
54	5'-3'	18213867	18213867	18214277	64570061	410	12	400
55	3'-5'	8708530	8708824	8709163	55064724	339	31	329
56	3'-5'	12995777	12996162	12996410	59351971	248	7	238
57	3'-5'	13242320	13242579	13242953	59598514	374	31	364
58	3'-5'	15483906	15484246	15484539	61840100	293	25	283
59	3'-5'	17459422	17459688	17460055	63815616	367	11	357
60	3'-5'	18985123	18985531	18985756	65341317	225	22	215
61	3'-5'	19260928	19261124	19261561	65617122	437	14	427
62	3'-5'	20183450	20183702	20184083	66539644	381	23	371
		NT_039428.7 (3)						
63	5'-3'	2950767	2950767	2951233	69952972	466	21	456
64	5'-3'	8923599	8923599	8924109	75925804	510	9	500
65	5'-3'	9831923	9831923	9832307	76834128	384	23	374
66	5'-3'	9896812	9896812	9897286	76899017	474	22	464
67	5'-3'	10295237	10295237	10295547	77297442	310	13	300
68	5'-3'	12229544	12229544	12230059	79231749	515	27	505
69	5'-3'	12315351	12315351	12315711	79317556	360	16	350
70	5'-3'	14549559	14549559	14549831	81551764	272	18	262
71	5'-3'	17045877	17045877	17046092	84048082	215	27	205
72	5'-3'	18307225	18307225	18307607	85309430	382	29	372
73	5'-3'	21318709	21318709	21319210	88320914	501	15	491
74	5'-3'	21532638	21532638	21533017	88534843	379	6	369
75	5'-3'	21562396	21562396	21562814	88564601	418	12	408
76	3'-5'	159097	159307	159730	67161302	423	20	413
77	3'-5'	186457	186740	187090	67188662	350	16	340
78	3'-5'	441914	442148	442547	67444119	399	8	389
79	3'-5'	916924	917175	917557	67919129	382	8	372
80	3'-5'	2149704	2149958	2150337	69151909	379	27	369
81	3'-5'	2920890	2921156	2921523	69923095	367	11	357

82	3'-5'	2971487	2971807	2972120	69973692	313	16	303
83	3'-5'	11108614	11108840	11109247	78110819	407	21	397
84	3'-5'	12695648	12695909	12696281	79697853	372	25	362
85	3'-5'	12909514	12909827	12910147	79911719	320	28	310
86	3'-5'	16514694	16514870	16515327	83516899	457	27	447
87	3'-5'	16858757	16858970	16859390	83860962	420	9	410
88	3'-5'	17267567	17267853	17268200	84269772	347	32	337
89	3'-5'	17353668	17353756	17354301	84355873	545	32	535
90	3'-5'	21717797	21717952	21718430	88720002	478	16	468
		NT_039433.7 (4)						
91	5'-3'	826098	826098	826561	90225931	463	11	453
92	5'-3'	1874946	1874946	1875336	91274779	390	9	380
93	5'-3'	3909868	3909868	3910394	93309701	526	10	516
94	5'-3'	4545153	4545153	4545528	93944986	375	23	365
95	5'-3'	5631456	5631456	5631932	95031289	476	19	466
96	5'-3'	10249776	10249776	10249959	99649609	183	7	173
97	5'-3'	11885567	11885567	11885907	101285400	340	26	330
98	5'-3'	13475631	13475631	13476055	102875464	424	28	414
99	5'-3'	14044063	14044063	14044383	103443896	320	30	310
100	5'-3'	16087566	16087566	16087786	105487399	220	9	210
101	5'-3'	21258411	21258411	21258892	110658244	481	25	471
102	5'-3'	24338505	24338505	24338942	113738338	437	30	427
103	5'-3'	24642977	24642977	24643411	114042810	434	27	424
104	5'-3'	31515825	31515825	31516140	120915658	315	9	305
105	5'-3'	32742669	32742669	32743056	122142502	387	11	377
106	5'-3'	33133858	33133858	33134409	122533691	551	25	541
107	5'-3'	35225765	35225765	35226102	124625598	337	16	327
108	5'-3'	48059515	48059515	48059934	137459348	419	18	409
109	5'-3'	49811510	49811510	49811764	139211343	254	14	244
110	3'-5'	104770	104994	105403	89504603	409	18	399
111	3'-5'	5062053	5062270	5062686	94461886	416	21	406
112	3'-5'	7129032	7129170	7129665	96528865	495	15	485
113	3'-5'	8847743	8848008	8848376	98247576	368	9	358
114	3'-5'	9384715	9385130	9385348	98784548	218	7	208
115	3'-5'	10447775	10447861	10448408	99847608	547	20	537
116	3'-5'	15748330	15748598	15748963	105148163	365	19	355

	117	3'-5'	16992070	16992450	16992703	106391903	253	29	243
	118	3'-5'	19639470	19639877	19640103	109039303	226	27	216
	119	3'-5'	21549931	21550202	21550564	110949764	362	26	352
	120	3'-5'	21743570	21743821	21744203	111143403	382	34	372
	121	3'-5'	25903223	25903497	25903856	115303056	359	24	349
	122	3'-5'	25999505	25999771	26000138	115399338	367	24	357
	123	3'-5'	27744119	27744400	27744752	117143952	352	22	342
	124	3'-5'	29134152	29134403	29134785	118533985	382	15	372
	125	3'-5'	34399061	34399412	34399694	123798894	282	32	272
	126	3'-5'	37164634	37165071	37165267	126564467	196	24	186
	127	3'-5'	38199587	38199901	38200220	127599420	319	18	309
	128	3'-5'	38342552	38342680	38343185	127742385	505	22	495
	129	3'-5'	39062210	39062475	39062843	128462043	368	22	358
	130	3'-5'	40460628	40461051	40461261	129860461	210	12	200
	131	3'-5'	41015301	41015519	41015934	130415134	415	13	405
	132	3'-5'	41105115	41105364	41105748	130504948	384	19	374
	133	3'-5'	43105348	43105506	43105981	132505181	475	12	465
	134	3'-5'	51136563	51136982	51137196	140536396	214	11	204
	135	3'-5'	54945099	54945351	54945732	144344932	381	8	371
	136	3'-5'	55624230	55624539	55624863	145024063	324	15	314
	137	3'-5'	56649905	56650131	56650538	146049738	407	20	397
			NT_166306.1 (5)						
	138	3'-5'	2254468	2254535	2255101	148502626	566	9	556
			NT_039437.7 (6)						
	139	3'-5'	2369260	2369454	2369893	151299776	439	32	429
	140	3'-5'	2536766	2537036	2537399	151467282	363	23	353
8			NT_039455.7 (1)						
	0	5'-3'	798673	798673	799022	3798673	349	16	339
	1	5'-3'	3488154	3488154	3488489	6488154	335	31	325
	2	5'-3'	4221184	4221184	4221427	7221184	243	29	233
	3	5'-3'	4499670	4499670	4500023	7499670	353	14	343
	4	5'-3'	5695817	5695817	5696265	8695817	448	23	438
	5	5'-3'	7143803	7143803	7144204	10143803	401	25	391
	6	5'-3'	9618341	9618341	9618749	12618341	408	28	398

7	5'-3'	12480602	12480602	12481053	15480602	451	21	441
8	5'-3'	17028053	17028053	17028414	20028053	361	16	351
9	3'-5'	3092120	3092350	3092753	6092120	403	10	393
10	3'-5'	3217784	3218057	3218417	6217784	360	7	350
11	3'-5'	5003037	5003329	5003670	8003037	341	21	331
12	3'-5'	5724305	5724783	5724938	8724305	155	18	145
13	3'-5'	8837920	8837999	8838553	11837920	554	26	544
14	3'-5'	13285426	13285903	13286059	16285426	156	29	146
15	3'-5'	14025707	14026008	14026340	17025707	332	34	322
16	3'-5'	14931340	14931732	14931973	17931340	241	32	231
17	3'-5'	15401736	15401909	15402369	18401736	460	17	450
18	3'-5'	15580998	15581243	15581631	18580998	388	10	378
		NT_039457.7 (2)						
19	5'-3'	1379401	1379401	1379613	23418114	212	22	202
20	5'-3'	1678784	1678784	1679057	23717497	273	18	263
21	5'-3'	2767148	2767148	2767461	24805861	313	6	303
22	5'-3'	9132535	9132535	9132815	31171248	280	20	270
23	5'-3'	11502550	11502550	11502769	33541263	219	30	209
24	5'-3'	12954631	12954631	12954985	34993344	354	24	344
25	5'-3'	14235655	14235655	14236154	36274368	499	24	489
26	5'-3'	14473763	14473763	14474200	36512476	437	19	427
27	5'-3'	16020556	16020556	16021116	38059269	560	22	550
28	5'-3'	17323264	17323264	17323635	39361977	371	21	361
29	3'-5'	1529679	1529941	1530312	23568392	371	30	361
30	3'-5'	3422843	3423022	3423476	25461556	454	33	444
31	3'-5'	4708841	4709165	4709474	26747554	309	12	299
32	3'-5'	9511528	9511875	9512161	31550241	286	29	276
33	3'-5'	10062333	10062581	10062966	32101046	385	9	375
		NT_039460.7 (3)						
34	5'-3'	3307739	3307739	3308091	43545883	352	10	342
35	5'-3'	3754462	3754462	3754894	43992606	432	13	422
36	5'-3'	3810775	3810775	3811260	44048919	485	10	475
37	5'-3'	5166815	5166815	5167117	45404959	302	21	292
38	5'-3'	6219261	6219261	6219751	46457405	490	7	480
39	5'-3'	6251144	6251144	6251603	46489288	459	8	449

40	5'-3'	6470534	6470534	6470998	46708678	464	17	454
41	5'-3'	11237562	11237562	11237934	51475706	372	18	362
42	5'-3'	12643360	12643360	12643749	52881504	389	31	379
43	5'-3'	13495700	13495700	13495994	53733844	294	27	284
44	5'-3'	14212962	14212962	14213347	54451106	385	19	375
45	5'-3'	14885072	14885072	14885373	55123216	301	14	291
46	3'-5'	3060267	3060333	3060900	43298411	567	14	557
47	3'-5'	3739177	3739508	3739810	43977321	302	22	292
48	3'-5'	5305546	5306017	5306179	45543690	162	9	152
49	3'-5'	8003913	8004169	8004546	48242057	377	13	367
50	3'-5'	8444784	8444966	8445417	48682928	451	28	441
51	3'-5'	9361781	9362061	9362414	49599925	353	11	343
52	3'-5'	9709144	9709393	9709777	49947288	384	34	374
53	3'-5'	10003730	10003834	10004363	50241874	529	14	519
54	3'-5'	10895024	10895368	10895657	51133168	289	10	279
55	3'-5'	12159258	12159516	12159891	52397402	375	11	365
56	3'-5'	14095715	14095907	14096348	54333859	441	8	431
		NT_039461.7 (4)						
57	5'-3'	1981979	1981979	1982312	58361044	333	22	323
58	5'-3'	2616368	2616368	2616717	58995433	349	27	339
59	5'-3'	4228766	4228766	4229096	60607831	330	28	320
60	5'-3'	6928189	6928189	6928511	63307254	322	10	312
61	5'-3'	7231056	7231056	7231418	63610121	362	23	352
62	5'-3'	8076563	8076563	8076902	64455628	339	23	329
63	5'-3'	9281593	9281593	9281951	65660658	358	20	348
64	3'-5'	1186905	1187249	1187538	57565970	289	7	279
65	3'-5'	2010531	2010633	2011164	58389596	531	27	521
66	3'-5'	6652235	6652466	6652868	63031300	402	28	392
67	3'-5'	8204061	8204474	8204694	64583126	220	33	210
		NT_039462.7 (5)						
68	5'-3'	1601573	1601573	1601933	67400091	360	23	350
69	5'-3'	4358006	4358006	4358299	70156524	293	10	283
70	5'-3'	5610776	5610776	5611005	71409294	229	19	219
71	3'-5'	1867018	1867224	1867651	67665536	427	22	417
72	3'-5'	5240679	5240943	5241312	71039197	369	8	359

73	3'-5'	7351104	7351341	7351737	73149622	396	23	386
		NT_078575.6 (6)						
74	5'-3'	1830597	1830597	1831109	75467243	512	6	502
75	5'-3'	3888709	3888709	3889289	77525355	580	25	570
76	5'-3'	5717011	5717011	5717375	79353657	364	8	354
77	5'-3'	6412410	6412410	6412762	80049056	352	29	342
78	5'-3'	6767090	6767090	6767579	80403736	489	19	479
79	5'-3'	7657421	7657421	7657704	81294067	283	14	273
80	5'-3'	8579382	8579382	8579812	82216028	430	12	420
81	5'-3'	10265489	10265489	10265863	83902135	374	16	364
82	5'-3'	17192424	17192424	17192799	90829070	375	17	365
83	5'-3'	20009777	20009777	20010097	93646423	320	30	310
84	5'-3'	21577391	21577391	21577787	95214037	396	7	386
85	5'-3'	22643634	22643634	22643848	96280280	214	29	204
86	5'-3'	23600892	23600892	23601213	97237538	321	7	311
87	5'-3'	24278754	24278754	24279196	97915400	442	7	432
88	5'-3'	25500208	25500208	25500622	99136854	414	21	404
89	5'-3'	26447610	26447610	26447959	100084256	349	20	339
90	5'-3'	26631180	26631180	26631587	100267826	407	25	397
91	5'-3'	28131484	28131484	28131845	101768130	361	10	351
92	5'-3'	30420825	30420825	30421209	104057471	384	13	374
93	5'-3'	31966306	31966306	31966760	105602952	454	21	444
94	5'-3'	34496414	34496414	34496806	108133060	392	17	382
95	5'-3'	34661212	34661212	34661667	108297858	455	10	445
96	5'-3'	40339845	40339845	40340202	113976491	357	22	347
97	5'-3'	48982173	48982173	48982591	122618819	418	15	408
98	5'-3'	53178506	53178506	53178912	126815152	406	13	396
99	5'-3'	55841647	55841647	55842053	129478293	406	9	396
100	3'-5'	937551	937953	938184	74574197	231	25	221
101	3'-5'	1278551	1278817	1279184	74915197	367	21	357
102	3'-5'	1484961	1485229	1485594	75121607	365	26	355
103	3'-5'	1746785	1747094	1747418	75383431	324	14	314
104	3'-5'	2041759	2042024	2042392	75678405	368	18	358
105	3'-5'	5566537	5566676	5567170	79203183	494	22	484
106	3'-5'	8667957	8668160	8668590	82304603	430	17	420
107	3'-5'	13479575	13479918	13480208	87116221	290	30	280

	108	3'-5'	24487268	24487643	24487901	98123914	258	12	248
	109	3'-5'	24677409	24677691	24678042	98314055	351	7	341
	110	3'-5'	25517642	25517902	25518275	99154288	373	26	363
	111	3'-5'	26388341	26388601	26388974	100024987	373	9	363
	112	3'-5'	27342086	27342340	27342719	100978732	379	12	369
	113	3'-5'	27791135	27791396	27791768	101427781	372	11	362
	114	3'-5'	34217740	34218064	34218373	107854386	309	21	299
	115	3'-5'	36337076	36337287	36337709	109973722	422	28	412
	116	3'-5'	38270643	38270955	38271276	111907289	321	26	311
	117	3'-5'	38646656	38646886	38647289	112283302	403	13	393
	118	3'-5'	40843895	40844009	40844528	114480541	519	17	509
	119	3'-5'	42620089	42620376	42620722	116256735	346	19	336
	120	3'-5'	44369654	44369902	44370287	118006300	385	16	375
	121	3'-5'	46347027	46347229	46347660	119983673	431	30	421
	122	3'-5'	55194362	55194609	55194995	128831008	386	19	376
9			NT_039471.7 (1)						
	0	5'-3'	1721178	1721178	1721353	4721178	175	18	165
	1	5'-3'	1913244	1913244	1913411	4913244	167	27	157
	2	5'-3'	3013998	3013998	3014538	6013998	540	9	530
	3	5'-3'	3994726	3994726	3994896	6994726	170	9	160
	4	5'-3'	4137686	4137686	4138065	7137686	379	23	369
	5	5'-3'	5132266	5132266	5132499	8132266	233	24	223
	6	5'-3'	7125879	7125879	7126394	10125879	515	31	505
	7	5'-3'	7435332	7435332	7435641	10435332	309	28	299
	8	5'-3'	9590057	9590057	9590298	12590057	241	14	231
	9	3'-5'	526313	526405	526946	3526313	541	16	531
	10	3'-5'	1455699	1455930	1456332	4455699	402	12	392
	11	3'-5'	3454558	3454911	3455191	6454558	280	12	270
	12	3'-5'	6248699	6248966	6249332	9248699	366	11	356
	13	3'-5'	6859961	6860038	6860594	9859961	556	16	546
	14	3'-5'	8238010	8238260	8238643	11238010	383	22	373
	15	3'-5'	9481519	9481711	9482152	12481519	441	27	431
			NT_039472.7 (2)						
	16	5'-3'	1307591	1307591	1307990	14525114	399	30	389
	17	5'-3'	4694637	4694637	4695057	17912160	420	17	410

18	5'-3'	5727588	5727588	5728064	18945111	476	15	466
19	5'-3'	6711668	6711668	6712049	19929191	381	26	371
20	5'-3'	9595412	9595412	9595731	22812935	319	8	309
21	5'-3'	9975684	9975684	9976096	23193207	412	23	402
22	5'-3'	11000935	11000935	11001285	24218458	350	26	340
23	5'-3'	11806190	11806190	11806735	25023713	545	28	535
24	5'-3'	17852642	17852642	17852983	31070165	341	21	331
25	5'-3'	21507590	21507590	21507925	34725113	335	12	325
26	5'-3'	24089855	24089855	24090218	37307378	363	7	353
27	5'-3'	26026483	26026483	26027046	39244006	563	21	553
28	5'-3'	38538620	38538620	38538999	51756143	379	13	369
29	3'-5'	381855	382023	382488	13599378	465	17	455
30	3'-5'	1091508	1091824	1092141	14309031	317	9	307
31	3'-5'	2781874	2781928	2782507	15999397	579	7	569
32	3'-5'	4174803	4175008	4175436	17392326	428	12	418
33	3'-5'	5149159	5149408	5149792	18366682	384	10	374
34	3'-5'	6619319	6619609	6619952	19836842	343	31	333
35	3'-5'	6656495	6656811	6657128	19874018	317	6	307
36	3'-5'	8750662	8750872	8751295	21968185	423	9	413
37	3'-5'	11720636	11720921	11721269	24938159	348	22	338
38	3'-5'	14703511	14703642	14704144	27921034	502	24	492
39	3'-5'	16198649	16198863	16199282	29416172	419	17	409
40	3'-5'	18673677	18673941	18674310	31891200	369	34	359
41	3'-5'	22156028	22156423	22156661	35373551	238	23	228
42	3'-5'	24417605	24417823	24418238	37635128	415	24	405
43	3'-5'	25221575	25221853	25222208	38439098	355	13	345
44	3'-5'	25848152	25848415	25848785	39065675	370	8	360
45	3'-5'	28745588	28745949	28746221	41963111	272	34	262
46	3'-5'	31441702	31441962	31442335	44659225	373	30	363
		NT_039474.7 (3)						
47	5'-3'	724815	724815	725220	54304231	405	13	395
48	3'-5'	885462	885683	886095	54464878	412	12	402
49	3'-5'	1129066	1129317	1129699	54708482	382	21	372
		NT_039476.7 (4)						
50	5'-3'	457204	457204	457590	80785374	386	15	376

51	5'-3'	921671	921671	922065	81249841	394	17	384
52	5'-3'	4580912	4580912	4581267	84909082	355	25	345
53	5'-3'	6608387	6608387	6608779	86936557	392	26	382
54	5'-3'	8212792	8212792	8213313	88540962	521	30	511
55	5'-3'	10025510	10025510	10025876	90353680	366	14	356
56	5'-3'	10106951	10106951	10107384	90435121	433	9	423
57	5'-3'	13626236	13626236	13626687	93954406	451	12	441
58	5'-3'	16276545	16276545	16276812	96604715	267	10	257
59	5'-3'	17042935	17042935	17043281	97371105	346	9	336
60	5'-3'	20407340	20407340	20407501	100735510	161	17	151
61	3'-5'	244140	244328	244773	80572310	445	14	435
62	3'-5'	2513230	2513489	2513863	82841400	374	31	364
63	3'-5'	2726351	2726578	2726984	83054521	406	8	396
64	3'-5'	8580060	8580172	8580693	88908230	521	30	511
65	3'-5'	9559283	9559687	9559916	89887453	229	22	219
66	3'-5'	10440318	10440400	10440951	90768488	551	22	541
67	3'-5'	11017005	11017263	11017638	91345175	375	11	365
68	3'-5'	12774842	12775060	12775475	93103012	415	11	405
69	3'-5'	18397153	18397447	18397786	98725323	339	25	329
70	3'-5'	20462985	20463229	20463618	100791155	389	23	379
		NT_039477.7 (5)						
71	5'-3'	3643853	3643853	3644200	104638130	347	15	337
72	5'-3'	3914399	3914399	3914900	104908676	501	7	491
73	3'-5'	1522923	1523178	1523556	102517200	378	23	368
74	3'-5'	3747775	3747985	3748408	104742052	423	29	413
75	3'-5'	4486477	4486720	4487110	105480754	390	17	380
		NT_039482.7 (6)						
76	5'-3'	241419	241419	241711	109055893	292	28	282
77	5'-3'	917781	917781	918173	109732255	392	12	382
78	5'-3'	3585010	3585010	3585428	112399484	418	21	408
79	5'-3'	4327659	4327659	4328035	113142133	376	18	366
80	5'-3'	4880275	4880275	4880661	113694749	386	21	376
81	5'-3'	9154101	9154101	9154392	117968575	291	20	281
82	3'-5'	5816335	5816496	5816968	114630809	472	31	462
83	3'-5'	9794562	9794829	9795195	118609036	366	27	356

	84	3'-5'	10101476	10101524	10102109	118915950	585	12	575
	85	3'-5'	10487785	10488036	10488418	119302259	382	10	372
	86	3'-5'	12945559	12945798	12946192	121760033	394	8	384
	87	3'-5'	13173176	13173496	13173809	121987650	313	7	303
10			NT_03490.7 (1)						
	0	5'-3'	737141	737141	737522	3737141	381	18	371
	1	5'-3'	984084	984084	984556	3984084	472	16	462
	2	5'-3'	1381290	1381290	1381540	4381290	250	7	240
	3	5'-3'	3165812	3165812	3166225	6165812	413	14	403
	4	5'-3'	4190169	4190169	4190628	7190169	459	10	449
	5	3'-5'	1431211	1431320	1431844	4431211	524	11	514
	6	3'-5'	1813346	1813665	1813979	4813346	314	9	304
	7	3'-5'	2090629	2090907	2091262	5090629	355	6	345
	8	3'-5'	3491364	3491649	3491997	6491364	348	21	338
			NT_039492.7 (2)						
	9	5'-3'	5340923	5340923	5341401	12637465	478	19	468
	10	5'-3'	11369726	11369726	11370047	18666268	321	14	311
	11	5'-3'	17456101	17456101	17456526	24752643	425	27	415
	12	5'-3'	20925371	20925371	20925741	28221913	370	27	360
	13	5'-3'	22375198	22375198	22375789	29671740	591	19	581
	14	5'-3'	25421579	25421579	25422089	32718121	510	9	500
	15	5'-3'	27681454	27681454	27681834	34977996	380	12	370
	16	5'-3'	27974353	27974353	27974736	35270895	383	23	373
	17	5'-3'	30181845	30181845	30182193	37478387	348	26	338
	18	5'-3'	30965028	30965028	30965478	38261570	450	18	440
	19	5'-3'	31783250	31783250	31783633	39079792	383	13	373
	20	5'-3'	32515367	32515367	32515988	39811909	621	29	611
	21	5'-3'	33919223	33919223	33919711	41215765	488	30	478
	22	5'-3'	34795513	34795513	34795876	42092055	363	20	353
	23	5'-3'	36643050	36643050	36643295	43939592	245	20	235
	24	5'-3'	38847967	38847967	38848350	46144509	383	25	373
	25	5'-3'	38850237	38850237	38850576	46146779	339	26	329
	26	5'-3'	41246019	41246019	41246150	48542561	131	17	121
	27	5'-3'	46373050	46373050	46373323	53669592	273	18	263
	28	5'-3'	47157634	47157634	47158129	54454176	495	18	485

29	5'-3'	47415509	47415509	47415854	54712051	345	30	335
30	5'-3'	47634516	47634516	47634739	54931058	223	15	213
31	5'-3'	49111779	49111779	49112108	56408321	329	12	319
32	3'-5'	3327043	3327452	3327676	10623585	224	22	214
33	3'-5'	4102616	4102927	4103249	11399158	322	13	312
34	3'-5'	8914747	8914997	8915380	16211289	383	31	373
35	3'-5'	9659687	9659988	9660320	16956229	332	32	322
36	3'-5'	12666058	12666416	12666691	19962600	275	18	265
37	3'-5'	13784254	13784482	13784887	21080796	405	26	395
38	3'-5'	15259113	15259514	15259746	22555655	232	14	222
39	3'-5'	16233580	16233785	16234213	23530122	428	11	418
40	3'-5'	18576441	18576869	18577074	25872983	205	6	195
41	3'-5'	20160852	20161049	20161485	27457394	436	11	426
42	3'-5'	20650049	20650298	20650682	27946591	384	28	374
43	3'-5'	21781865	21782086	21782498	29078407	412	18	402
44	3'-5'	21904003	21904238	21904636	29200545	398	31	388
45	3'-5'	23227845	23228076	23228478	30524387	402	9	392
46	3'-5'	24066901	24066943	24067534	31363443	591	25	581
47	3'-5'	24839129	24839447	24839762	32135671	315	30	305
48	3'-5'	25118161	25118451	25118794	32414703	343	19	333
49	3'-5'	25196265	25196540	25196898	32492807	358	34	348
50	3'-5'	25760305	25760632	25760938	33056847	306	9	296
51	3'-5'	26218802	26219120	26219435	33515344	315	6	305
52	3'-5'	26497728	26498043	26498361	33794270	318	19	308
53	3'-5'	26544050	26544308	26544683	33840592	375	22	365
54	3'-5'	27675245	27675434	27675878	34971787	444	28	434
55	3'-5'	27822583	27822829	27823216	35119125	387	13	377
56	3'-5'	31271469	31271718	31272102	38568011	384	27	374
57	3'-5'	31516860	31517152	31517493	38813402	341	20	331
58	3'-5'	32310368	32310630	32311001	39606910	371	26	361
59	3'-5'	35205991	35206232	35206624	42502533	392	29	382
60	3'-5'	37366833	37367103	37367466	44663375	363	18	353
61	3'-5'	38707506	38707725	38708139	46004048	414	13	404
62	3'-5'	38881274	38881467	38881907	46177816	440	10	430
63	3'-5'	40319549	40319814	40320182	47616091	368	11	358
64	3'-5'	41057670	41058009	41058303	48354212	294	23	284
65	3'-5'	41240708	41240880	41241341	48537250	461	32	451

66	3'-5'	42587423	42587656	42588056	49883965	400	12	390
67	3'-5'	42620165	42620434	42620798	49916707	364	25	354
68	3'-5'	43658564	43658744	43659197	50955106	453	28	443
69	3'-5'	44144911	44145157	44145544	51441453	387	24	377
70	3'-5'	45273579	45273756	45274212	52570121	456	15	446
71	3'-5'	45745033	45745121	45745666	53041575	545	17	535
72	3'-5'	46313290	46313501	46313923	53609832	422	15	412
73	3'-5'	48948012	48948086	48948645	56244554	559	14	549
74	3'-5'	48975013	48975218	48975646	56271555	428	19	418
75	3'-5'	50266018	50266500	50266651	57562560	151	33	141
		NT_039500.7 (3)						
76	5'-3'	326790	326790	327083	57995403	293	6	283
77	5'-3'	2468546	2468546	2468906	60137159	360	27	350
78	5'-3'	5099166	5099166	5099784	62767779	618	9	608
79	5'-3'	7001699	7001699	7001875	64670312	176	10	166
80	5'-3'	7612264	7612264	7612614	65280877	350	31	340
81	5'-3'	8106871	8106871	8107211	65775484	340	24	330
82	5'-3'	8617332	8617332	8617509	66285945	177	14	167
83	5'-3'	10458301	10458301	10458716	68126914	415	30	405
84	5'-3'	14684858	14684858	14685283	72353471	425	9	415
85	5'-3'	19130788	19130788	19131000	76799401	212	17	202
86	5'-3'	19889709	19889709	19890085	77558322	376	17	366
87	5'-3'	21703259	21703259	21703748	79371872	489	26	479
88	5'-3'	24947156	24947156	24947528	82615769	372	22	362
89	5'-3'	27561475	27561475	27561711	85230088	236	28	226
90	5'-3'	29178285	29178285	29178621	86846898	336	17	326
91	5'-3'	30825859	30825859	30826223	88494472	364	27	354
92	5'-3'	33193103	33193103	33193533	90861716	430	10	420
93	5'-3'	39951403	39951403	39951742	97620016	339	18	329
94	5'-3'	40139819	40139819	40140294	97808432	475	28	465
95	5'-3'	42530113	42530113	42530370	100198726	257	14	247
96	5'-3'	43480071	43480071	43480527	101148684	456	23	446
97	5'-3'	45167722	45167722	45168109	102836335	387	26	377
98	5'-3'	45777820	45777820	45778179	103446433	359	24	349
99	5'-3'	46702536	46702536	46702956	104371149	420	7	410
100	5'-3'	47296674	47296674	47296979	104965287	305	18	295

101	5'-3'	50708467	50708467	50708799	108377080	332	30	322
102	5'-3'	53719999	53719999	53720324	111388612	325	27	315
103	5'-3'	58793476	58793476	58793857	116462089	381	16	371
104	5'-3'	62760067	62760067	62760218	120428680	151	24	141
105	5'-3'	62891727	62891727	62892120	120560340	393	29	383
106	5'-3'	64757670	64757670	64758119	122426283	449	7	439
107	5'-3'	66100245	66100245	66100596	123768858	351	28	341
108	5'-3'	66336020	66336020	66336340	124004633	320	24	310
109	5'-3'	67208574	67208574	67208921	124877187	347	30	337
110	3'-5'	4925899	4926141	4926532	62594512	391	16	381
111	3'-5'	5232272	5232644	5232905	62900885	261	23	251
112	3'-5'	5790027	5790310	5790660	63458640	350	18	340
113	3'-5'	6270052	6270294	6270685	63938665	391	19	381
114	3'-5'	6450257	6450729	6450890	64118870	161	7	151
115	3'-5'	8205977	8206389	8206610	65874590	221	16	211
116	3'-5'	9604421	9604696	9605054	67273034	358	9	348
117	3'-5'	13397209	13397460	13397842	71065822	382	19	372
118	3'-5'	15361112	15361450	15361745	73029725	295	16	285
119	3'-5'	20373829	20374198	20374462	78042442	264	20	254
120	3'-5'	21099184	21099516	21099817	78767797	301	34	291
121	3'-5'	24290391	24290573	24291024	81959004	451	22	441
122	3'-5'	28705418	28705556	28706051	86374031	495	25	485
123	3'-5'	30413566	30413760	30414199	88082179	439	9	429
124	3'-5'	34499013	34499121	34499646	92167626	525	9	515
125	3'-5'	34792914	34793202	34793547	92461527	345	34	335
126	3'-5'	35266787	35267037	35267420	92935400	383	8	373
127	3'-5'	38054679	38054887	38055312	95723292	425	21	415
128	3'-5'	40555258	40555554	40555891	98223871	337	29	327
129	3'-5'	41251930	41252118	41252563	98920543	445	9	435
130	3'-5'	42228983	42229191	42229616	99897596	425	26	415
131	3'-5'	43145638	43145889	43146271	100814251	382	20	372
132	3'-5'	43272279	43272525	43272912	100940892	387	10	377
133	3'-5'	44116550	44116731	44117183	101785163	452	30	442
134	3'-5'	44920359	44920571	44920992	102588972	421	20	411
135	3'-5'	45506486	45506737	45507119	103175099	382	12	372
136	3'-5'	46460077	46460242	46460710	104128690	468	13	458
137	3'-5'	48137567	48137820	48138200	105806180	380	13	370

	138	3'-5'	48711214	48711473	48711847	106379827	374	25	364
	139	3'-5'	51072819	51073089	51073452	108741432	363	20	353
	140	3'-5'	52089273	52089629	52089906	109757886	277	9	267
	141	3'-5'	52587505	52587749	52588138	110256118	389	30	379
	142	3'-5'	53561132	53561425	53561765	111229745	340	17	330
	143	3'-5'	55030844	55031100	55031477	112699457	377	31	367
	144	3'-5'	55039705	55039930	55040338	112708318	408	21	398
	145	3'-5'	59321971	59322297	59322604	116990584	307	12	297
	146	3'-5'	62418683	62418936	62419316	120087296	380	9	370
	147	3'-5'	65805704	65805952	65806337	123474317	385	17	375
	148	3'-5'	65977698	65977934	65978331	123646311	397	6	387
	149	3'-5'	70592037	70592308	70592670	128260650	362	9	352
	150	3'-5'	71714268	71714534	71714901	129382881	367	27	357
	151	3'-5'	71715162	71715364	71715795	129383775	431	32	421
11			NT_039515.6 (1)						
	0	5'-3'	95810	95810	96392	3095810	582	14	572
	1	5'-3'	2200663	2200663	2200947	5200663	284	17	274
	2	5'-3'	3683866	3683866	3684245	6683866	379	30	369
	3	5'-3'	5522377	5522377	5522527	8522377	150	28	140
	4	5'-3'	11627435	11627435	11627857	14627435	422	18	412
	5	5'-3'	13214471	13214471	13214885	16214471	414	29	404
	6	5'-3'	15222815	15222815	15223190	18222815	375	14	365
	7	5'-3'	19159921	19159921	19160187	22159921	266	9	256
	8	5'-3'	21244257	21244257	21244597	24244257	340	7	330
	9	5'-3'	28470633	28470633	28471013	31470633	380	14	370
	10	5'-3'	28876641	28876641	28876976	31876641	335	15	325
	11	5'-3'	29306480	29306480	29307087	32306480	607	8	597
	12	3'-5'	9638797	9639087	9639430	12638797	343	6	333
	13	3'-5'	10348174	10348431	10348807	13348174	376	11	366
	14	3'-5'	10752194	10752380	10752827	13752194	447	20	437
	15	3'-5'	11477881	11478058	11478514	14477881	456	8	446
	16	3'-5'	11503101	11503354	11503734	14503101	380	17	370
	17	3'-5'	12270494	12270666	12271127	15270494	461	19	451
	18	3'-5'	14227574	14227935	14228207	17227574	272	6	262
	19	3'-5'	14530511	14530775	14531144	17530511	369	23	359
	20	3'-5'	18839794	18840087	18840427	21839794	340	9	330

21	3'-5'	21885322	21885589	21885955	24885322	366	9	356
22	3'-5'	22629627	22629806	22630260	25629627	454	12	444
23	3'-5'	23213637	23213943	23214270	26213637	327	24	317
24	3'-5'	25013560	25013874	25014193	28013560	319	18	309
25	3'-5'	26614581	26615028	26615214	29614581	186	33	176
26	3'-5'	27163988	27164220	27164621	30163988	401	27	391
27	3'-5'	27307248	27307558	27307881	30307248	323	34	313
28	3'-5'	28819312	28819616	28819945	31819312	329	8	319
		NT_096135.5 (2)						
29	5'-3'	2201812	2201812	2202294	36696934	482	26	472
30	5'-3'	4049377	4049377	4049734	38544499	357	15	347
31	5'-3'	4839368	4839368	4839802	39334490	434	23	424
32	5'-3'	7088861	7088861	7089332	41583983	471	19	461
33	5'-3'	8181709	8181709	8182101	42676831	392	20	382
34	5'-3'	11193078	11193078	11193565	45688200	487	7	477
35	5'-3'	13358238	13358238	13358686	47853360	448	24	438
36	5'-3'	13695279	13695279	13695698	48190401	419	9	409
37	5'-3'	14066627	14066627	14067046	48561749	419	30	409
38	5'-3'	14943976	14943976	14944457	49439098	481	14	471
39	5'-3'	20343346	20343346	20343673	54838468	327	15	317
40	5'-3'	23373693	23373693	23374053	57868815	360	8	350
41	5'-3'	28812452	28812452	28812632	63307574	180	15	170
42	5'-3'	32165994	32165994	32166404	66661116	410	10	400
43	5'-3'	34372108	34372108	34372634	68867230	526	17	516
44	5'-3'	36218131	36218131	36218586	70713253	455	12	445
45	5'-3'	39203329	39203329	39203682	73698451	353	14	343
46	5'-3'	39205535	39205535	39205828	73700657	293	18	283
47	5'-3'	41508139	41508139	41508623	76003261	484	17	474
48	5'-3'	45907969	45907969	45908421	80403091	452	8	442
49	5'-3'	46131613	46131613	46132060	80626735	447	29	437
50	5'-3'	47405386	47405386	47405784	81900508	398	6	388
51	5'-3'	50751925	50751925	50752397	85247047	472	7	462
52	5'-3'	52401151	52401151	52401511	86896273	360	28	350
53	5'-3'	53107377	53107377	53107846	87602499	469	16	459
54	3'-5'	282966	283382	283599	34778088	217	20	207
55	3'-5'	1099274	1099646	1099907	35594396	261	20	251

56	3'-5'	3408047	3408317	3408680	37903169	363	30	353
57	3'-5'	3493057	3493374	3493690	37988179	316	30	306
58	3'-5'	3505439	3505641	3506072	38000561	431	9	421
59	3'-5'	4051560	4051846	4052193	38546682	347	8	337
60	3'-5'	4870830	4871011	4871463	39365952	452	29	442
61	3'-5'	8096498	8096730	8097131	42591620	401	33	391
62	3'-5'	8351892	8352023	8352525	42847014	502	11	492
63	3'-5'	13812724	13812931	13813357	48307846	426	14	416
64	3'-5'	14596112	14596194	14596745	49091234	551	20	541
65	3'-5'	17552822	17552867	17553455	52047944	588	32	578
66	3'-5'	17728642	17729056	17729275	52223764	219	31	209
67	3'-5'	19765007	19765199	19765640	54260129	441	23	431
68	3'-5'	21826927	21827018	21827560	56322049	542	28	532
69	3'-5'	23719217	23719476	23719850	58214339	374	6	364
70	3'-5'	30528793	30529052	30529426	65023915	374	7	364
71	3'-5'	34522267	34522555	34522900	69017389	345	9	335
72	3'-5'	36750900	36751156	36751533	71246022	377	6	367
73	3'-5'	44496605	44496925	44497238	78991727	313	27	303
		NT_165773.2 (3)						
74	5'-3'	2790383	2790383	2790789	91249308	406	15	396
75	5'-3'	3479811	3479811	3480242	91938736	431	21	421
76	5'-3'	4143840	4143840	4144258	92602765	418	15	408
77	5'-3'	12909805	12909805	12910144	101368730	339	7	329
78	5'-3'	13263310	13263310	13263536	101722235	226	22	216
79	5'-3'	18237142	18237142	18237491	106696067	349	11	339
80	5'-3'	20747663	20747663	20747955	109206588	292	16	282
81	5'-3'	22547051	22547051	22547457	111005976	406	8	396
82	5'-3'	26922833	26922833	26923134	115381758	301	16	291
83	5'-3'	29031751	29031751	29031997	117490676	246	19	236
84	3'-5'	3242148	3242604	3242781	91701073	177	7	167
85	3'-5'	5128107	5128352	5128740	93587032	388	9	378
86	3'-5'	6457945	6458174	6458578	94916870	404	8	394
87	3'-5'	9288059	9288420	9288692	97746984	272	21	262
88	3'-5'	10564786	10564947	10565419	99023711	472	24	462
89	3'-5'	12148410	12148749	12149043	100607335	294	26	284
90	3'-5'	12253734	12253918	12254367	100712659	449	9	439

	91	3'-5'	15972762	15972929	15973395	104431687	466	22	456
	92	3'-5'	16204968	16205287	16205601	104663893	314	13	304
	93	3'-5'	22610826	22610968	22611459	111069751	491	31	481
	94	3'-5'	23024119	23024345	23024752	111483044	407	20	397
	95	3'-5'	23751672	23751785	23752305	112210597	520	33	510
	96	3'-5'	24848958	24849152	24849591	113307883	439	27	429
	97	3'-5'	31913336	31913608	31913969	120372261	361	7	351
12			NT_039548.7 (1)						
	0	5'-3'	520783	520783	521393	3520783	610	9	600
	1	5'-3'	3151738	3151738	3152070	6151738	332	8	322
	2	5'-3'	4481405	4481405	4481758	7481405	353	25	343
	3	5'-3'	5213886	5213886	5214285	8213886	399	23	389
	4	5'-3'	6879772	6879772	6880157	9879772	385	23	375
	5	5'-3'	12114096	12114096	12114406	15114096	310	22	300
	6	5'-3'	14960348	14960348	14960709	17960348	361	11	351
	7	5'-3'	15493886	15493886	15494241	18493886	355	11	345
	8	5'-3'	15598510	15598510	15598870	18598510	360	11	350
	9	5'-3'	15716872	15716872	15717231	18716872	359	11	349
	10	5'-3'	15832684	15832684	15833044	18832684	360	11	350
	11	5'-3'	15958036	15958036	15958397	18958036	361	11	351
	12	5'-3'	17399422	17399422	17399777	20399422	355	11	345
	13	5'-3'	17592451	17592451	17592839	20592451	388	11	378
	14	5'-3'	17969580	17969580	17969940	20969580	360	11	350
	15	5'-3'	18093411	18093411	18093772	21093411	361	11	351
	16	5'-3'	19664393	19664393	19664754	22664393	361	11	351
	17	5'-3'	19781596	19781596	19781957	22781596	361	11	351
	18	5'-3'	19952348	19952348	19952710	22952348	362	11	352
	19	5'-3'	20074427	20074427	20074788	23074427	361	11	351
	20	5'-3'	20498640	20498640	20499001	23498640	361	11	351
	21	5'-3'	20635128	20635128	20635489	23635128	361	11	351
	22	5'-3'	21581052	21581052	21581420	24581052	368	9	358
	23	5'-3'	22619556	22619556	22619968	25619556	412	9	402
	24	5'-3'	26042398	26042398	26042768	29042398	370	19	360
	25	5'-3'	28163959	28163959	28164321	31163959	362	17	352
	26	5'-3'	31612510	31612510	31612931	34612510	421	13	411
	27	5'-3'	36496206	36496206	36496535	39496206	329	15	319

28	5'-3'	37452360	37452360	37452702	40452360	342	6	332
29	5'-3'	38382406	38382406	38382760	41382406	354	31	344
30	3'-5'	3332283	3332588	3332916	6332283	328	26	318
31	3'-5'	5111652	5111683	5112285	8111652	602	16	592
32	3'-5'	16776130	16776401	16776763	19776130	362	11	352
33	3'-5'	16918589	16918862	16919222	19918589	360	11	350
34	3'-5'	18865639	18865917	18866272	21865639	355	11	345
35	3'-5'	19115715	19115987	19116348	22115715	361	11	351
36	3'-5'	19233150	19233422	19233783	22233150	361	11	351
37	3'-5'	19531493	19531765	19532126	22531493	361	11	351
38	3'-5'	20448341	20448606	20448974	23448341	368	9	358
39	3'-5'	20877728	20878000	20878361	23877728	361	11	351
40	3'-5'	21354205	21354477	21354838	24354205	361	11	351
41	3'-5'	21514097	21514369	21514730	24514097	361	11	351
42	3'-5'	21807689	21807962	21808322	24807689	360	11	350
43	3'-5'	21959748	21960020	21960381	24959748	361	11	351
44	3'-5'	23840689	23841092	23841322	26840689	230	24	220
45	3'-5'	25777810	25778185	25778443	28777810	258	13	248
46	3'-5'	27932302	27932435	27932935	30932302	500	21	490
47	3'-5'	27939782	27939995	27940415	30939782	420	24	410
48	3'-5'	28015555	28015731	28016188	31015555	457	27	447
49	3'-5'	28478400	28478653	28479033	31478400	380	12	370
		NT_039551.7 (2)						
50	5'-3'	464656	464656	464997	42796542	341	19	331
51	5'-3'	1375861	1375861	1376200	43707747	339	27	329
52	5'-3'	3775248	3775248	3775625	46107134	377	17	367
53	5'-3'	6611074	6611074	6611430	48942960	356	27	346
54	5'-3'	8167512	8167512	8167919	50499398	407	31	397
55	5'-3'	8530948	8530948	8531274	50862834	326	12	316
56	5'-3'	9592456	9592456	9592852	51924342	396	17	386
57	5'-3'	10046404	10046404	10046804	52378290	400	12	390
58	5'-3'	11029923	11029923	11030356	53361809	433	26	423
59	5'-3'	11281674	11281674	11282024	53613560	350	15	340
60	5'-3'	14900232	14900232	14900609	57232118	377	9	367
61	5'-3'	15302831	15302831	15303256	57634717	425	28	415
62	5'-3'	15729306	15729306	15729701	58061192	395	12	385

63	5'-3'	16805441	16805441	16805844	59137327	403	17	393
64	5'-3'	17385649	17385649	17386061	59717535	412	13	402
65	5'-3'	22775782	22775782	22776071	65107668	289	6	279
66	5'-3'	24133003	24133003	24133379	66464889	376	19	366
67	5'-3'	25065613	25065613	25066049	67397499	436	13	426
68	5'-3'	27994323	27994323	27994901	70326209	578	9	568
69	5'-3'	28450787	28450787	28451137	70782673	350	26	340
70	5'-3'	29750851	29750851	29751198	72082737	347	6	337
71	5'-3'	33628176	33628176	33628572	75960062	396	11	386
72	5'-3'	33749301	33749301	33749668	76081187	367	27	357
73	5'-3'	33755497	33755497	33755933	76087383	436	9	426
74	5'-3'	35909098	35909098	35909497	78240984	399	20	389
75	3'-5'	456564	456945	457197	42788450	252	32	242
76	3'-5'	500825	501092	501458	42832711	366	9	356
77	3'-5'	2704788	2705040	2705421	45036674	381	8	371
78	3'-5'	6972486	6972852	6973119	49304372	267	29	257
79	3'-5'	12570352	12570720	12570985	54902238	265	29	255
80	3'-5'	19215914	19216170	19216547	61547800	377	13	367
81	3'-5'	21318730	21319006	21319363	63650616	357	25	347
82	3'-5'	21366066	21366336	21366699	63697952	363	25	353
83	3'-5'	24574037	24574270	24574670	66905923	400	26	390
84	3'-5'	24816019	24816344	24816652	67147905	308	31	298
85	3'-5'	25052539	25052849	25053172	67384425	323	8	313
86	3'-5'	25130887	25131128	25131520	67462773	392	34	382
87	3'-5'	26184434	26184642	26185067	68516320	425	19	415
88	3'-5'	26976650	26977005	26977283	69308536	278	8	268
89	3'-5'	28764920	28765275	28765553	71096806	278	17	268
90	3'-5'	30469783	30470216	30470416	72801669	200	14	190
91	3'-5'	30944537	30944832	30945170	73276423	338	30	328
92	3'-5'	32250033	32250279	32250666	74581919	387	10	377
93	3'-5'	33039660	33039932	33040293	75371546	361	28	351
94	3'-5'	34577641	34577817	34578274	76909527	457	22	447
95	3'-5'	35724436	35724772	35725069	78056322	297	17	287
96	3'-5'	36470826	36471094	36471459	78802712	365	24	355
97	3'-5'	42621853	42622146	42622486	84953739	340	20	330

NT_166318.1 (3)

	98	5'-3'	766784	766784	767153	89470777	369	11	359
	99	5'-3'	3291977	3291977	3292534	91995970	557	19	547
	100	5'-3'	5532894	5532894	5533369	94236887	475	30	465
	101	5'-3'	5936094	5936094	5936470	94640087	376	29	366
	102	5'-3'	8743614	8743614	8744029	97447607	415	6	405
	103	5'-3'	9059529	9059529	9059930	97763522	401	15	391
	104	5'-3'	11624278	11624278	11624531	100328271	253	29	243
	105	5'-3'	12814785	12814785	12815133	101518778	348	11	338
	106	5'-3'	13495679	13495679	13496069	102199672	390	31	380
	107	5'-3'	13741547	13741547	13741939	102445540	392	11	382
	108	5'-3'	23192458	23192458	23192725	111896451	267	14	257
	109	5'-3'	28223059	28223059	28223535	116927052	476	18	466
	110	5'-3'	28503783	28503783	28504152	117207776	369	24	359
	111	5'-3'	31243479	31243479	31243815	119947472	336	26	326
	112	3'-5'	2066348	2066637	2066981	90770341	344	14	334
	113	3'-5'	2602372	2602651	2603005	91306365	354	7	344
	114	3'-5'	3134670	3134927	3135303	91838663	376	26	366
	115	3'-5'	5014757	5015260	5015390	93718750	130	6	120
	116	3'-5'	6767857	6767990	6768490	95471850	500	9	490
	117	3'-5'	7028137	7028318	7028770	95732130	452	8	442
	118	3'-5'	7596877	7597132	7597510	96300870	378	28	368
	119	3'-5'	8221636	8221861	8222269	96925629	408	31	398
	120	3'-5'	19244039	19244352	19244672	107948032	320	19	310
	121	3'-5'	20773285	20773387	20773918	109477278	531	33	521
	122	3'-5'	20948265	20948491	20948898	109652258	407	14	397
	123	3'-5'	26763255	26763635	26763888	115467248	253	33	243
13			NT_039573.7 (1)						
	0	5'-3'	7022324	7022324	7022816	10022324	492	16	482
	1	5'-3'	7212977	7212977	7213380	10212977	403	28	393
	2	3'-5'	171025	171282	171658	3171025	376	14	366
	3	3'-5'	1999238	1999456	1999871	4999238	415	9	405
	4	3'-5'	2824538	2824805	2825171	5824538	366	9	356
	5	3'-5'	5631908	5632167	5632541	8631908	374	22	364
	6	3'-5'	6933685	6933888	6934318	9933685	430	11	420
			NT_039578.7 (2)						

7	5'-3'	25743	25743	26109	11199084	366	12	356
8	5'-3'	4280831	4280831	4281218	15454172	387	25	377
9	5'-3'	5667425	5667425	5667800	16840766	375	21	365
10	5'-3'	6630962	6630962	6631340	17804303	378	14	368
11	5'-3'	19412776	19412776	19413149	30586117	373	15	363
12	5'-3'	21775876	21775876	21776256	32949217	380	24	370
13	5'-3'	25139750	25139750	25140191	36313091	441	12	431
14	5'-3'	25736093	25736093	25736375	36909434	282	26	272
15	3'-5'	23419	23771	24052	11196760	281	32	271
16	3'-5'	2730184	2730429	2730817	13903525	388	33	378
17	3'-5'	5427721	5427981	5428354	16601062	373	33	363
18	3'-5'	6641225	6641515	6641858	17814566	343	6	333
19	3'-5'	7638506	7638770	7639139	18811847	369	12	359
20	3'-5'	11457981	11458238	11458614	22631322	376	6	366
21	3'-5'	12035292	12035599	12035925	23208633	326	24	316
22	3'-5'	12105057	12105366	12105690	23278398	324	24	314
23	3'-5'	16897270	16897500	16897903	28070611	403	19	393
24	3'-5'	17377005	17377366	17377638	28550346	272	9	262
25	3'-5'	23073094	23073261	23073727	34246435	466	32	456
26	3'-5'	23106031	23106405	23106664	34279372	259	6	249
27	3'-5'	24057578	24057777	24058211	35230919	434	31	424
28	3'-5'	25681355	25681526	25681988	36854696	462	15	452
29	3'-5'	27580091	27580156	27580724	38753432	568	12	558
		NT_039580.7 (3)						
30	5'-3'	755602	755602	755984	40598538	382	16	372
31	5'-3'	979398	979398	979809	40822334	411	19	401
32	5'-3'	1344404	1344404	1344653	41187340	249	21	239
33	5'-3'	4777529	4777529	4778009	44620465	480	20	470
34	5'-3'	6651407	6651407	6651771	46494343	364	12	354
35	5'-3'	9029273	9029273	9029600	48872209	327	12	317
36	5'-3'	13089269	13089269	13089629	52932205	360	18	350
37	3'-5'	1498543	1498891	1499176	41341479	285	7	275
38	3'-5'	1875065	1875286	1875698	41718001	412	7	402
39	3'-5'	2420994	2421076	2421627	42263930	551	12	541
40	3'-5'	2579042	2579396	2579675	42421978	279	28	269
41	3'-5'	5212169	5212497	5212802	45055105	305	31	295

42	3'-5'	6241266	6241350	6241899	46084202	549	20	539
43	3'-5'	7114493	7114616	7115126	46957429	510	23	500
44	3'-5'	11866557	11866730	11867190	51709493	460	23	450
		NT_039589.7 (4)						
45	5'-3'	571470	571470	571992	55030351	522	19	512
46	5'-3'	3879784	3879784	3880155	58338665	371	12	361
47	5'-3'	8971034	8971034	8971273	63429915	239	21	229
48	5'-3'	12331677	12331677	12332147	66790558	470	28	460
49	5'-3'	12458612	12458612	12459082	66917493	470	28	460
50	5'-3'	13523023	13523023	13523455	67981904	432	25	422
51	5'-3'	14307494	14307494	14307800	68766375	306	26	296
52	5'-3'	17816467	17816467	17816986	72275348	519	25	509
53	5'-3'	20732715	20732715	20733082	75191596	367	27	357
54	5'-3'	21475476	21475476	21475984	75934357	508	19	498
55	5'-3'	28118609	28118609	28118969	82577490	360	17	350
56	5'-3'	31104395	31104395	31104777	85563276	382	6	372
57	5'-3'	33170362	33170362	33170760	87629243	398	10	388
58	5'-3'	35682871	35682871	35683363	90141752	492	26	482
59	3'-5'	6398606	6398838	6399239	60857487	401	14	391
60	3'-5'	6543150	6543436	6543783	61002031	347	20	337
61	3'-5'	10053112	10053321	10053745	64511993	424	22	414
62	3'-5'	12682681	12682891	12683314	67141562	423	33	413
63	3'-5'	21439186	21439386	21439819	75898067	433	18	423
64	3'-5'	21823216	21823445	21823849	76282097	404	28	394
65	3'-5'	23143886	23144182	23144519	77602767	337	7	327
66	3'-5'	25865617	25865694	25866250	80324498	556	20	546
67	3'-5'	30056939	30056959	30057572	84515820	613	16	603
68	3'-5'	31155859	31156229	31156492	85614740	263	20	253
69	3'-5'	34735570	34735915	34736203	89194451	288	25	278
70	3'-5'	36456779	36457009	36457412	90915660	403	6	393
71	3'-5'	37584042	37584132	37584675	92042923	543	12	533
		NT_039590.7 (5)						
72	5'-3'	1067719	1067719	1067856	93859070	137	6	127
73	5'-3'	8008041	8008041	8008254	100799392	213	29	203
74	5'-3'	9337937	9337937	9338328	102129288	391	28	381

	75	5'-3'	12350298	12350298	12350652	105141649	354	26	344
	76	5'-3'	13358572	13358572	13358948	106149923	376	23	366
	77	3'-5'	429688	429969	430321	93221039	352	8	342
	78	3'-5'	1109089	1109345	1109722	93900440	377	6	367
	79	3'-5'	1557161	1557383	1557794	94348512	411	8	401
14			NT_039595.7 (1)						
	0	5'-3'	7203049	7203049	7203274	10203049	225	17	215
	1	5'-3'	7778523	7778523	7778927	10778523	404	18	394
	2	5'-3'	9101270	9101270	9101660	12101270	390	10	380
	3	5'-3'	11964275	11964275	11964662	14964275	387	30	377
	4	3'-5'	7739437	7739745	7740070	10739437	325	23	315
	5	3'-5'	8055378	8055634	8056011	11055378	377	29	367
	6	3'-5'	10622800	10622979	10623433	13622800	454	9	444
	7	3'-5'	15171685	15172017	15172318	18171685	301	31	291
			NT_039596.7 (2)						
	8	5'-3'	4033313	4033313	4033634	24322239	321	15	311
			NT_166324.1 (3)						
	9	3'-5'	13445	13670	14078	25548201	408	9	398
			NT_039606.7 (4)						
	10	5'-3'	591560	591560	592025	28156516	465	6	455
	11	5'-3'	3101477	3101477	3101891	30666433	414	20	404
	12	5'-3'	6496430	6496430	6496695	34061386	265	26	255
	13	5'-3'	10978823	10978823	10979220	38543779	397	11	387
	14	5'-3'	11451976	11451976	11452370	39016932	394	12	384
	15	5'-3'	11762007	11762007	11762537	39326963	530	18	520
	16	5'-3'	11996543	11996543	11996934	39561499	391	13	381
	17	5'-3'	13117790	13117790	13118263	40682746	473	11	463
	18	5'-3'	14130707	14130707	14131202	41695663	495	26	485
	19	5'-3'	20303993	20303993	20304499	47868949	506	21	496
	20	5'-3'	21633130	21633130	21633471	49198086	341	31	331
	21	5'-3'	22018483	22018483	22018631	49583439	148	28	138
	22	5'-3'	22518406	22518406	22518734	50083362	328	7	318
	23	5'-3'	32399939	32399939	32400215	59964895	276	22	266

24	5'-3'	32741743	32741743	32742123	60306699	380	14	370
25	5'-3'	41189403	41189403	41189809	68754359	406	24	396
26	5'-3'	41988777	41988777	41989161	69553733	384	26	374
27	5'-3'	47807879	47807879	47808265	75372835	386	25	376
28	5'-3'	49950672	49950672	49951065	77515628	393	25	383
29	5'-3'	49977970	49977970	49978404	77542926	434	16	424
30	5'-3'	52909556	52909556	52910174	80474512	618	12	608
31	5'-3'	53392049	53392049	53392416	80957005	367	12	357
32	5'-3'	54648376	54648376	54648728	82213332	352	9	342
33	5'-3'	56944195	56944195	56944502	84509151	307	29	297
34	5'-3'	57077117	57077117	57077493	84642073	376	27	366
35	5'-3'	57678029	57678029	57678321	85242985	292	22	282
36	5'-3'	64264516	64264516	64264983	91829472	467	10	457
37	5'-3'	65780026	65780026	65780397	93344982	371	21	361
38	5'-3'	66760687	66760687	66760985	94325643	298	26	288
39	5'-3'	68232866	68232866	68233231	95797822	365	9	355
40	5'-3'	70570442	70570442	70570597	98135398	155	28	145
41	5'-3'	70719105	70719105	70719525	98284061	420	20	410
42	5'-3'	76131436	76131436	76131722	103696392	286	28	276
43	5'-3'	78675599	78675599	78675998	106240555	399	18	389
44	5'-3'	79058198	79058198	79058530	106623154	332	15	322
45	5'-3'	79814808	79814808	79815221	107379764	413	20	403
46	5'-3'	80643705	80643705	80644093	108208661	388	14	378
47	5'-3'	81146246	81146246	81146635	108711202	389	16	379
48	5'-3'	81729040	81729040	81729428	109293996	388	15	378
49	5'-3'	84251537	84251537	84251944	111816493	407	25	397
50	5'-3'	84369256	84369256	84369618	111934212	362	26	352
51	5'-3'	85404484	85404484	85404848	112969440	364	26	354
52	5'-3'	85711013	85711013	85711324	113275969	311	12	301
53	5'-3'	87792026	87792026	87792473	115356982	447	29	437
54	5'-3'	94062265	94062265	94062727	121627221	462	6	452
55	5'-3'	94062316	94062316	94062727	121627272	411	6	401
56	5'-3'	94062367	94062367	94062758	121627323	391	6	381
57	5'-3'	94062418	94062418	94062824	121627374	406	6	396
58	5'-3'	94062469	94062469	94062824	121627425	355	6	345
59	5'-3'	96018278	96018278	96018680	123583234	402	12	392
60	5'-3'	96779732	96779732	96780111	124344688	379	23	369

61	5'-3'	97056996	97056996	97057377	124621952	381	18	371
62	3'-5'	1840226	1840636	1840859	29405182	223	31	213
63	3'-5'	2349297	2349601	2349930	29914253	329	9	319
64	3'-5'	5598964	5599190	5599597	33163920	407	25	397
65	3'-5'	7351531	7351731	7352164	34916487	433	31	423
66	3'-5'	8237228	8237358	8237861	35802184	503	20	493
67	3'-5'	9527078	9527326	9527711	37092034	385	24	375
68	3'-5'	12581369	12581631	12582002	40146325	371	34	361
69	3'-5'	12664665	12664996	12665298	40229621	302	18	292
70	3'-5'	18498414	18498645	18499047	46063370	402	21	392
71	3'-5'	20907772	20907975	20908405	48472728	430	23	420
72	3'-5'	23018342	23018617	23018975	50583298	358	21	348
73	3'-5'	23076798	23077047	23077431	50641754	384	19	374
74	3'-5'	24618899	24619204	24619532	52183855	328	19	318
75	3'-5'	25257348	25257530	25257981	52822304	451	8	441
76	3'-5'	27954614	27954930	27955247	55519570	317	20	307
77	3'-5'	30636061	30636439	30636694	58201017	255	29	245
78	3'-5'	33808461	33808615	33809094	61373417	479	27	469
79	3'-5'	36021479	36021628	36022112	63586435	484	22	474
80	3'-5'	38997766	38998041	38998399	66562722	358	31	348
81	3'-5'	40232840	40232864	40233473	67797796	609	19	599
82	3'-5'	41496039	41496218	41496672	69060995	454	33	444
83	3'-5'	42154526	42154773	42155159	69719482	386	14	376
84	3'-5'	50919943	50920228	50920576	78484899	348	7	338
85	3'-5'	53779204	53779421	53779837	81344160	416	31	406
86	3'-5'	53870715	53870903	53871348	81435671	445	7	435
87	3'-5'	55716832	55717020	55717465	83281788	445	15	435
88	3'-5'	57350585	57350598	57351218	84915541	620	20	610
89	3'-5'	57493515	57493786	57494148	85058471	362	12	352
90	3'-5'	58450152	58450258	58450785	86015108	527	34	517
91	3'-5'	59254683	59254958	59255316	86819639	358	6	348
92	3'-5'	60389760	60390026	60390393	87954716	367	19	357
93	3'-5'	60880572	60880785	60881205	88445528	420	11	410
94	3'-5'	61220161	61220220	61220794	88785117	574	22	564
95	3'-5'	61307494	61307741	61308127	88872450	386	28	376
96	3'-5'	66054948	66055188	66055581	93619904	393	15	383
97	3'-5'	66355824	66356084	66356457	93920780	373	23	363

	98	3'-5'	66908777	66908908	66909410	94473733	502	22	492
	99	3'-5'	68919869	68920124	68920502	96484825	378	22	368
	100	3'-5'	69208631	69208784	69209264	96773587	480	23	470
	101	3'-5'	71924094	71924354	71924727	99489050	373	27	363
	102	3'-5'	73511805	73512109	73512438	101076761	329	9	319
	103	3'-5'	74578091	74578269	74578724	102143047	455	21	445
	104	3'-5'	74688548	74688756	74689181	102253504	425	23	415
	105	3'-5'	76775445	76775692	76776078	104340401	386	27	376
	106	3'-5'	78994730	78994883	78995363	106559686	480	31	470
	107	3'-5'	79030334	79030597	79030967	106595290	370	33	360
	108	3'-5'	80216201	80216490	80216834	107781157	344	8	334
	109	3'-5'	81020439	81020641	81021072	108585395	431	21	421
	110	3'-5'	81156311	81156469	81156944	108721267	475	33	465
	111	3'-5'	81381241	81381609	81381874	108946197	265	26	255
	112	3'-5'	83833802	83834100	83834435	111398758	335	24	325
	113	3'-5'	86564145	86564387	86564778	114129101	391	20	381
	114	3'-5'	86655030	86655293	86655663	114219986	370	26	360
	115	3'-5'	87524351	87524559	87524984	115089307	425	30	415
	116	3'-5'	93048759	93048937	93049392	120613715	455	7	445
	117	3'-5'	94026411	94026509	94027044	121591367	535	25	525
	118	3'-5'	95748238	95748516	95748871	123313194	355	22	345
15			NT_039617.7 (1)						
	0	5'-3'	851336	851336	851610	3851336	274	14	264
	1	5'-3'	3409730	3409730	3410055	6409730	325	20	315
	2	3'-5'	5017730	5017970	5018363	8017730	393	22	383
			NT_039618.7 (2)						
	3	5'-3'	1272301	1272301	1272450	10256829	149	25	139
	4	5'-3'	3236311	3236311	3236674	12220839	363	31	353
	5	5'-3'	5266271	5266271	5266716	14250799	445	13	435
	6	5'-3'	5366406	5366406	5366792	14350934	386	17	376
	7	5'-3'	5919459	5919459	5919974	14903987	515	21	505
	8	5'-3'	6598898	6598898	6599247	15583426	349	28	339
	9	5'-3'	7108152	7108152	7108445	16092680	293	16	283
	10	5'-3'	8283480	8283480	8283787	17268008	307	15	297
	11	5'-3'	9913632	9913632	9914168	18898160	536	27	526

12	5'-3'	10265673	10265673	10266134	19250201	461	8	451
13	5'-3'	14066578	14066578	14067048	23051106	470	28	460
14	5'-3'	15561349	15561349	15561723	24545877	374	19	364
15	5'-3'	18530281	18530281	18530643	27514809	362	12	352
16	5'-3'	19659527	19659527	19659913	28644055	386	13	376
17	5'-3'	23418153	23418153	23418439	32402681	286	28	276
18	5'-3'	23531969	23531969	23532587	32516497	618	15	608
19	5'-3'	23825975	23825975	23826335	32810503	360	19	350
20	5'-3'	25908692	25908692	25909063	34893220	371	16	361
21	5'-3'	26466897	26466897	26467371	35451425	474	13	464
22	5'-3'	27939913	27939913	27940304	36924441	391	24	381
23	3'-5'	130959	131254	131592	9115487	338	11	328
24	3'-5'	1772321	1772681	1772954	10756849	273	28	263
25	3'-5'	1851423	1851450	1852056	10835951	606	17	596
26	3'-5'	4010394	4010583	4011027	12994922	444	11	434
27	3'-5'	5229161	5229422	5229794	14213689	372	14	362
28	3'-5'	5914398	5914502	5915031	14898926	529	25	519
29	3'-5'	7131639	7131969	7132272	16116167	303	18	293
30	3'-5'	7170422	7170707	7171055	16154950	348	24	338
31	3'-5'	7441568	7441772	7442201	16426096	429	16	419
32	3'-5'	12116177	12116392	12116810	21100705	418	22	408
33	3'-5'	12521784	12522027	12522417	21506312	390	34	380
34	3'-5'	12940839	12941081	12941472	21925367	391	27	381
35	3'-5'	13122546	13122805	13123179	22107074	374	9	364
36	3'-5'	13596722	13596973	13597355	22581250	382	26	372
37	3'-5'	14859517	14859788	14860150	23844045	362	8	352
38	3'-5'	15063956	15064280	15064589	24048484	309	27	299
39	3'-5'	15310380	15310508	15311013	24294908	505	28	495
40	3'-5'	17064074	17064295	17064707	26048602	412	11	402
41	3'-5'	18020836	18021083	18021469	27005364	386	23	376
42	3'-5'	24274698	24274855	24275331	33259226	476	7	466
43	3'-5'	27629543	27629795	27630176	36614071	381	6	371
44	3'-5'	27991839	27992152	27992472	36976367	320	34	310
45	3'-5'	29570264	29570368	29570897	38554792	529	28	519
46	3'-5'	29603858	29604135	29604491	38588386	356	22	346

NT_039621.7 (3)

47	5'-3'	141352	141352	141535	38855348	183	17	173
48	5'-3'	348211	348211	348633	39062207	422	25	412
49	5'-3'	521242	521242	521586	39235238	344	31	334
50	5'-3'	573414	573414	573780	39287410	366	7	356
51	5'-3'	1518536	1518536	1518964	40232532	428	13	418
52	5'-3'	3278038	3278038	3278252	41992034	214	9	204
53	5'-3'	5623145	5623145	5623545	44337141	400	21	390
54	5'-3'	11363780	11363780	11364170	50077776	390	9	380
55	5'-3'	11790546	11790546	11790762	50504542	216	12	206
56	5'-3'	15005873	15005873	15006382	53719869	509	16	499
57	5'-3'	16415598	16415598	16415992	55129594	394	10	384
58	5'-3'	20868409	20868409	20868877	59582405	468	21	458
59	5'-3'	22004851	22004851	22005270	60718847	419	25	409
60	5'-3'	24130910	24130910	24131371	62844906	461	18	451
61	5'-3'	26456577	26456577	26456889	65170573	312	27	302
62	5'-3'	31094707	31094707	31095134	69808703	427	6	417
63	5'-3'	33121169	33121169	33121733	71835165	564	12	554
64	5'-3'	33491627	33491627	33492156	72205623	529	31	519
65	5'-3'	34209917	34209917	34210453	72923913	536	31	526
66	5'-3'	36672675	36672675	36672986	75386671	311	15	301
67	5'-3'	38610333	38610333	38610840	77324329	507	24	497
68	5'-3'	46516762	46516762	46517069	85230758	307	20	297
69	5'-3'	51155664	51155664	51156105	89869660	441	24	431
70	5'-3'	54560451	54560451	54560923	93274447	472	23	462
71	5'-3'	57369590	57369590	57369871	96083586	281	23	271
72	5'-3'	60420822	60420822	60421257	99134818	435	16	425
73	5'-3'	61833503	61833503	61833823	100547499	320	17	310
74	5'-3'	62171487	62171487	62171685	100885483	198	9	188
75	3'-5'	3653144	3653423	3653777	42367140	354	15	344
76	3'-5'	3980093	3980294	3980726	42694089	432	18	422
77	3'-5'	4800887	4801227	4801520	43514883	293	24	283
78	3'-5'	5653064	5653172	5653697	44367060	525	30	515
79	3'-5'	7649219	7649495	7649852	46363215	357	19	347
80	3'-5'	8527669	8527894	8528302	47241665	408	19	398
81	3'-5'	9798566	9798834	9799199	48512562	365	10	355
82	3'-5'	10564032	10564276	10564665	49278028	389	18	379
83	3'-5'	11879252	11879529	11879885	50593248	356	27	346

	84	3'-5'	12377945	12378184	12378578	51091941	394	33	384
	85	3'-5'	14703546	14703728	14704179	53417542	451	23	441
	86	3'-5'	17171413	17171865	17172046	55885409	181	13	171
	87	3'-5'	19404408	19404549	19405041	58118404	492	8	482
	88	3'-5'	20634231	20634455	20634864	59348227	409	10	399
	89	3'-5'	22662998	22663252	22663631	61376994	379	25	369
	90	3'-5'	23500779	23500941	23501412	62214775	471	24	461
	91	3'-5'	23871815	23871964	23872448	62585811	484	14	474
	92	3'-5'	26232379	26232568	26233012	64946375	444	11	434
	93	3'-5'	27704358	27704590	27704991	66418354	401	30	391
	94	3'-5'	33184387	33184578	33185020	71898383	442	26	432
	95	3'-5'	38767067	38767190	38767700	77481063	510	24	500
	96	3'-5'	40453287	40453653	40453920	79167283	267	32	257
	97	3'-5'	44552532	44552737	44553165	83266528	428	27	418
	98	3'-5'	51108630	51108895	51109263	89822626	368	32	358
	99	3'-5'	51429893	51430205	51430526	90143889	321	29	311
	100	3'-5'	51594917	51595008	51595550	90308913	542	15	532
	101	3'-5'	52058038	52058136	52058671	90772034	535	27	525
	102	3'-5'	53098984	53099244	53099617	91812980	373	12	363
	103	3'-5'	55944076	55944325	55944709	94658072	384	25	374
	104	3'-5'	56238447	56238820	56239080	94952443	260	28	250
	105	3'-5'	57284887	57285109	57285520	95998883	411	32	401
	106	3'-5'	63263519	63263596	63264152	101977515	556	23	546
16			NT_039624.7 (1)						
	0	5'-3'	4078710	4078710	4079030	7078710	320	19	310
	1	5'-3'	6938844	6938844	6939223	9938844	379	13	369
	2	5'-3'	7199911	7199911	7200331	10199911	420	10	410
	3	5'-3'	7213422	7213422	7213859	10213422	437	24	427
	4	5'-3'	11923585	11923585	11923975	14923585	390	9	380
	5	5'-3'	11989652	11989652	11990047	14989652	395	9	385
	6	5'-3'	12689763	12689763	12690140	15689763	377	17	367
	7	5'-3'	12958050	12958050	12958374	15958050	324	20	314
	8	5'-3'	13497728	13497728	13498199	16497728	471	24	461
	9	5'-3'	17082771	17082771	17083056	20082771	285	9	275
	10	5'-3'	17691747	17691747	17692090	20691747	343	18	333
	11	5'-3'	32006667	32006667	32007111	35006667	444	12	434

12	5'-3'	32586487	32586487	32586876	35586487	389	26	379
13	5'-3'	34334856	34334856	34335419	37334856	563	24	553
14	5'-3'	41976537	41976537	41976999	44976537	462	9	452
15	5'-3'	46647069	46647069	46647438	49647069	369	30	359
16	5'-3'	48772827	48772827	48773347	51772827	520	31	510
17	5'-3'	49737797	49737797	49738187	52737797	390	25	380
18	5'-3'	51288056	51288056	51288444	54288056	388	20	378
19	5'-3'	51633347	51633347	51633775	54633347	428	31	418
20	5'-3'	52126008	52126008	52126387	55126008	379	8	369
21	5'-3'	53262772	53262772	53263118	56262772	346	20	336
22	5'-3'	59915111	59915111	59915343	62915111	232	25	222
23	5'-3'	61256422	61256422	61256766	64256422	344	30	334
24	5'-3'	61792644	61792644	61793017	64792644	373	25	363
25	3'-5'	127732	127851	128365	3127732	514	32	504
26	3'-5'	2272249	2272539	2272882	5272249	343	31	333
27	3'-5'	5752131	5752332	5752764	8752131	432	12	422
28	3'-5'	8643739	8644014	8644372	11643739	358	34	348
29	3'-5'	14782326	14782443	14782959	17782326	516	28	506
30	3'-5'	18913164	18913247	18913797	21913164	550	15	540
31	3'-5'	21870927	21871140	21871560	24870927	420	28	410
32	3'-5'	23977688	23977955	23978321	26977688	366	25	356
33	3'-5'	32053863	32054164	32054496	35053863	332	26	322
34	3'-5'	33187161	33187401	33187794	36187161	393	32	383
35	3'-5'	33309147	33309396	33309780	36309147	384	20	374
36	3'-5'	34282362	34282712	34282995	37282362	283	12	273
37	3'-5'	36155269	36155456	36155902	39155269	446	22	436
38	3'-5'	36295355	36295616	36295988	39295355	372	34	362
39	3'-5'	36448409	36448709	36449042	39448409	333	19	323
40	3'-5'	36822309	36822565	36822942	39822309	377	31	367
41	3'-5'	37038189	37038312	37038822	40038189	510	7	500
42	3'-5'	37455466	37455725	37456099	40455466	374	9	364
43	3'-5'	37467836	37468062	37468469	40467836	407	16	397
44	3'-5'	38066754	38066873	38067387	41066754	514	10	504
45	3'-5'	38337850	38337965	38338483	41337850	518	24	508
46	3'-5'	41803240	41803404	41803873	44803240	469	22	459
47	3'-5'	42824665	42824859	42825298	45824665	439	30	429
48	3'-5'	42929118	42929390	42929751	45929118	361	20	351

49	3'-5'	45423153	45423630	45423786	48423153	156	25	146
50	3'-5'	45890875	45891264	45891508	48890875	244	32	234
51	3'-5'	50418415	50418581	50419048	53418415	467	17	457
52	3'-5'	51021843	51022113	51022476	54021843	363	16	353
53	3'-5'	53425640	53425868	53426273	56425640	405	31	395
54	3'-5'	54229479	54229774	54230112	57229479	338	18	328
55	3'-5'	55073593	55073775	55074226	58073593	451	14	441
56	3'-5'	57452664	57452974	57453297	60452664	323	20	313
57	3'-5'	57861480	57861737	57862113	60861480	376	30	366
58	3'-5'	60056006	60056507	60056639	63056006	132	33	122
59	3'-5'	60087070	60087328	60087703	63087070	375	15	365
60	3'-5'	60254731	60254817	60255364	63254731	547	18	537
61	3'-5'	60563576	60563821	60564209	63563576	388	6	378
		NT_039625.7 (2)						
62	5'-3'	919203	919203	919617	66214126	414	18	404
63	5'-3'	4112788	4112788	4113162	69407711	374	26	364
64	5'-3'	5095712	5095712	5096070	70390635	358	25	348
65	5'-3'	5270209	5270209	5270516	70565132	307	19	297
66	5'-3'	8224120	8224120	8224500	73519043	380	17	370
67	5'-3'	8995064	8995064	8995458	74289987	394	8	384
68	5'-3'	9751689	9751689	9752137	75046612	448	19	438
69	5'-3'	10307058	10307058	10307417	75601981	359	20	349
70	5'-3'	11878167	11878167	11878573	77173090	406	17	396
71	5'-3'	16611379	16611379	16611788	81906302	409	17	399
72	5'-3'	17009479	17009479	17009869	82304402	390	14	380
73	5'-3'	17137070	17137070	17137667	82431993	597	14	587
74	5'-3'	17301767	17301767	17302153	82596690	386	30	376
75	5'-3'	22148072	22148072	22148544	87442995	472	24	462
76	5'-3'	27068921	27068921	27069367	92363844	446	14	436
77	5'-3'	29608046	29608046	29608444	94902969	398	31	388
78	3'-5'	340697	340977	341330	65635620	353	9	343
79	3'-5'	449370	449722	450003	65744293	281	32	271
80	3'-5'	1014415	1014655	1015048	66309338	393	16	383
81	3'-5'	2477189	2477267	2477822	67772112	555	8	545
82	3'-5'	2699015	2699230	2699648	67993938	418	29	408
83	3'-5'	3461191	3461409	3461824	68756114	415	22	405

	84	3'-5'	3605017	3605314	3605650	68899940	336	21	326
	85	3'-5'	3628497	3628838	3629130	68923420	292	28	282
	86	3'-5'	3764166	3764400	3764799	69059089	399	25	389
	87	3'-5'	4093218	4093460	4093851	69388141	391	27	381
	88	3'-5'	4367368	4367631	4368001	69662291	370	23	360
	89	3'-5'	5937004	5937247	5937637	71231927	390	19	380
	90	3'-5'	7281296	7281637	7281929	72576219	292	21	282
	91	3'-5'	8229609	8229848	8230242	73524532	394	31	384
	92	3'-5'	11424399	11424608	11425032	76719322	424	14	414
	93	3'-5'	16634734	16635118	16635367	81929657	249	15	239
	94	3'-5'	17615834	17616090	17616467	82910757	377	14	367
	95	3'-5'	17892169	17892472	17892802	83187092	330	21	320
	96	3'-5'	19433660	19433902	19434293	84728583	391	33	381
	97	3'-5'	29324346	29324553	29324979	94619269	426	23	416
	98	3'-5'	31048167	31048347	31048800	96343090	453	22	443
	99	3'-5'	31310368	31310499	31311001	96605291	502	25	492
	100	3'-5'	32926962	32926984	32927595	98221885	611	22	601
17			NT_039636.7 (1)						
	0	5'-3'	889265	889265	889664	3889265	399	28	389
	1	5'-3'	2459086	2459086	2459630	5459086	544	9	534
	2	5'-3'	3672015	3672015	3672399	6672015	384	9	374
	3	5'-3'	3877217	3877217	3877601	6877217	384	9	374
	4	3'-5'	917189	917304	917822	3917189	518	14	508
	5	3'-5'	2258829	2259038	2259462	5258829	424	17	414
	6	3'-5'	3040365	3040543	3040998	6040365	455	28	445
	7	3'-5'	3411048	3411316	3411681	6411048	365	9	355
	8	3'-5'	3582268	3582489	3582901	6582268	412	9	402
	9	3'-5'	3788442	3788663	3789075	6788442	412	9	402
	10	3'-5'	4571465	4571865	4572098	7571465	233	12	223
			NT_039638.7 (2)						
	11	5'-3'	5128594	5128594	5128985	12842534	391	21	381
	12	3'-5'	3643766	3643863	3644399	11357706	536	16	526
	13	3'-5'	5591863	5592274	5592496	13305803	222	12	212
	14	3'-5'	5870390	5870802	5871023	13584330	221	12	211

		NT_039649.7 (3)						
15	5'-3'	1986818	1986818	1987378	15793032	560	19	550
16	5'-3'	3315379	3315379	3315749	17121593	370	29	360
17	5'-3'	5990493	5990493	5990860	19796707	367	23	357
18	5'-3'	6103462	6103462	6103822	19909676	360	23	350
19	5'-3'	16753610	16753610	16754114	30559824	504	26	494
20	5'-3'	23098356	23098356	23098756	36904570	400	20	390
21	5'-3'	25554503	25554503	25554857	39360717	354	29	344
22	5'-3'	25976995	25976995	25977365	39783209	370	12	360
23	5'-3'	26260856	26260856	26261210	40067070	354	29	344
24	5'-3'	26925778	26925778	26926200	40731992	422	31	412
25	5'-3'	27513768	27513768	27514095	41319982	327	22	317
26	5'-3'	28855104	28855104	28855492	42661318	388	10	378
27	5'-3'	35854888	35854888	35855462	49661102	574	29	564
28	5'-3'	36594640	36594640	36595108	50400854	468	22	458
29	5'-3'	37103109	37103109	37103643	50909323	534	31	524
30	5'-3'	37114778	37114778	37115162	50920992	384	14	374
31	5'-3'	40397064	40397064	40397436	54203278	372	7	362
32	5'-3'	43796317	43796317	43796722	57602531	405	11	395
33	5'-3'	44469457	44469457	44469776	58275671	319	26	309
34	5'-3'	46497727	46497727	46498104	60303941	377	17	367
35	5'-3'	46962011	46962011	46962400	60768225	389	19	379
36	5'-3'	48208195	48208195	48208587	62014409	392	28	382
37	5'-3'	52183242	52183242	52183789	65989456	547	25	537
38	5'-3'	59930841	59930841	59931166	73737055	325	8	315
39	5'-3'	60545437	60545437	60545937	74351651	500	29	490
40	5'-3'	61821024	61821024	61821440	75627238	416	7	406
41	5'-3'	65516879	65516879	65517031	79323093	152	22	142
42	5'-3'	66501378	66501378	66501726	80307592	348	27	338
43	5'-3'	66501396	66501396	66501726	80307610	330	9	320
44	5'-3'	68136682	68136682	68137046	81942896	364	16	354
45	5'-3'	68500290	68500290	68500634	82306504	344	18	334
46	5'-3'	69674341	69674341	69674718	83480555	377	23	367
47	5'-3'	70875335	70875335	70875597	84681549	262	16	252
48	5'-3'	71322767	71322767	71323157	85128981	390	30	380
49	5'-3'	72002535	72002535	72002850	85808749	315	15	305
50	5'-3'	74388815	74388815	74389353	88195029	538	7	528

51	5'-3'	76335165	76335165	76335455	90141379	290	7	280
52	5'-3'	77814664	77814664	77814971	91620878	307	7	297
53	5'-3'	79802034	79802034	79802369	93608248	335	6	325
54	3'-5'	644334	644600	644967	14450548	367	33	357
55	3'-5'	2312989	2313109	2313622	16119203	513	16	503
56	3'-5'	2371241	2371539	2371874	16177455	335	24	325
57	3'-5'	2935392	2935600	2936025	16741606	425	9	415
58	3'-5'	5390015	5390239	5390648	19196229	409	26	399
59	3'-5'	6006555	6006827	6007188	19812769	361	28	351
60	3'-5'	6458314	6458588	6458947	20264528	359	27	349
61	3'-5'	6666242	6666430	6666875	20472456	445	10	435
62	3'-5'	6780451	6780708	6781084	20586665	376	18	366
63	3'-5'	15322973	15323227	15323606	29129187	379	31	369
64	3'-5'	19388632	19388973	19389265	33194846	292	29	282
65	3'-5'	25838120	25838387	25838753	39644334	366	33	356
66	3'-5'	25955809	25956097	25956442	39762023	345	32	335
67	3'-5'	26650873	26651249	26651506	40457087	257	17	247
68	3'-5'	30915022	30915410	30915655	44721236	245	16	235
69	3'-5'	31003496	31003857	31004129	44809710	272	12	262
70	3'-5'	31774286	31774546	31774919	45580500	373	9	363
71	3'-5'	37616529	37616858	37617162	51422743	304	13	294
72	3'-5'	46211033	46211346	46211666	60017247	320	28	310
73	3'-5'	46447152	46447419	46447785	60253366	366	8	356
74	3'-5'	47105060	47105424	47105693	60911274	269	31	259
75	3'-5'	47490408	47490595	47491041	61296622	446	29	436
76	3'-5'	47592240	47592556	47592873	61398454	317	29	307
77	3'-5'	47787587	47787809	47788220	61593801	411	18	401
78	3'-5'	52352212	52352533	52352845	66158426	312	13	302
79	3'-5'	57287014	57287292	57287647	71093228	355	19	345
80	3'-5'	59659137	59659469	59659770	73465351	301	22	291
81	3'-5'	63010883	63011036	63011516	76817097	480	25	470
82	3'-5'	63341807	63341998	63342440	77148021	442	32	432
83	3'-5'	71735064	71735508	71735697	85541278	189	15	179
84	3'-5'	74263628	74263950	74264261	88069842	311	18	301
85	3'-5'	75967793	75968021	75968426	89774007	405	10	395
86	3'-5'	77383019	77383250	77383652	91189233	402	12	392
87	3'-5'	77792938	77793081	77793571	91599152	490	28	480

	88	3'-5'	78669222	78669534	78669855	92475436	321	11	311
	89	3'-5'	79251529	79251828	79252162	93057743	334	10	324
	90	3'-5'	80505398	80505607	80506031	94311612	424	33	414
18			NT_039674.7 (1)						
	0	5'-3'	519154	519154	519555	3519154	401	31	391
	1	5'-3'	12581218	12581218	12581750	15581218	532	31	522
	2	5'-3'	15579038	15579038	15579508	18579038	470	25	460
	3	5'-3'	16831220	16831220	16831569	19831220	349	6	339
	4	5'-3'	18463849	18463849	18464204	21463849	355	29	345
	5	5'-3'	19789227	19789227	19789595	22789227	368	21	358
	6	5'-3'	23786673	23786673	23787144	26786673	471	7	461
	7	5'-3'	26766413	26766413	26766749	29766413	336	22	326
	8	5'-3'	26990227	26990227	26990548	29990227	321	29	311
	9	5'-3'	30067197	30067197	30067605	33067197	408	23	398
	10	5'-3'	31636297	31636297	31636553	34636297	256	20	246
	11	5'-3'	32108239	32108239	32108640	35108239	401	26	391
	12	5'-3'	32735465	32735465	32735830	35735465	365	30	355
	13	5'-3'	33905526	33905526	33905947	36905526	421	13	411
	14	5'-3'	37373433	37373433	37373909	40373433	476	21	466
	15	5'-3'	39923282	39923282	39923597	42923282	315	14	305
	16	5'-3'	42383242	42383242	42383628	45383242	386	27	376
	17	5'-3'	44039351	44039351	44039660	47039351	309	18	299
	18	5'-3'	44504532	44504532	44504834	47504532	302	21	292
	19	5'-3'	45198838	45198838	45199019	48198838	181	31	171
	20	5'-3'	45576504	45576504	45576858	48576504	354	9	344
	21	5'-3'	46181364	46181364	46181512	49181364	148	12	138
	22	5'-3'	47889766	47889766	47890074	50889766	308	16	298
	23	5'-3'	49096790	49096790	49097129	52096790	339	15	329
	24	5'-3'	52776986	52776986	52777285	55776986	299	9	289
	25	5'-3'	53643931	53643931	53644214	56643931	283	28	273
	26	5'-3'	57066812	57066812	57067014	60066812	202	17	192
	27	5'-3'	57124505	57124505	57124850	60124505	345	8	335
	28	5'-3'	57464131	57464131	57464392	60464131	261	9	251
	29	5'-3'	61946246	61946246	61946636	64946246	390	21	380
	30	5'-3'	61946304	61946304	61946636	64946304	332	21	322
	31	5'-3'	61946358	61946358	61946794	64946358	436	8	426

32	5'-3'	67411291	67411291	67411693	70411291	402	23	392
33	5'-3'	67551569	67551569	67551883	70551569	314	26	304
34	5'-3'	68861094	68861094	68861485	71861094	391	14	381
35	5'-3'	69442999	69442999	69443473	72442999	474	14	464
36	3'-5'	981764	982162	982397	3981764	235	12	225
37	3'-5'	1167529	1167674	1168162	4167529	488	6	478
38	3'-5'	2118989	2119266	2119622	5118989	356	9	346
39	3'-5'	2949978	2950209	2950611	5949978	402	21	392
40	3'-5'	5232266	5232356	5232899	8232266	543	30	533
41	3'-5'	7449036	7449227	7449669	10449036	442	21	432
42	3'-5'	10510420	10510770	10511053	13510420	283	22	273
43	3'-5'	15067848	15068102	15068481	18067848	379	33	369
44	3'-5'	16182934	16183248	16183567	19182934	319	18	309
45	3'-5'	16831025	16831280	16831658	19831025	378	16	368
46	3'-5'	18937978	18938224	18938611	21937978	387	27	377
47	3'-5'	21213766	21214018	21214399	24213766	381	7	371
48	3'-5'	24227283	24227759	24227916	27227283	157	21	147
49	3'-5'	24290570	24290787	24291203	27290570	416	19	406
50	3'-5'	24512075	24512270	24512708	27512075	438	23	428
51	3'-5'	25118564	25118868	25119197	28118564	329	32	319
52	3'-5'	30049229	30049478	30049862	33049229	384	21	374
53	3'-5'	37249704	37250158	37250337	40249704	179	19	169
54	3'-5'	38696843	38697090	38697476	41696843	386	24	376
55	3'-5'	41284467	41284758	41285100	44284467	342	30	332
56	3'-5'	44975636	44975796	44976269	47975636	473	7	463
57	3'-5'	45007457	45007720	45008090	48007457	370	9	360
58	3'-5'	49808811	49809106	49809444	52808811	338	9	328
59	3'-5'	51237637	51237648	51238270	54237637	622	23	612
60	3'-5'	52713604	52713704	52714237	55713604	533	19	523
61	3'-5'	53497180	53497499	53497813	56497180	314	34	304
63	3'-5'	59891808	59892174	59892441	62891808	267	34	257
64	3'-5'	60483801	60484236	60484434	63483801	198	25	188
65	3'-5'	60731041	60731199	60731674	63731041	475	24	465
66	3'-5'	65323168	65323570	65323801	68323168	231	7	221
67	3'-5'	65950022	65950281	65950655	68950022	374	9	364
68	3'-5'	69610654	69610935	69611287	72610654	352	15	342
69	3'-5'	69620994	69621246	69621627	72620994	381	28	371

	70	3'-5'	70115556	70115811	70116189	73115556	378	28	368
	71	3'-5'	70454224	70454409	70454857	73454224	448	23	438
	72	3'-5'	73043090	73043306	73043723	76043090	417	23	407
			NT_039675.7 (2)						
	75	5'-3'	536229	536229	536635	77305377	406	20	396
	76	5'-3'	620445	620445	620838	77389593	393	26	383
	77	3'-5'	124228	124353	124861	76893376	508	11	498
	78	3'-5'	949728	950013	950361	77718876	348	30	338
			NT_039676.7 (3)						
	79	5'-3'	716383	716383	716860	78846001	477	31	467
	80	5'-3'	1835169	1835169	1835776	79964787	607	9	597
	81	5'-3'	3219520	3219520	3219942	81349138	422	20	412
	82	5'-3'	3829911	3829911	3830491	81959529	580	7	570
	83	5'-3'	4211748	4211748	4212092	82341366	344	17	334
			NT_039678.7 (4)						
	84	5'-3'	199961	199961	200252	82590332	291	27	281
	85	5'-3'	2000888	2000888	2001049	84391259	161	23	151
	86	5'-3'	6375039	6375039	6375399	88765410	360	9	350
	87	5'-3'	7008168	7008168	7008478	89398539	310	15	300
	88	3'-5'	368630	368662	369263	82759001	601	29	591
	89	3'-5'	605154	605416	605787	82995525	371	22	361
	90	3'-5'	1278384	1278674	1279017	83668755	343	22	333
	91	3'-5'	2751690	2752066	2752323	85142061	257	20	247
	92	3'-5'	3551166	3551417	3551799	85941537	382	16	372
19			NT_082868.6 (1)						
	0	5'-3'	805940	805940	806545	3805940	605	21	595
	1	5'-3'	1728528	1728528	1728990	4728528	462	11	452
			NT_039687.7 (2)						
	2	5'-3'	1316747	1316747	1317149	8204852	402	14	392
	3	5'-3'	1587356	1587356	1587683	8475461	327	14	317
	4	5'-3'	2289023	2289023	2289411	9177128	388	21	378
	5	5'-3'	8361377	8361377	8361670	15249482	293	26	283

6	5'-3'	8421454	8421454	8421847	15309559	393	24	383
7	5'-3'	8948262	8948262	8948851	15836367	589	29	579
8	5'-3'	10046554	10046554	10046968	16934659	414	30	404
9	5'-3'	12043524	12043524	12043905	18931629	381	21	371
10	5'-3'	12183248	12183248	12183708	19071353	460	11	450
11	5'-3'	14261693	14261693	14262288	21149798	595	21	585
12	5'-3'	20431434	20431434	20431889	27319539	455	8	445
13	5'-3'	22138970	22138970	22139334	29027075	364	25	354
14	5'-3'	22226262	22226262	22226655	29114367	393	13	383
15	5'-3'	22358670	22358670	22359130	29246775	460	13	450
16	5'-3'	23473908	23473908	23474331	30362013	423	27	413
17	5'-3'	29354220	29354220	29354572	36242325	352	20	342
18	5'-3'	32182303	32182303	32182700	39070408	397	17	387
19	5'-3'	32950061	32950061	32950440	39838166	379	11	369
20	5'-3'	33025642	33025642	33026001	39913747	359	11	349
21	5'-3'	44239069	44239069	44239462	51127174	393	22	383
22	5'-3'	44679525	44679525	44680060	51567630	535	9	525
23	5'-3'	44831927	44831927	44832237	51720032	310	27	300
24	5'-3'	44979060	44979060	44979419	51867165	359	19	349
25	5'-3'	45305272	45305272	45305632	52193377	360	8	350
26	5'-3'	47937150	47937150	47937667	54825255	517	26	507
27	5'-3'	52285740	52285740	52286172	59173845	432	13	422
28	3'-5'	1157165	1157450	1157798	8045270	348	27	338
29	3'-5'	1453782	1454067	1454415	8341887	348	27	338
30	3'-5'	2603938	2604304	2604571	9492043	267	32	257
31	3'-5'	4744190	4744368	4744823	11632295	455	10	445
32	3'-5'	8312707	8312853	8313340	15200812	487	18	477
33	3'-5'	11513066	11513354	11513699	18401171	345	10	335
34	3'-5'	12519817	12520273	12520450	19407922	177	13	167
35	3'-5'	13396979	13397221	13397612	20285084	391	23	381
36	3'-5'	13505617	13505739	13506250	20393722	511	9	501
37	3'-5'	22184764	22185000	22185397	29072869	397	30	387
38	3'-5'	22374632	22374831	22375265	29262737	434	20	424
39	3'-5'	23596240	23596466	23596873	30484345	407	13	397
40	3'-5'	24669767	24670021	24670400	31557872	379	30	369
41	3'-5'	28478126	28478264	28478759	35366231	495	11	485
42	3'-5'	30265859	30266109	30266492	37153964	383	21	373

	43	3'-5'	31451235	31451525	31451868	38339340	343	24	333
	44	3'-5'	34641913	34642118	34642546	41530018	428	28	418
	45	3'-5'	36575738	36575973	36576371	43463843	398	28	388
	46	3'-5'	40008728	40008970	40009361	46896833	391	26	381
	47	3'-5'	43492330	43492550	43492963	50380435	413	31	403
	48	3'-5'	46542243	46542363	46542876	53430348	513	34	503
	49	3'-5'	46805014	46805284	46805647	53693119	363	23	353
	50	3'-5'	47846077	47846289	47846710	54734182	421	22	411
	51	3'-5'	49765324	49765713	49765957	56653429	244	26	234
	52	3'-5'	50772593	50772641	50773226	57660698	585	12	575
	53	3'-5'	51167568	51167769	51168201	58055673	432	13	422
	54	3'-5'	53614645	53614924	53615278	60502750	354	32	344
	55	3'-5'	53710545	53710808	53711178	60598650	370	6	360
	56	3'-5'	53949138	53949349	53949771	60837243	422	27	412
X			NT_ 039699.7 (1)						
	0	5'-3'	1677869	1677869	1678248	4677869	379	22	369
	1	5'-3'	1823491	1823491	1823872	4823491	381	22	371
	2	3'-5'	158649	158901	159282	3158649	381	22	371
	3	3'-5'	3016343	3016711	3016976	6016343	265	19	255
	4	3'-5'	3042014	3042280	3042647	6042014	367	15	357
			NT_039700.7 (2)						
	5	5'-3'	5117018	5117018	5117364	11526112	346	25	336
	6	5'-3'	6013899	6013899	6014443	12422993	544	20	534
	7	5'-3'	11443344	11443344	11443703	17852438	359	9	349
	8	3'-5'	2617313	2617581	2617946	9026407	365	26	355
	9	3'-5'	3858249	3858479	3858882	10267343	403	15	393
	10	3'-5'	4289675	4289800	4290308	10698769	508	6	498
	11	3'-5'	6074868	6075074	6075501	12483962	427	14	417
	12	3'-5'	6319309	6319544	6319942	12728403	398	19	388
	13	3'-5'	8391359	8391614	8391992	14800453	378	15	368
	14	3'-5'	9724044	9724244	9724677	16133138	433	30	423
	15	3'-5'	11391843	11392102	11392476	17800937	374	29	364
	16	3'-5'	12721576	12721981	12722209	19130670	228	14	218
	17	3'-5'	14684810	14685139	14685443	21093904	304	27	294

		NT_111909.2 (11)						
18	5'-3'	1928244	1928244	1928620	34232725	376	12	366
		NT_039702.5 (12)						
19	5'-3'	1113985	1113985	1114318	36281826	333	6	323
20	5'-3'	3186849	3186849	3187163	38354690	314	17	304
21	5'-3'	4099556	4099556	4099862	39267397	306	22	296
22	5'-3'	4217898	4217898	4218299	39385739	401	7	391
23	5'-3'	6411738	6411738	6412120	41579579	382	21	372
24	5'-3'	9052208	9052208	9052529	44220049	321	11	311
25	5'-3'	13416039	13416039	13416514	48583880	475	17	465
26	5'-3'	13903071	13903071	13903434	49070912	363	19	353
27	5'-3'	14515738	14515738	14516172	49683579	434	29	424
28	3'-5'	3742306	3742507	3742939	38910147	432	28	422
29	3'-5'	5114946	5115281	5115579	40282787	298	19	288
30	3'-5'	5214399	5214720	5215032	40382240	312	21	302
31	3'-5'	5470135	5470381	5470768	40637976	387	11	377
32	3'-5'	5784923	5785160	5785556	40952764	396	9	386
33	3'-5'	7725509	7725768	7726142	42893350	374	12	364
34	3'-5'	7938034	7938249	7938667	43105875	418	27	408
35	3'-5'	10525242	10525362	10525875	45693083	513	23	503
36	3'-5'	13050177	13050446	13050810	48218018	364	9	354
37	3'-5'	13543780	13544040	13544413	48711621	373	25	363
38	3'-5'	14650095	14650383	14650728	49817936	345	23	335
		NT_039706.7 (15)						
39	5'-3'	999216	999216	999618	53415109	402	20	392
40	5'-3'	1453798	1453798	1454188	53869691	390	27	380
41	5'-3'	3796377	3796377	3796739	56212270	362	29	352
42	5'-3'	5625380	5625380	5625790	58041273	410	15	400
43	5'-3'	6469565	6469565	6469772	58885458	207	13	197
44	5'-3'	8316918	8316918	8317281	60732811	363	7	353
45	5'-3'	13482256	13482256	13482738	65898149	482	8	472
46	5'-3'	17267034	17267034	17267398	69682927	364	30	354
47	5'-3'	20248862	20248862	20249236	72664755	374	12	364
48	5'-3'	20360479	20360479	20360887	72776372	408	8	398
49	5'-3'	22189567	22189567	22189952	74605460	385	25	375

50	5'-3'	23794193	23794193	23794539	76210086	346	20	336
51	5'-3'	24250399	24250399	24250723	76666292	324	22	314
52	5'-3'	26453296	26453296	26453696	78869189	400	21	390
53	5'-3'	28684474	28684474	28684873	81100367	399	24	389
54	5'-3'	31471262	31471262	31471462	83887155	200	9	190
55	5'-3'	32489065	32489065	32489357	84904958	292	9	282
56	5'-3'	37690085	37690085	37690330	90105978	245	30	235
57	5'-3'	38234249	38234249	38234613	90650142	364	25	354
58	5'-3'	38940410	38940410	38940792	91356303	382	28	372
59	5'-3'	39025620	39025620	39026090	91441513	470	31	460
60	5'-3'	47439566	47439566	47440076	99855459	510	23	500
61	5'-3'	52930917	52930917	52931338	105346810	421	6	411
62	5'-3'	53842489	53842489	53842871	106258382	382	31	372
63	3'-5'	1079421	1079651	1080054	53495314	403	20	393
64	3'-5'	1778100	1778375	1778733	54193993	358	9	348
65	3'-5'	2320548	2320627	2321181	54736441	554	8	544
66	3'-5'	3514474	3514690	3515107	55930367	417	31	407
67	3'-5'	3811521	3811902	3812154	56227414	252	20	242
68	3'-5'	4014327	4014793	4014960	56430220	167	25	157
69	3'-5'	5077440	5077718	5078073	57493333	355	29	345
70	3'-5'	6786081	6786349	6786714	59201974	365	17	355
71	3'-5'	9039865	9040108	9040498	61455758	390	14	380
72	3'-5'	12649725	12650112	12650358	65065618	246	28	236
73	3'-5'	16053531	16053691	16054164	68469424	473	13	463
74	3'-5'	19885652	19885878	19886285	72301545	407	24	397
75	3'-5'	20274446	20274509	20275079	72690339	570	22	560
76	3'-5'	20303759	20303940	20304392	72719652	452	22	442
77	3'-5'	20930476	20930703	20931109	73346369	406	10	396
78	3'-5'	22789265	22789446	22789898	75205158	452	22	442
79	3'-5'	25401956	25402238	25402589	77817849	351	12	341
80	3'-5'	26225498	26225936	26226131	78641391	195	25	185
81	3'-5'	27634585	27634837	27635218	80050478	381	19	371
82	3'-5'	28063270	28063696	28063903	80479163	207	17	197
83	3'-5'	28867448	28867721	28868081	81283341	360	15	350
84	3'-5'	29303402	29303573	29304035	81719295	462	15	452
85	3'-5'	30534082	30534345	30534715	82949975	370	7	360
86	3'-5'	30774574	30774692	30775207	83190467	515	8	505

87	3'-5'	31924276	31924525	31924909	84340169	384	12	374
88	3'-5'	32045082	32045346	32045715	84460975	369	24	359
89	3'-5'	32250067	32250251	32250700	84665960	449	23	439
90	3'-5'	32331523	32331744	32332156	84747416	412	18	402
91	3'-5'	34302861	34302936	34303494	86718754	558	32	548
92	3'-5'	36082651	36082903	36083284	88498544	381	14	371
93	3'-5'	37804520	37804824	37805153	90220413	329	25	319
94	3'-5'	37863630	37863892	37864263	90279523	371	25	361
95	3'-5'	38212696	38212953	38213329	90628589	376	25	366
96	3'-5'	38654616	38654838	38655249	91070509	411	24	401
97	3'-5'	41215588	41215838	41216221	93631481	383	21	373
98	3'-5'	41713036	41713291	41713669	94128929	378	15	368
99	3'-5'	42624280	42624524	42624913	95040173	389	26	379
100	3'-5'	45979482	45979739	45980115	98395375	376	25	366
101	3'-5'	48058918	48059143	48059551	100474811	408	32	398
		NT_039713.7 (16)						
102	5'-3'	2335333	2335333	2335796	110805240	463	21	453
103	5'-3'	5967200	5967200	5967560	114437107	360	26	350
104	5'-3'	6533811	6533811	6534153	115003718	342	12	332
105	5'-3'	7755274	7755274	7755699	116225181	425	18	415
106	5'-3'	10354647	10354647	10355089	118824554	442	25	432
107	3'-5'	661852	662231	662485	109131759	254	18	244
108	3'-5'	1064322	1064609	1064955	109534229	346	6	336
109	3'-5'	1194910	1195256	1195543	109664817	287	18	277
110	3'-5'	1717332	1717600	1717965	110187239	365	23	355
111	3'-5'	4156966	4157231	4157599	112626873	368	34	358
112	3'-5'	4884917	4885180	4885550	113354824	370	8	360
113	3'-5'	5177134	5177355	5177767	113647041	412	13	402
114	3'-5'	5862041	5862357	5862674	114331948	317	6	307
115	3'-5'	6735714	6735987	6736347	115205621	360	28	350
116	3'-5'	9638846	9639118	9639479	118108753	361	18	351
117	3'-5'	11337428	11337717	11338061	119807335	344	28	334
118	3'-5'	11629511	11629916	11630144	120099418	228	27	218
		NT_039716.7 (18)						
119	5'-3'	1192535	1192535	1192971	123628977	436	6	426

120	5'-3'	3577297	3577297	3577726	126013739	429	19	419
121	5'-3'	4317166	4317166	4317528	126753608	362	9	352
122	5'-3'	4473577	4473577	4473984	126910019	407	23	397
123	5'-3'	6163761	6163761	6164190	128600203	429	27	419
124	5'-3'	6832765	6832765	6833344	129269207	579	18	569
125	5'-3'	7114679	7114679	7114887	129551121	208	26	198
126	5'-3'	9092786	9092786	9093090	131529228	304	31	294
127	5'-3'	12118875	12118875	12119386	134555317	511	8	501
128	5'-3'	13307034	13307034	13307407	135743476	373	22	363
129	5'-3'	13312558	13312558	13312919	135749000	361	8	351
130	5'-3'	14178068	14178068	14178256	136614510	188	13	178
131	3'-5'	945204	945352	945837	123381646	485	20	475
132	3'-5'	2458791	2458939	2459424	124895233	485	10	475
133	3'-5'	2490204	2490448	2490837	124926646	389	22	379
134	3'-5'	4492003	4492222	4492636	126928445	414	16	404
135	3'-5'	4505011	4505280	4505644	126941453	364	15	354
136	3'-5'	5678617	5678856	5679250	128115059	394	10	384
137	3'-5'	8170923	8170968	8171556	130607365	588	17	578
138	3'-5'	9171185	9171440	9171818	131607627	378	34	368
139	3'-5'	10054367	10054539	10055000	132490809	461	19	451
140	3'-5'	12449166	12449426	12449799	134885608	373	9	363
141	3'-5'	13716011	13716300	13716644	136152453	344	33	334
142	3'-5'	14076758	14076979	14077391	136513200	412	13	402
143	3'-5'	14642454	14642703	14643087	137078896	384	16	374
		NT_039718.7 (19)						
144	5'-3'	1189884	1189884	1190517	138773955	277	30	267
145	5'-3'	2567326	2567326	2567959	140151397	446	27	436
146	5'-3'	2759554	2759554	2760187	140343625	364	30	354
147	5'-3'	3252779	3252779	3253412	140836850	180	25	170
148	5'-3'	5536278	5536278	5536911	143120349	368	18	358
149	5'-3'	5839358	5839358	5839991	143423429	353	17	343
150	5'-3'	5958618	5958618	5959251	143542689	347	22	337
151	5'-3'	6077435	6077435	6078068	143661506	284	17	274
152	5'-3'	6319340	6319340	6319973	143903411	280	8	270
153	5'-3'	9617063	9617063	9617696	147201134	461	24	451
154	5'-3'	9633167	9633167	9633800	147217238	438	19	428

	155	5'-3'	12614744	12614744	12615377	150198815	353	22	343
	156	5'-3'	16699204	16699204	16699837	154283275	424	31	414
	157	5'-3'	17713255	17713255	17713888	155297326	448	27	438
	158	5'-3'	19503517	19503517	19504150	157087588	168	15	158
	159	5'-3'	24898836	24898836	24899469	162482907	290	16	280
	160	5'-3'	26647592	26647592	26648225	164231663	483	31	473
	161	5'-3'	26746406	26746406	26747039	164330477	390	17	380
	162	5'-3'	27738874	27738874	27739507	165322945	420	27	410
	163	5'-3'	27940764	27940764	27941397	165524835	423	14	413
	164	5'-3'	28769017	28769017	28769650	166353088	421	11	411
	165	3'-5'	351340	351424	351973	137935411	549	9	539
	166	3'-5'	14226937	14227262	14227570	151811008	308	9	298
	167	3'-5'	15736780	15737088	15737413	153320851	325	16	315
	168	3'-5'	15938712	15938944	15939345	153522783	401	8	391
	169	3'-5'	20854488	20854647	20855121	158438559	474	18	464
	170	3'-5'	21678489	21678760	21679122	159262560	362	11	352
	171	3'-5'	21870242	21870427	21870875	159454313	448	26	438
	172	3'-5'	22194809	22195138	22195442	159778880	304	31	294
	173	3'-5'	23101466	23101913	23102099	160685537	186	12	176
	174	3'-5'	24466505	24466731	24467138	162050576	407	34	397
	175	3'-5'	26197615	26197924	26198248	163781686	324	27	314
	176	3'-5'	26998091	26998390	26998724	164582162	334	25	324
	177	3'-5'	27864573	27864779	27865206	165448644	427	22	417
Y			NT_078925.6 (1)						
	0	3'-5'	243539	243860	244172	243539	312	34	302
	1	3'-5'	857240	857444	857873	857240	429	26	419
	2	3'-5'	937740	937944	938373	937740	429	26	419

Supplemental Table S2: Possible spatial correlation between protein-coding genes and pol III transcription units.

chromosome	number of genes	numeber of items
1	1819	190
2	2445	138
3	1579	80
4	1881	136
5	1773	99
6	1906	138
7	2908	141
8	1446	123
9	1640	88
10	1412	152
11	2094	98
12	1369	124
13	1401	80
14	1538	119
15	1091	107
16	973	101
17	1511	90
18	823	90
19	974	57
X	1847	178
Y	53	4
total	32483	2333

Supplemental Table S3: subchromosomal localization of pol III type 3 putative transcription units in mouse chromosomes

chromosome	Number of items	Number of items in genes	% items in genes
1	190	66	34,74
2	138	43	31,16
3	80	22	27,50
4	136	30	22,06
5	99	33	33,33
6	138	43	31,16
7	141	50	35,46
8	123	25	20,33
9	88	40	45,45
10	152	36	23,68
11	98	25	25,51
12	124	24	19,35
13	80	23	28,75
14	119	38	31,93
15	107	38	35,51
16	101	33	32,67
17	90	30	33,33
18	90	18	20,00
19	57	23	40,35
X	178	62	34,83
Y	4	0	0,00
total	2333	702	28,91

Supplemental Table S4: analysis of genes/chromosome length

chr	chr.length (bp)	exons	avg exon (bp)	exons (bp)	introns	avg intron (bp)	introns (bp)	genes (bp)	genes/chr length
1	197195432	13351	284	3791684	11952	5476	65449152	69240836	0,35
2	181748087	16959	294	4985946	15015	4369	65600535	70586481	0,39
3	159599783	9530	299	2849470	8393	5238	43962534	46812004	0,29
4	155630120	12272	290	3558880	10807	4420	47766940	51325820	0,33
5	152537259	13795	278	3835010	12315	4459	54912585	58747595	0,39
6	149517037	10730	286	3068780	9347	5741	53661127	56729907	0,38
7	152524553	15995	302	4830490	13733	3578	49136674	53967164	0,35
8	131738871	10285	279	2869515	9123	4576	41746848	44616363	0,34
9	124076172	11796	282	3326472	10502	4370	45893740	49220212	0,40
10	129993255	9682	289	2798098	8555	5368	45923240	48721338	0,37
11	121843856	15849	275	4358475	14111	3237	45677307	50035782	0,41
12	121257530	7220	296	2137120	6309	5734	36175806	38312926	0,32
13	120284312	7311	306	2237166	6311	5963	37632493	39869659	0,33
14	125194864	8024	286	2294864	7021	6035	42371735	44666599	0,36
15	103494974	8396	279	2342484	7525	4611	34697775	37040259	0,36
16	98319150	6350	297	1885950	5573	5719	31871987	33757937	0,34
17	95272651	9867	271	2673957	8691	3687	32043717	34717674	0,36
18	90772031	5069	344	1743736	4468	7265	32460020	34203756	0,38
19	61424300	7060	270	1906200	6260	3989	24971140	26877340	0,44
X	166650296	7949	312	2480088	6705	5846	39197430	41677518	0,25
Y	15902555	442	242	106964	356	3395	1208620	1315584	0,08
total	2654977088	207932		60081349	183072		872361405	932442754	

Supplemental Table S5: chi square test analysis of the correlation between single polIII transcription units and their correspondent protein-coding host genes

	OBSERVED		EXPECTED		
chromosome	cogenes	items non in genes	cogenes	items non in genes	chi-square
1	66	124	66,71	123,29	0,914
2	43	95	53,60	84,40	0,064
3	22	58	23,46	56,54	0,719
4	30	106	44,85	91,15	0,007
5	33	66	38,13	60,87	0,290
6	43	95	52,36	85,64	0,101
7	50	91	49,89	91,11	0,984
8	25	98	41,66	81,34	0,002
9	40	48	34,91	53,09	0,267
10	36	116	56,97	95,03	0,000
11	25	73	40,24	57,76	0,002
12	24	100	39,18	84,82	0,003
13	23	57	26,52	53,48	0,404
14	38	81	42,46	76,54	0,394
15	38	69	38,29	68,71	0,953
16	33	68	34,68	66,32	0,725
17	30	60	32,80	57,20	0,540
18	18	72	33,91	56,09	0,001
19	23	34	24,94	32,06	0,604
X	62	116	44,52	133,48	0,002
Y	0	4	0,33	3,67	0,548
total	702	1631	819,36	1513,64	3,57614E-07

Supplemental Table S5: chi square test analysis of the correlation between single polIII transcription units and their correspondent protein-coding host genes

	OBSERVED		EXPECTED		
chromosome	cogenes	items non in genes	cogenes	items non in genes	chi-square
1	66	124	66,71	123,29	0,914
2	43	95	53,60	84,40	0,064
3	22	58	23,46	56,54	0,719
4	30	106	44,85	91,15	0,007
5	33	66	38,13	60,87	0,290
6	43	95	52,36	85,64	0,101
7	50	91	49,89	91,11	0,984
8	25	98	41,66	81,34	0,002
9	40	48	34,91	53,09	0,267
10	36	116	56,97	95,03	0,000
11	25	73	40,24	57,76	0,002
12	24	100	39,18	84,82	0,003
13	23	57	26,52	53,48	0,404
14	38	81	42,46	76,54	0,394
15	38	69	38,29	68,71	0,953
16	33	68	34,68	66,32	0,725
17	30	60	32,80	57,20	0,540
18	18	72	33,91	56,09	0,001
19	23	34	24,94	32,06	0,604
X	62	116	44,52	133,48	0,002
Y	0	4	0,33	3,67	0,548
total	702	1631	819,36	1513,64	3,57614E-07

Supplemental Table S6: fraction of intronic and exonic pol III transcription units in single chromosomes

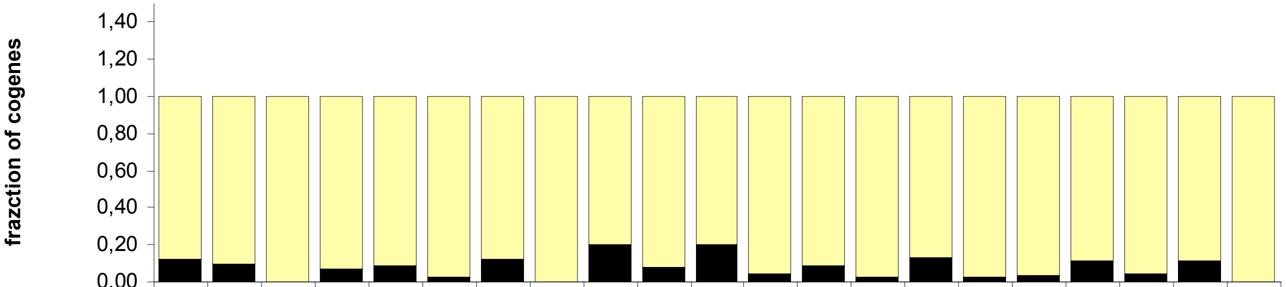

	1	2	3	4	5	6	7	8	9	10	11	12	13	14	15	16	17	18	19	X	Y
in introns	0,88	0,91	1,00	0,93	0,91	0,98	0,88	1,00	0,80	0,92	0,80	0,96	0,91	0,97	0,87	0,97	0,97	0,89	0,96	0,89	1,00
in exons	0,12	0,09	0,00	0,07	0,09	0,02	0,12	0,00	0,20	0,08	0,20	0,04	0,09	0,03	0,13	0,03	0,03	0,11	0,04	0,11	0,00

chromosomes

Supplemental Table S7: chi square test analysis of the correlation between th novel single polIII transcription units and introns

	OBSERVED		EXPECTED		
chromosome	in exons	in introns	in exons	in introns	chi-square
1	8	58	3,61	62,39	0,0177
2	4	39	3,04	39,96	0,5667
3	0	22	1,34	20,66	0,2324
4	2	28	2,08	27,92	0,9541
5	3	30	2,15	30,85	0,5512
6	1	42	2,33	40,67	0,3713
7	6	44	4,48	45,52	0,4501
8	0	25	1,61	23,39	0,1899
9	8	32	2,70	37,30	0,0008
10	3	33	2,07	33,93	0,5041
11	5	20	2,18	22,82	0,0453
12	1	23	1,34	22,66	0,7632
13	2	21	1,29	21,71	0,5204
14	1	37	1,95	36,05	0,4841
15	5	33	2,40	35,60	0,0835
16	1	32	1,84	31,16	0,5225
17	1	29	2,31	27,69	0,3695
18	2	16	0,92	17,08	0,2461
19	1	22	1,63	21,37	0,6081
X	7	55	3,69	58,31	0,0755
Y	0	0	0,00	0,00	
total	61	641	45,23	656,77	0,0154

CHR	Tr. Unit	gene	gene ID	intr/ex	position in chromosome	Length	TATA distance	poly-T distance
1	1	regulator of G-protein signaling 20	Rgs20	I	4.994.160	337	6	327
1	2	hypothetical protein LOC320492		I	11.682.780	263	16	253
1	3	hypothetical protein LOC320492		I	11.745.619	377	13	367
1	4	potassium voltage-gated channel, subfamily Q, member 5	Kcnq5	I	21.841.135	362	26	352
1	5	hypothetical protein LOC76982		I	9.596.021	491	8	481
1	6	centrosome and spindle pole associated protein 1	Cspp1	I	10.061.605	415	6	405
1	7	centrosome and spindle pole associated protein 1	Cspp1	I	10.105.421	588	21	578
1	8	polyductin	Pkhd1	I	20.101.314	491	30	481
1	9	liver regeneration p-53 related protein		I	24.794.644	409	20	399
1	10	similar to AF4/FMR2 family member 3 (Protein LAF-4) (Lymp...		I	38.325.597	137	20	127
1	11	solute carrier family 39 (zinc transporter), member 10	Slc39a10	E	46.892.767	390	18	380
1	12	transmembrane protein with EGF-like and two follistatin-l...	Tmeff1	I	51.231.495	325	7	315
1	13	GPI deacylase	Pgap	I	54.568.849	393	12	383
1	14	aldehyde oxidase 3-like 1	Aox3l1	I	58.339.268	216	8	206
1	15	amyotrophic lateral sclerosis 2 (juvenile) chromosome reg...	Als2cr19	I	59.375.703	416	28	406
1	16	v-erb-a erythroblastic leukemia viral oncogene homolog 4 ...	Erbb4	I	68.776.308	245	17	235
		v-erb-a erythroblastic leukemia viral oncogene homolog 4 ...	Erbb4	I				
1	17	v-erb-a erythroblastic leukemia viral oncogene homolog 4 ...	Erbb4	I	69.023.571	372	28	362
		v-erb-a erythroblastic leukemia viral oncogene homolog 4 ...	Erbb4	I				
1	18	sperm associated antigen 16 isoform 1	Spag16	I	70.608.903	352	16	342
		sperm associated antigen 16 isoform 2	Spag16	I				
1	19	nonhomologous end-joining factor 1	Nhej1	I	75.084.377	533	25	523
1	20	brain-specific angiogenesis inhibitor 3	Bai3	E	25.452.974	435	7	425
1	21	brain-specific angiogenesis inhibitor 3	Bai3	I	25.489.776	387	13	377
1	22	hypothetical protein LOC213056		I	58.587.398	582	20	572
1	23	v-erb-a erythroblastic leukemia viral oncogene homolog 4 ...	Erbb4	I	69.028.557	395	30	385
		v-erb-a erythroblastic leukemia viral oncogene homolog 4 ...	Erbb4	I				
1	24	dedicator of cytokinesis 10 isoform 2	Dock10	I	80.555.966	329	18	319
1	25	hypothetical protein LOC241134		E	81.083.497	404	23	394
1	26	sphingosine kinase type 1-interacting protein	Sphkap	I	83.289.342	335	25	325
1	27	Pid1 phosphotyrosine interaction domain containing 1	Pid1	I	84.087.449	165	17	155
1	28	hypothetical protein		E	99.139.804	354	17	344
1	29	hypothetical protein LOC227446		E	107.622.734	423	16	413
1	30	Yeast Sps1/Ste20-related kinase 4	Ysk4	I	129.745.924	215	26	205

1	31	thrombospondin, type I, domain containing 7B	Thsd7b	I	131.504.896	377	16	367
1	32	thrombospondin, type I, domain containing 7B	Thsd7b	I	131.687.824	399	17	389
1	33	Fam72a family with sequence similarity 72, member A	Fam72a	I	133.434.714	300	16	290
1	34	DBCCR1-like	:CR1-like (Fa	I	148.562.696	386	24	376
1	35	ral guanine nucleotide dissociation stimulator-like 1	Rgl1	I	154.468.463	285	6	275
1	36	armadillo repeat containing 9 isoform 1	Armc9	I	88.150.689	382	17	372
1	37	COP9 (constitutive photomorphogenic) homolog, subunit 7b	Cops7b	I	88.493.445	375	24	365
1	38	secreted nidogen domain protein	Sned1	I	95.175.069	379	33	369
1	39	histidine acid phosphatase domain containing 1	Ppip5K1	I	99.618.530	290	14	280
1	40	contactin associated protein-like 5	Cntnap5a	I	102.352.425	386	34	376
1	41	contactin associated protein-like 5A	Cntnap5a	I	118.410.453	333	6	323
1	42	dipeptidyl peptidase 10	Dpp10	I	125.635.862	160	24	150
1	43	dipeptidyl peptidase 10	Dpp10	I	125.873.211	542	6	532
1	44	thrombospondin, type I, domain containing 7B	Thsd7b	I	131.461.383	397	21	387
1	45	DBCCR1-like	DBCCR1-like	I	148.385.966	259	21	249
1	46	hypothetical protein LOC545370		I	152.828.556	340	19	330
1	47	RIKEN cDNA 1700025G04		I	153.756.996	524	18	514
1	48	astrotactin 1	Astn1	I	160.567.364	322	22	312
1	49	pappalysin 2	Pappa2	I	160.875.378	369	30	359
1	50	kelch-like 20	Klhl20	I	163.024.762	322	8	312
1	51	succinate dehydrogenase complex, subunit C, integral memb...	Sdhc	I	173.062.910	308	31	298
1	52	CD84 antigen	CD84	E	173.782.101	380	10	370
1	53	Fc receptor, IgE, high affinity I, alpha polypeptide	Fcer1a	E	175.155.312	477	9	467
1	54	spectrin alpha 1	Spna1	I	176.167.204	422	23	412
1	55	regulator of G protein signaling 7	Rgs7	I	176.993.722	362	9	352
1	56	phospholipase D family, member 5	Pld5	I	178.046.546	372	9	362
1	57	similar to chromosome 1 open reading frame 101		I	179.842.251	405	7	395
1	58	hypothetical protein LOC78825		I	180.158.370	229	13	219
1	59	SET and MYND domain containing 3	Smyd3	I	181.421.928	388	28	378
1	60	leucine zipper transcription regulator 2	Sec16b	I	159.463.296	400	8	390
1	61	RAB GTPase activating protein 1-like isoform b	Rabgap1l	I	162.258.156	358	15	348
		RAB GTPase activating protein 1-like isoform a	Rabgap1l	I				
1	62	coagulation factor V	F5	E	166.138.907	404	6	394
1	63	Cdc42 binding protein kinase alpha isoform 16	Cdc42bpa	I	181.997.769	395	23	385
		Cdc42 binding protein kinase alpha isoform 14	Cdc42bpa	I				
1	64	usherin	Ush2a	I	190.340.675	324	23	314
1	65	transforming growth factor, beta 2	Tgfb2	I	188.460.286	321	21	311

1	66	usherin	Ush2a	I	190.234.720	405	7	395
1	67	synaptotagmin XIV	Syt14	I	194.850.134	216	24	206
2	68	NOL1/NOP2/Sun domain family 6		I	14963053	254	14	244
2	69	enhancer trap locus 4	Etl4	I	20355591	338	11	328
2	70	USP6 N-terminal like isoform b	Usp6nl	I	6275100	459	28	449
2		USP6 N-terminal like isoform a	Usp6nl	I				
2	71	hypothetical protein		I	7347002	379	34	369
2	72	plexin domain containing 2 precursor	Plxdc2	I	16332982	407	11	397
2	73	hypothetical protein LOC75069		I	16894723	414	28	404
2	74	hypothetical protein LOC75069		I	16896540	378	33	368
2	75	enkurin	Enkur	I	21122616	387	32	377
2	76	spectrin SH3 domain binding protein 1 isoform 1	Abi1	E	22826598	351	26	341
		spectrin SH3 domain binding protein 1 isoform 4	Abi1	I				
2	77	pre B-cell leukemia transcription factor 3	Pbx3	I	34040634	454	27	444
2	78	mitogen-activated protein kinase associated protein 1	Mapkap1	I	34380168	297	21	287
2	79	Scai suppressor of cancer cell invasion	Scai	I	39024408	474	20	464
2	80	nebulin	Neb	I	52071705	322	29	312
2	81	UDP-N-acetyl-alpha-D-galactosamine:polypeptide N-acetylga...	Galnt3	I	54516885	338	12	328
2	82	TGF-beta induced apoptosis protein 2	Csrnp3	I	65855178	265	8	255
2	83	myosin IIIB	Myo3b	I	70201238	385	19	375
2	84	tousled-like kinase 1	Tlk1	E	70555792	250	24	240
2	85	leucine zipper- and sterile alpha motif-containing kinase...	230120H23F	I	72176292	381	9	371
		leucine zipper- and sterile alpha motif-containing kinase...	230120H23F	I				
2	86	mitogen activated protein kinase 8 interacting protein 1	Mapk8ip1	I	92232025	195	22	185
2	87	low density lipoprotein receptor class A domain containing 3	Ldlrad3	I	102006178	333	9	323
2	88	homeodomain interacting protein kinase 3	Hipk3	I	104272391	305	11	295
2	89	kinesin family member 18A	Kif18a	I	109171302	390	17	380
2	90	hypothetical protein LOC399568	BC052040	I	115561155	283	29	273
2	91	cancer susceptibility candidate 5	Casc5	E	118913228	414	15	404
2	92	calcium binding protein P22	500003O03F	I	119382589	374	10	364
2	93	transformation related protein 53 binding protein 1	Trp53bp1	I	121092645	383	15	373
2	94	proprotein convertase subtilisin/kexin type 2	Pcsk2	I	143466704	191	14	181
2	95	D-tyrosyl-tRNA deacylase 1	Dtd1	I	144529152	156	8	146
2	96	protein tyrosine phosphatase, receptor type, T	Ptprt	I	161585121	530	17	520
2	97	solute carrier family 4, sodium bicarbonate cotransporter...	Slc4a11	I	62074716	397	30	387
2	98	sodium channel, voltage-gated, type VII, alpha polypeptide	Scn7a	I	66546166	318	7	308
2	99	sodium channel, voltage-gated, type VII, alpha polypeptide	Scn7a	I	66553896	409	25	399

2	100	zinc finger protein 533	Zfp385b	I	77527272	351	26	341
2	101	hypothetical protein LOC72446		I	101579268	389	11	379
		hypothetical protein LOC72446		I				
2	102	low density lipoprotein receptor class A domain containing 3	Ldlrad3	I	101891909	303	7	293
2	103	yeast INO80-like protein	Ino80	I	119228404	340	16	330
2	104	MAX-interacting protein	Mga	E	119772746	366	15	356
2	105	FERM domain containing 5	Frmd5	I	121522980	404	23	394
2	106	hypothetical protein LOC629499	)22505G16F	I	157092587	352	22	342
2	107	protein tyrosine phosphatase, receptor type, T	Ptprt	I	161657905	487	32	477
2	108	tumor differentially expressed protein 1	Serinc3	I	163461363	395	22	385
2	109	RAB22A, member RAS oncogene family	Rab22a	I	173491893	364	22	354
3	110	zinc finger homeodomain 4	Zfhx4	I	4835075	503	27	493
3	111	hypothetical protein LOC67306	Fam164a	I	5290623	199	24	189
3	112	glucocorticoid induced gene 1	Zfp704	I	9598377	148	29	138
3	113	zinc finger homeodomain 4	Zfhx4	I	5324427	409	22	399
3	114	SIRP beta 1 isoform 3	Sirpb1	I	15418056	133	17	123
		SIRP beta 1 isoform 2	Sirpb1	I				
3	115	hypothetical protein		I	15549665	133	17	123
3	116	transient receptor potential cation channel, subfamily C,...	Trpc4	I	36555000	444	13	434
3	117	hypothetical protein LOC229227		I	36859369	221	25	211
3	118	neuroligin 1	Nlgn1	I	25570499	341	9	331
3	119	hypothetical protein LOC75740 isoform 1		I	29109962	375	7	365
3	120	ATPase, Class VI, type 11B	Atp11b	I	35657037	419	26	409
3	121	fibroblast growth factor 2	Fgf2	I	37254066	613	12	603
3	122	major facilitator superfamily domain containing 8	Mfsd8	I	40634522	387	17	377
3	123	hypothetical protein		I	133526974	151	16	141
3	124	hypothetical protein		I	133547690	593	20	583
3	125	TNNI3 interacting kinase	Tnni3k	I	154467799	449	15	439
3	126	neurotractin isoform b	Negr1	I	156415988	339	10	329
		neurotractin isoform a	Negr1	I				
3	127	glutathione S-transferase, C-terminal domain containing	Gstcd	I	132712062	460	22	450
3	128	hypothetical protein		I	133874908	384	26	374
3	129	hypothetical protein LOC381476	)30007M17F	I	139212689	335	29	325
3	130	phosphatidylinositol glycan anchor biosynthesis, class K ...	Pigk	I	152426656	247	24	237
		phosphatidylinositol glycan anchor biosynthesis, class K ...	Pigk	I				
3	131	hypothetical protein		I	152833836	415	13	405
4	132	thymocyte selection-associated HMG box	Tox	I	6884978	436	10	426

4	133	F-box and leucine-rich repeat protein 4	Fbxl4	I	22322567	381	6	371
4	134	hypothetical protein LOC69984	'00025O08R	I	22767633	360	24	350
4	135	thymocyte selection-associated HMG box	Tox	I	6706981	407	11	397
4	136	hypothetical protein LOC73314		I	14679121	429	20	419
4	137	kelch-like 32	Klhl32	I	24556591	385	27	375
4	138	Eph receptor A7	Epha7	I	28821935	522	19	512
4	139	ATP-binding cassette 1, sub-family A, member 1	Abca1	E	53121735	522	6	512
4	140	polydom precursor	Svep1	I	58153774	417	12	407
4	141	RAD23b homolog	Rad23b	E	55380358	437	26	427
4	142	hypothetical protein LOC214106	933430I17R	I	62195461	384	22	374
4	143	transducin-like enhancer protein 1	Tle1	I	71795825	403	24	393
4	144	FERM domain containing 3	Frmd3	I	73809355	525	27	515
4	145	gene amplified in squamous cell carcinoma 1	Kdm4c	I	73959967	474	7	464
4	146	hypothetical protein		I	77577045	477	9	467
4	147	hypothetical protein		I	81733476	479	24	469
4	148	SH3-domain GRB2-like 2	Sh3gl2	I	84906885	247	29	237
4	149	deleted in bladder cancer chromosome region candidate 1	Dbc1	I	68464077	385	27	375
4	150	protein tyrosine phosphatase, receptor type, D isoform B	Ptprd	I	75741017	390	26	380
		protein tyrosine phosphatase, receptor type, D isoform A						
4	151	hypothetical protein LOC320226	930473A06R	I	83260683	486	12	476
4	152	SH3-domain GRB2-like 2	Sh3gl2	I	84991610	292	14	282
4	153	hypothetical protein		I	85306106	421	34	411
4	154	Fas-associated factor 1	Faf1	I	109422649	391	16	381
4	155	hypothetical protein		I	114175847	412	28	402
4	156	phosphatidic acid phosphatase type 2B	Ppap2b	I	104875938	301	20	291
4	157	endoplasmic reticulum protein ERp19	Txndc12	I	108514992	414	15	404
4	158	hypothetical protein LOC230613		I	112404708	381	9	371
4	159	hypothetical protein		I	125102194	586	15	576
4	160	zinc finger protein 258	Zmym6	I	126797821	403	27	393
4	161	podoplanin	Pdpn	I	142859844	239	27	229
5	162	hypothetical protein LOC231014	330182L06R	I	15566827	371	18	361
5	163	piccolo (presynaptic cytomatrix protein)	Pclo	I	14705456	376	28	366
5	164	semaphorin 3E	Sema3e	I	14200804	416	20	406
5	165	piccolo (presynaptic cytomatrix protein)	Pclo	I	14722143	374	11	364
5	166	membrane associated guanylate kinase, WW and PDZ domain c...	Magi2	I	19538552	390	29	380
5	167	membrane associated guanylate kinase, WW and PDZ domain c...	Magi2	I	19780578	386	23	376
		membrane associated guanylate kinase, WW and PDZ domain c...	Magi2	I				

5	168	F-box and leucine-rich repeat protein 13	FBXL13	E	21120406	387	7	377
5	169	reelin precursor	Reln	E	21426083	392	23	382
5	170	calcium channel, voltage-dependent, alpha2/delta subunit 1	Cacna2d1	I	15556660	415	7	405
5	171	calcium channel, voltage-dependent, alpha2/delta subunit 1	Cacna2d1	I	15566827	371	18	361
5	172	membrane associated guanylate kinase, WW and PDZ domain c...	Magi2	I	18815491	364	13	354
5	173	reelin precursor	Reln	I	21426957	305	21	295
5	174	brain and reproductive organ-expressed protein isoform V	Bre	I	32036303	348	29	338
		brain and reproductive organ-expressed protein isoform I	Bre	I				
5	175	hypothetical protein LOC320661	D5Ertd579e	E	36963847	416	26	406
5	176	LIM domain binding 2 isoform 1	Ldb2	I	45064428	464	7	454
		LIM domain binding 2 isoform 2	Ldb2	I				
5	177	slit homolog 2	Slit2	I	48508580	334	26	324
5	178	potassium channel interacting protein 4	Kcnip4	I 1	49159664	375	30	365
5	179	hypothetical protein LOC319807	I10047P20R	I	64183394	401	17	391
5	180	corin	corin	I	72870725	624	8	614
5	181	Bod1l biorientation of chromosomes in cell division 1-like	Bod1l	I	42224856	331	27	321
5	182	hypothetical protein LOC66768		I	48768546	284	16	274
5	183	potassium channel tetramerisation domain containing 8	Kctd8	I	69602587	525	34	515
5	184	circadian locomoter output cycles kaput	Clock	I	76668944	377	23	367
5	185	hypothetical protein		I	117119419	230	11	220
5	186	density-regulated protein	Denr	I	124370261	340	18	330
5	187	thyroid hormone receptor associated protein 2	Med13l	I	119148011	364	34	354
5	188	hypothetical protein LOC243274		I	128646608	367	34	357
5	189	UDP-GalNAc:polypeptide N-acetylgalactosaminyltransferase-...	Wbscr17	I	131358154	388	22	378
5	190	calneuron 1	Caln1	I	131094713	406	25	396
5	191	autism susceptibility candidate 2	Auts2	I	132247742	375	16	365
5	192	autism susceptibility candidate 2	Auts2	I	132933820	398	30	388
5	193	IQ motif containing E	Iqce	I	141176358	152	26	142
5	194	RNA polymerase 1-3 isoform 2	Polr1d	I	147900661	423	25	413
5	195	PABP-dependent poly(A) nuclease 3	Pan3	I	148340676	377	6	367
5	196	furry homolog	Fryl	I	151115811	384	19	374
6	197	solute carrier family 13 (sodium/sulphate symporters), me...	Slc13a1	I	24103218	366	27	356
6	198	glutamate receptor, metabotropic 7	Grm7	I	27423429	392	30	382
6	199	low density lipoprotein-related protein 12 isoform 2	St7	I	17705603	371	12	361
		low density lipoprotein-related protein 12 isoform 1	St7	I				
6	200	glutamate receptor, metabotropic 7	Grm7	I	28035853	338	24	328

6	201	nuclear respiratory factor 1	Nrf1	I	30084241	438	24	428
6	202	diacylglycerol kinase, iota	Dgki	I	36896027	587	8	577
6	203	Eph receptor A1	Epha1	I	42318344	330	17	320
6	204	contactin associated protein-like 2 isoform a	Cntnap2	I	46324163	359	21	349
6	205	muskelin 1, intracellular mediator containing kelch motifs	Mkln1	I	31436334	184	26	174
6	206	caldesmon 1	Cald1	I	34693736	406	12	396
6	207	homeodomain interacting protein kinase 2	Hipk2	I	38725904	260	13	250
6	208	thiamin pyrophosphokinase	tpk1	I	43561490	372	31	362
6	209	SKAP55 homologue	Skap2	I	51942667	244	26	234
6	210	hect domain and RLD 5 isoform 2	Herc5	I	57602531	409	13	399
		hect domain and RLD 5 isoform 1		I				
6	211	hypothetical protein LOC232035	Fam190a	I	61443245	449	26	439
6	212	catenin (cadherin associated protein), alpha 2 isoform 1	Ctnna2	I	76947234	448	11	438
		catenin (cadherin associated protein), alpha 2 isoform 2		I				
6	213	catenin (cadherin associated protein), alpha 2 isoform 1	Ctnna2	I	77087477	422	25	412
		catenin (cadherin associated protein), alpha 2 isoform 2		I				
6	214	sideroflexin 5	Sfxn5	I	85225402	153	19	143
6	215	TAFA1 protein	Fam19a1	I	96447571	372	14	362
6	216	hypothetical protein		I	105559070	265	21	255
6	217	contactin 4	Cntn4	I	106441937	396	9	386
6	218	inositol 1,4,5-triphosphate receptor 1	Itpr1	I	108490208	149	13	139
6	219	transmembrane and coiled coil domains 1 isoform 2	Tmcc1	I	116032993	413	22	403
6	220	SKAP55 homologue	Skap2	I	51835090	420	32	410
6	221	5'-nucleotidase, cytosolic III	Nt5c3	I	56858159	454	7	444
6	222	synuclein, alpha	Snca	I	60726448	377	9	367
		synuclein, alpha		I				
6	223	hypothetical protein LOC232035	Fam190a	I	61724365	216	32	206
6	224	interleukin 23 receptor	Il23r	I	67385939	382	9	372
6	225	zinc finger protein 638	Zfml	E	83933637	261	11	251
6	226	SEC15-like 2 isoform 1	Exoc6b	I	84845703	371	15	361
		SEC15-like 2 isoform 2		I				
6	227	TAFA1 protein	Fam19a1	I	96208364	346	24	336
6	228	forkhead box P1	Foxp1	I	98944861	474	17	464
6	229	RING1 and YY1 binding protein	Rybp	I	100224064	333	30	323
6	230	glutamate receptor, metabotropic 7	Grm7	I	110745643	316	14	306
6	231	bromodomain containing 1	Brpf1	I	113263834	560	8	550
6	232	calcium/calmodulin-dependent protein kinase I	Camk1	I	113288428	450	27	440

6	233	peroxisome proliferator activated receptor gamma	Pparg	I	115372421	553	14	543
6	234	transmembrane and coiled coil domains 1 isoform 2	Tmcc1	I	116055162	265	18	255
6	235	wingless-related MMTV integration site 5B	Wnt5b	I	119385969	155	7	145
6	236	hypothetical protein LOC74741	5730419I0 9Rik	I	142974314	199	14	189
6	237	phosphodiesterase 3A, cGMP inhibited	Pdea3	I	141333951	502	14	492
6	238	mitochondrial ribosomal protein S35	Mrps35	I	147000322	368	8	358
6	239	FGFR1 oncogene partner 2	Fgfr1op2	I	146538725	377	34	367
7	240	similar to vomeronasal 2, receptor, 15 isoform 3	LOC628326	I	8258601	390	11	380
7		similar to vomeronasal 2, receptor, 15 isoform 2		I				
7	241	similar to vomeronasal 2, receptor, 15 isoform 2	LOC628326	I	8570852	387	11	377
		similar to vomeronasal 2, receptor, 15 isoform 1		I				
7	242	vomeronasal 2, receptor, 14	LOC628326	I	8960473	388	11	378
7	243	similar to vomeronasal 2, receptor, 15 isoform 1	LOC628326	I	9058179	390	11	380
7	244	similar to putative pheromone receptor	LOC628326	I	9213863	388	11	378
7	245	similar to vomeronasal 2, receptor, 15 isoform 1	LOC628326	I	9257906	387	11	377
		similar to vomeronasal 2, receptor, 15 isoform 2		I				
7	246	similar to vomeronasal 2, receptor, 15 isoform 3	LOC628326	I	9364502	390	11	380
		similar to vomeronasal 2, receptor, 15 isoform 2		I				
7	247	similar to vomeronasal 2, receptor, 14 isoform 2	LOC545908	I	9644408	390	11	380
		similar to vomeronasal 2, receptor, 14 isoform 1		I				
7	248	similar to putative pheromone receptor		I	9933134	388	11	378
7	249	similar to vomeronasal 2, receptor, 15 isoform 1	LOC628326	I	9977237	387	11	377
		similar to vomeronasal 2, receptor, 15 isoform 2						
7	250	hypothetical protein LOC232947		I	20138548	260	17	250
7	251	protein tyrosine phosphatase, receptor type, H	Ptprh	I	4551693	203	32	193
7	252	vomeronasal 2, receptor, 15	LOC628326	I	7276756	390	11	380
7	253	vomeronasal 2, receptor, 5 isoform 1	Vmn2r32	I	7426056	388	11	378
7	254	similar to vomeronasal 2, receptor, 14 isoform 1	LOC545908	I	7635348	388	11	378
		similar to vomeronasal 2, receptor, 14 isoform 2						
7	255	similar to vomeronasal 2, receptor, 15 isoform 2	LOC628326	I	7840959	387	11	377
		similar to vomeronasal 2, receptor, 15 isoform 1		I				
7	256	similar to vomeronasal 2, receptor, 14 isoform 1	LOC545908	I	8074941	387	11	377
		similar to vomeronasal 2, receptor, 14 isoform 2						
7	257	similar to vomeronasal 2, receptor, 15 isoform 3	LOC628326	I	10413694	390	11	380
		similar to vomeronasal 2, receptor, 15 isoform 2						

7	258	similar to putative pheromone receptor isoform 1	LOC243945	I	10526833	387	11	377
7	259	similar to putative pheromone receptor isoform 1		I	10570853	388	11	378
7	260	similar to vomeronasal 2, receptor, 15 isoform 1	LOC628326	I	10684412	391	11	381
		similar to vomeronasal 2, receptor, 15 isoform 2		I				
7	261	phospholipase A2, group IVC (cytosolic, calcium-independent)	pla2g4c	E	13927170	247	16	237
7	262	kallikrein 26	Klk1b26	E	51269907	561	25	551
7	263	leucine zipper protein 2	Luzp2	I	62318828	361	24	351
7	264	gamma-aminobutyric acid (GABA-A) receptor, subunit gamma 3	Gabrb3	I	64570061	410	12	400
7	265	ubiquitin protein ligase E3A isoform 2	Ube3a	E	66539644	381	23	371
		ubiquitin protein ligase E3A isoform 3		I				
7	266	multiple C2 domains, transmembrane 2	Mctp2	I	79231749	515	27	505
7	267	multiple C2 domains, transmembrane 2	Mctp2	I	79317556	360	16	350
7	268	solute carrier organic anion transporter family, member 3...	Slco3a1	I	81551764	272	18	262
		solute carrier organic anion transporter family, member 3...						
7	269	cytoplasmic polyadenylation element binding protein 1		I	88534843	379	6	369
7	270	cytoplasmic polyadenylation element binding protein 1	Cpeb1	I	88564601	418	12	408
7	271	carboxypeptidase 4, cytosolic	Agbl1	I	83860962	420	9	410
7	272	WAS protein homology region 2 domain containing 1	Whamm	I	88720002	478	16	468
7	273	hypothetical protein LOC70178		I	91274779	390	9	380
7	274	similar to putative pheromone receptor	LOC272377	I	93309701	526	10	516
7	275	similar to putative pheromone receptor	LOC272377	I	93944986	375	23	365
7	276	glutamate receptor, metabotropic 5	Grm5	I	95031289	476	19	466
7	277	spondin 1, (f-spondin) extracellular matrix protein	Spon1	I	120915658	315	9	305
7	278	xylosyltransferase 1	Xylt1	I	124625598	337	16	327
7	279	ADAMTS-like 3	Adamtsl3	I	89504603	409	18	399
7	280	NADPH oxidase 4	Nox4	I	94461886	416	21	406
7	281	chapsyn-110	LOC233498	I	98247576	368	9	358
7	282	chapsyn-110	LOC233498	I	98784548	218	7	208
7	283	glycerophosphodiester phosphodiesterase domain containing 4	Gdpd4	E	105148163	365	19	355
7	284	olfactory receptor 485	Olfr485	E	115303056	359	24	349
7	285	olfactory receptor 488	Olfr485	E	115399338	367	24	357
7	286	ATP-binding cassette transporter sub-family A member 16	Abca16	I	127599420	319	18	309
7	287	voltage-dependent calcium channel gamma-3 subunit	Cacng3	I	129860461	210	12	200
7	288	sodium/glucose cotransporter KST1	Slc5a11	I	130415134	415	13	405
7	289	nadrin	Arhgap17	I	130504948	384	19	374
7	290	protein phosphatase 2A, regulatory subunit B, delta isoform	Ppp2r2d	I	146049738	407	20	397

7	291	deformed epidermal autoregulatory factor 1	Deaf1	I	148502626	566	9	556
8	292	tubulin, gamma complex associated protein 3	Tubgcp3	I	12618341	408	28	398
8	293	CUB and Sushi multiple domains 1	Csmd1	I	16285426	156	29	146
8	294	CUB and Sushi multiple domains 1	Csmd1	I	17025707	332	34	322
8	295	hypothetical protein		I	18401736	460	17	450
8	296	transmembrane phosphatase with tensin homology	Tpte	I	23418114	212	22	202
8	297	RNA binding protein gene with multiple splicing isoform 2	Rbpms	I	34993344	354	24	344
		RNA binding protein gene with multiple splicing isoform 1						
8	298	hypothetical protein LOC212326	Fam149a	I	46457405	490	7	480
8	299	odd Oz/ten-m homolog 3	Odz3	I	49599925	353	11	343
8	300	annexin A10	Anxa10	I	63031300	402	28	392
8	301	membrane-associated ring finger (C3HC4) 1	March1	I	67400091	360	23	350
8	302	mucoepidermoid carcinoma translocated 1	Crtc1	I	71409294	229	19	219
8	303	splicing factor 4	Sf4	I	71039197	369	8	359
8	304	NYD-SP14 protein	LOC234470	I	79353657	364	8	354
8	305	hypothetical protein LOC622675		I	80049056	352	29	342
8	306	ring finger protein 150	Rnf150	I	83902135	374	16	364
8	307	cadherin 8 isoform 1	Cdh8	I	100084256	349	20	339
		cadherin 8 isoform 2		I				
8	308	cadherin 8 isoform 1	Cdh8	I	100267826	407	25	397
8		cadherin 8 isoform 2		I				
8	309	BDG-29 protein	Zcchc14	I	122618819	418	15	408
8	310	pecanex-like 2 isoform 2	Pcnxl2	I	126815152	406	13	396
8	311	neuropilin 1	Nrp1	I	129478293	406	9	396
8	312	cadherin 8 isoform 1	Cdh8	I	100024987	373	9	363
8		cadherin 8 isoform 2						
8	313	ring finger and WD repeat domain 3	Rfwd3	I	112283302	403	13	393
8	314	WW-domain oxidoreductase	Wwox	I	116256735	346	19	336
8	315	polycystin 1-like 2	Pkd1l2	I	118006300	385	16	375
8	316	cadherin 13	Cdh13	I	119983673	431	30	421
9	317	glutamate receptor, ionotropic, AMPA4 (alpha 4)	Gria4	I	4721178	167	27	157
9	318	dynein, cytoplasmic, heavy chain 2	Dync2h1	I	6994726	170	9	160
9	319	dynein, cytoplasmic, heavy chain 2	Dync2h1	I	7137686	379	23	369
9	320	hypothetical protein LOC234915	AK129341	I	8132266	233	24	223
9	321	contactin 5	Cntn5	I	10125879	515	31	505
9	322	glutamate receptor, ionotropic, AMPA4 (alpha 4)	Gria4	E	4455699	402	12	392
9	323	contactin 5	Cntn5	I	9859961	556	16	546

9	324	piwi-like 4	Piwil4	I	14525114	399	30	389
9	325	crossveinless 2	Bmper	I	23193207	412	23	402
9	326	dpy-19-like 1	Dpy19l1	E	24218458	350	26	340
9	327	membrane protein mKirre	Kirrel3	I	34725113	335	12	325
9	328	hypothetical protein LOC235184	BC024479	I	37307378	363	7	353
9	329	ferredoxin 1	Fdxacb1	I	51756143	379	13	369
9	330	myotubularin related protein 2	Mtmr2	E	13599378	465	17	455
9	331	embryonic development factor 1	Cwc15	I	14309031	317	9	307
9	332	FAT tumor suppressor homolog 3	Fat3	I	15999397	579	7	569
9	333	mucin 16	Muc16	E	18366682	384	10	374
9	334	HERPUD family member 2	Herpud2	I	24938159	348	22	338
9	335	opioid binding protein/cell adhesion molecule-like	Opcml	I	27921034	502	24	492
9	336	neurotrimin	Ntm	I	29416172	419	17	409
9	337	pseudouridine synthase 3	Pus3	E	35373551	238	23	228
9	338	myeloid/lymphoid or mixed-lineage leukemia 1	Mll1	I	44659225	373	30	363
9	339	Dmx-like 2	Dmxl2	I	54304231	405	13	395
9	340	NADPH cytochrome B5 oxidoreductase	Cyb5r4	I	86936557	392	26	382
9	341	calsyntenin 2	Clstn2	I	97371105	346	9	336
9	342	stromal antigen 1	Stag1	I	100735510	161	17	151
9	343	pleckstrin homology domain interacting protein	Phip	I	82841400	374	31	364
9	344	RAS protein-specific guanine nucleotide-releasing factor ...	Rasgrf1	I	89887453	229	22	219
9	345	stromal antigen 1	Stag1	E	100791155	389	23	379
9	346	copine IV	Cpne4	I	104638130	347	15	337
9	347	copine IV	Cpne4	I	104908676	501	7	491
9	348	centrosomal protein 63	Cep63	E	102517200	378	23	368
9	349	copine IV	Cpne4	I	104742052	423	29	413
9	350	F-box domain containing protein	Fbxw21	I	109055893	292	28	282
9	351	5-azacytidine induced 2 isoform a	Azi2	E	117968575	291	20	281
9	352	dynein, cytoplasmic, light intermediate chain 1	Dync1li1	I	114630809	472	31	462
9	353	integrin alpha 9	Itga9	I	118609036	366	27	356
9	354	CTD (carboxy-terminal domain, RNA polymerase II, polypept...	Ctdspl	I	118915950	585	12	575
9	355	hypoxia induced gene 1	Higd1a	I	121760033	394	8	384
10	356	synaptic nuclear envelope 1 isoform 1	Syne1	I	4813346	314	9	304
10	357	glycine receptor, alpha 1 subunit	Glra1	I	28221913	370	27	360
10	358	glutamate receptor, ionotropic, AMPA1 (alpha 1)	Gria1	I	29671740	591	19	581
10	359	nuclear receptor co-repressor 1	Ncor1	I	34977996	380	12	370
10	360	myosin, heavy polypeptide 2, skeletal muscle, adult	Myh2	E	39811909	621	29	611

10	361	dynein, axonemal, heavy chain 2	Dnahc2	E	42092055	363	20	353
10	362	amiloride-sensitive cation channel 1, neuronal isoform b<br>amiloride-sensitive cation channel 1, neuronal isoform a	Accn1	I	53669592	273	18	263
10	363	amiloride-sensitive cation channel 1, neuronal isoform b	Accn1	I	54454176	495	18	485
10	364	fatty acid binding protein 6, ileal (gastrotropin)	Fabp6	I	16211289	383	31	373
10	365	sarcoglycan, delta (dystrophin-associated glycoprotein)	Sgcd	I	19962600	275	18	265
10	366	LYR motif containing 7	Lyrm7	I	27457394	436	11	426
10	367	folliculin	Flcn	I	32414703	343	19	333
10	368	nuclear receptor co-repressor 1	Ncor1	I	34971787	444	28	434
10	369	phosphatidylinositol glycan anchor biosynthesis, class L	Pigl	I	35119125	387	13	377
10	370	dynein, axonemal, heavy chain 9 isoform 1	LOC195118	I	38568011	384	27	374
10	371	centaurin, beta 1	Acap1	I	42502533	392	29	382
10	372	regulatory factor X domain containing 1	Rfx6	I	51441453	387	24	377
10	373	solute carrier family 35, member F1	Slc35f1	I	52570121	456	15	446
10	374	hect domain and RLD 4	Herc4	I	62767779	618	9	608
10	375	carbohydrate sulfotransferase 11	Chst11	I	82615769	372	22	362
10	376	transmembrane protein 16D	Ano4	I	88494472	364	27	354
10	377	hypothetical protein		E	97808432	475	28	465
10	378	transmembrane and tetratricopeptide repeat containing 2	Tmtc2	I	104965287	305	18	295
10	379	myopalladin	Mypn	I	62594512	391	16	381
10	380	catenin (cadherin associated protein), alpha 3<br>leucine-rich repeat transmembrane neuronal 3 protein	Ctnna3	I	63458640	350	18	340
10	381	catenin (cadherin associated protein), alpha 3	Ctnna3	I	63938665	391	19	381
10	382	catenin (cadherin associated protein), alpha 3	Ctnna3	I	64118870	161	7	151
10	383	ilvB (bacterial acetolactate synthase)-like	Ilvbl	I	78042442	264	20	254
10	384	stabilin-2	Stab2	I	86374031	495	25	485
10	385	neural precursor cell expressed, developmentally down-reg...	Nedd1	I	92167626	525	9	515
10	386	hypothetical protein LOC380654	›30485B16R	I	92461527	345	34	335
10	387	hypothetical protein		I	98920543	445	9	435
10	388	eucine-rich repeats and IQ motif containing 1	Lrriq1	I	102588972	421	20	411
10	389	hypothetical protein		I	109757886	277	9	267
10	390	cytokine induced protein 29 kDa		I	128260650	362	9	352
11	391	zinc and ring finger 3	Znrf3	I	5200663	284	17	274
11	392	hypothetical protein LOC67592	›30524B15R	E	31876641	335	15	325
11	393	SH3 and PX domains 2B	Sh3pxd2b	I	32306480	607	8	597
11	394	hypothetical protein LOC73307	'00046C09R	E	14477881	456	8	446
11	395	reticulon 4 isoform A	Rtn4	I	29614581	186	33	176

		reticulon 4 isoform B2						
11	396	hypothetical protein		I	30163988	401	27	391
11	397	cytoplasmic polyadenylation element binding protein 4	Cpeb4	I	31819312	329	8	319
11	398	odd Oz/ten-m homolog 2 isoform 1	Odz2	I	36696934	482	26	472
11	399	clathrin interactor 1	Clint1	I	45688200	487	7	477
11	400	FMS-like tyrosine kinase 4	Flt4	E	49439098	481	14	471
11	401	la related protein	ı related prot	E	57868815	360	8	350
11	402	rabaptin, RAB GTPase binding effector protein 1	Rabep1	I	70713253	455	12	445
11	403	olfactory receptor 394	Olfr394	I	73700657	293	18	283
11	404	myosin ID	Myo1d	I	80403091	452	8	442
11	405	hypothetical protein LOC103743		I	80626735	447	29	437
11	406	hypothetical protein LOC74133		I	86896273	360	28	350
11	407	pantothenate kinase 3	Pank3	I	35594396	261	20	251
11	408	folliculin interacting protein 1	Fnip1	I	54260129	441	23	431
11	409	myocardin isoform A	Myocd	I	65023915	374	7	364
		myocardin isoform B						
11	410	breast cancer 1	Brca1	I	101368730	339	7	329
11	411	SMAD specific E3 ubiquitin protein ligase 2	Smurf2	I	106696067	349	11	339
11	412	integrin alpha 3	Itga3	E	94916870	404	8	394
11	413	similar to potassium voltage-gated channel, subfamily H, ...		I	100607335	294	26	284
11	414	hypothetical protein		I	104663893	314	13	304
11	415	solute carrier family 39 (metal ion transporter), member 11	Slc39a11	I	113307883	439	27	429
12	416	hypothetical protein		I	20969580	360	11	350
12	417	O-acyltransferase (membrane bound) domain containing 2 is...	Mboat2	I	25619556	412	9	402
		O-acyltransferase (membrane bound) domain containing 2 is...						
12	418	dedicator of cytokinesis 4	Dock4	I	41382406	354	31	344
12	419	syntrophin, gamma 2	Sntg2	I	30932302	500	21	490
12	420	syntrophin, gamma 2	Sntg2	I	30939782	420	24	410
12	421	syntrophin, gamma 2	Sntg2	I	31015555	457	27	447
12	422	striatin, calmodulin binding protein 3	Strn3	I	52378290	400	12	390
12	423	A kinase (PRKA) anchor protein 6	Akap6	I	53613560	350	15	340
12	424	solute carrier family 25 (mitochondrial oxodicarboxylate ...	Slc25a21	I	57634717	425	28	415
12	425	MAM domain containing glycosylphosphatidylinositol anchor 2	Mdga2	I	67397499	436	13	426
12	426	ninein isoform 1	Nin	I	70782673	350	26	340
		ninein isoform 2						
12	427	ras homolog gene family, member J	Rhoj	I	76081187	367	27	357
12	428	ras homolog gene family, member J	Rhoj	I	76087383	436	9	426

12	429	MAM domain containing glycosylphosphatidylinositol anchor 2	Mdga2	I	67384425	323	8	313
12	430	MAM domain containing glycosylphosphatidylinositol anchor 2	Mdga2	I	67462773	392	34	382
12	431	tripartite motif protein 9	Trim9	I	71096806	278	17	268
12	432	estrogen receptor beta isoform 2<br>estrogen receptor beta isoform 1	Esr2	E	76909527	457	22	447
12	433	hypothetical protein LOC75216	)30534B04F	I	91995970	557	19	547
12	434	ribosomal protein S6 kinase, polypeptide 5	Rps6ka5	I	101518778	348	11	338
12	435	hypothetical protein LOC271036		I	102445540	392	11	382
12	436	hypothetical protein LOC77878	)30440G07F	I	111896451	267	14	257
12	437	chr2 synaptotagmin	Esyt2	I	117207776	369	24	359
12	438	neurexin III	Nrxn3	I	90770341	344	14	334
12	439	Ena-vasodilator stimulated phosphoprotein	Evl	I	109477278	531	33	521
13	440	adenosine deaminase, RNA-specific, B2	Adarb2	I	8631908	374	22	364
13	441	phenylalanine-tRNA synthetase 2 (mitochondrial) isoform 1	Fars2	I	36313091	441	12	431
13	442	guanine nucleotide binding protein (G protein), gamma 4 s...	Gng4	I	13903525	388	33	378
13	443	cell division cycle 2-like 5 (cholinesterase-related cell...	Cdk13	I	17814566	343	6	333
13	444	vacuolar protein sorting 41	Vps41	I	18811847	369	12	359
13	445	hypothetical protein LOC73102		I	34279372	259	6	249
13	446	transcription factor AP-2, alpha	Tcfap2a	I	40822334	411	19	401
13	447	AU RNA-binding enoyl-coenzyme A hydratase	Auh	I	52932205	360	18	350
13	448	human immunodeficiency virus type I enhancer binding prot...	Hivep1	I	42263930	551	12	541
13	449	dystrobrevin binding protein 1	Dtnbp1	I	45055105	305	31	295
13	450	kinesin family member 13A	Kif13a	I	46957429	510	23	500
13	451	G kinase anchoring protein 1	Gkap1	I	58338665	371	12	361
13	452	Fanconi anemia, complementation group C<br>Fanconi anemia, complementation group C	Fancc	I	63429915	239	21	229
13	453	adenylate cyclase 2	Adcy2	I	68766375	306	26	296
13	454	X-ray repair complementing defective repair in Chinese ha...	Xrcc4	I	90141752	492	26	482
13	455	death associated protein kinase 1<br>death associated protein kinase 1	Dapk1	I	60857487	401	14	391
13	456	zinc finger protein 712 isoform 1	Zfp712	E	67141562	423	33	413
13	457	elongation factor RNA polymerase II 2	Ell2	I	75898067	433	18	423
13	458	hypothetical protein LOC218343		E	76282097	404	28	394
13	459	developmental endothelial locus-1 isoform a<br>developmental endothelial locus-1 isoform b	Edil3	I	89194451	288	25	278
13	460	RAS protein-specific guanine nucleotide-releasing factor 2	Rasgrf2	I	92042923	543	12	533
13	461	a disintegrin-like and metalloprotease (reprolysin type) ...	Adamts6	I	105141649	354	26	344

13	462	hypothetical protein LOC73942	Fam151b	I	93221039	352	8		342
14	463	fragile histidine triad	Fhit	I	10778523	404	18		394
14	464	fragile histidine triad	Fhit	I	10739437	325	23		315
14	465	fragile histidine triad	Fhit	I	11055378	377	29		367
14	466	Ca<2+>dependent activator protein for secretion isoform 2	Cadps	I	13622800	454	9		444
		Ca<2+>dependent activator protein for secretion isoform 1							
14	467	large conductance calcium-activated potassium channel sub...	Kcnma1	I	24322239	321	15		311
14	468	Rho guanine nucleotide exchange factor (GEF) 3	Arhgef3	I	28156516	465	6		455
14	469	neuregulin 3	Nrg3	I	39326963	530	18		520
14	470	neuregulin 3	Nrg3	I	39561499	391	13		381
14	471	SH2 domain containing 4B	Sh2d4b	I	41695663	495	26		485
14	472	WD repeat and HMG-box DNA binding protein 1	Wdhd1	I	47868949	506	21		496
14	473	solute carrier family 35, member F4	Slc35f4	I	50083362	328	7		318
14	474	ATPase, aminophospholipid transporter-like, class I, type...	Atp8a2	I	60306699	380	14		370
14	475	hypothetical protein		I	75372835	386	25		376
14	476	dachshund 1 isoform 2	Dach1	I	98284061	420	20		410
		dachshund 1 isoform 1							
14	477	pam, highwire, rpm 1	Mycbp2	E	103696392	286	28		276
14	478	FERMRhoGEF (Arhgef) and pleckstrin domain protein 1	Farp1	I	121627221	462	6		452
14	479	FERMRhoGEF (Arhgef) and pleckstrin domain protein 1	Farp1	I	121627272	411	6		401
14	480	FERMRhoGEF (Arhgef) and pleckstrin domain protein 1	Farp1	I	121627323	391	6		381
14	481	FERMRhoGEF (Arhgef) and pleckstrin domain protein 1	Farp1	I	121627374	406	6		396
14	482	FERMRhoGEF (Arhgef) and pleckstrin domain protein 1	Farp1	I	121627425	355	6		345
14	483	integrin, beta-like 1	Itgbl1	I	124344688	379	23		369
14	484	fibroblast growth factor 14 isoform b	Fgf14	I	124621952	381	18		371
14	485	calcium channel, voltage-dependent, alpha2/delta subunit 3	Cacna2d3	I	29914253	329	9		319
14	486	glutamate receptor, ionotropic, delta 1	Grid1	I	35802184	503	20		493
14	487	neuregulin 3	Nrg3	I	40146325	371	34		361
14	488	neuregulin 3	Nrg3	I	40229621	302	18		292
14	489	hypothetical protein LOC434459	Gm5622	I	52183855	328	19		318
14	490	chromodomain helicase DNA binding protein 8	Chd8	I	52822304	451	8		441
14	491	exportin 4	Xpo4	I	58201017	255	29		245
14	492	spermatogenesis associated 13 isoform 7	Spata13	I	61373417	479	27		469
14	493	scavenger receptor class A, member 3	Scara3	I	66562722	358	31		348
14	494	hypothetical protein LOC219170	AU021034	I	78484899	348	7		338
14	495	protocadherin 17	Pcdh17	I	84915541	620	20		610
14	496	protocadherin 9	Pcdh9	I	93619904	393	15		383

14	497	protocadherin 9	Pcdh9	I	93920780	373	23	363
14	498	kelch-like 1	Klhl1	I	96773587	480	23	470
14	499	RIKEN cDNA 2810028N01	OC1001874	I	99489050	373	27	363
14	500	LIM domain only 7	Lmo7	I	102143047	455	21	445
14	501	LIM domain only 7	Lmo7	I	102253504	425	23	415
14	502	FERMRhoGEF (Arhgef) and pleckstrin domain protein 1	Farp1	I	121591367	535	25	525
15	503	complement component 9	C9	I	6409730	325	20	315
15	504	WD repeat domain 70	Wdr70	I	8017730	393	22	383
15	505	prolactin receptor	Prlr	I	10256829	149	25	139
15	506	hypothetical protein		I	14903987	515	21	505
15	507	cadherin 10	Cdh10	I	18898160	536	27	526
15	508	ank protein	Ank	I	27514809	362	12	352
15	509	hypothetical protein		I	28644055	386	13	376
15	510	semaphorin 5A	Sema5a	I	32402681	286	28	276
15	511	semaphorin 5A	Sema5a	I	32516497	618	15	608
15	512	serine/threonine kinase 3 (Ste20, yeast homolog) STK3	Stk3	I	34893220	371	16	361
15	513	vacuolar protein sorting 13B isoform 5	Vps13b	I	35451425	474	13	464
15	514	LMBR1 domain containing 2	Lmbrd2	I	9115487	338	11	328
15	515	cadherin 6	Cdh6	I	12994922	444	11	434
15	516	hypothetical protein		I	14898926	529	25	519
15	517	cadherin 12	Cdh12	I	21506312	390	34	380
15	518	plasma glutamate carboxypeptidase	Pgcp	I	33259226	476	7	466
15	519	frizzled 6	Fzd6	I	38855348	183	17	173
15	520	regulating synaptic membrane exocytosis 2	Rims2	I	39062207	422	25	412
15	521	regulating synaptic membrane exocytosis 2	Rims2	I	39235238	344	31	334
15	522	regulating synaptic membrane exocytosis 2	Rims2	I	39287410	366	7	356
15	523	polycystic kidney and hepatic disease 1-like 1	Pkhd1l1	E	44337141	400	21	390
15	524	zinc finger transcription factor TRPS1	Trps1	I	50504542	216	12	206
15	525	sterile alpha motif domain containing 12	Samd12	I	53719869	509	16	499
15	526	collagen, type XXII, alpha 1 isoform 2	Col22a1	I	71835165	564	12	554
15	527	chromatin accessibility complex 1	Chrac1	E	72923913	536	31	526
15	528	ataxin 10	Atxn10	I	85230758	307	20	297
15	529	periphilin 1 isoform 3	Pphln1	I	93274447	472	23	462
		periphilin 1 isoform 1						
15	530	huntington yeast partner C	Prpf40b	E	99134818	435	16	425
15	531	angiopoietin 1	Angpt1	I	42367140	354	15	344
15	532	polycystic kidney and hepatic disease 1-like 1	Pkhd1l1	E	44367060	525	30	515

15	533	zinc finger transcription factor TRPS1	Trps1	I	50593248	356	27	346
15	534	sterile alpha motif domain containing 12	Samd12	I	53417542	451	23	441
15	535	non-SMC element 2 homolog (MMS21, S. cerevisiae)	Nsmce2	I	59348227	409	10	399
15	536	PHD finger protein 20-like 1	Phf20l1	I	66418354	401	30	391
15	537	kinesin family member 21A	Kif21a	E	90772034	535	27	525
15	538	hypothetical protein LOC320709		I	94658072	384	25	374
15	539	hypothetical protein		I	94952443	260	28	250
15	540	insulin-like growth factor binding protein 6	Igfbp6	I	101977515	556	23	546
16	541	ataxin 2 binding protein 1 isoform gamma	A2bp1	I	7078710	320	19	310
		ataxin 2 binding protein 1 isoform alpha						369
16	542	glutamate receptor, ionotropic, NMDA2A (epsilon 1)	Grin2a	I	9938844	379	13	393
16	543	activating transcription factor 7 interacting protein 2 i...	Atf7ip2	I	10213422	437	24	427
16	544	EF hand calcium binding domain 1	Efcab1	I	14923585	390	9	380
16	545	protein kinase, DNA activated, catalytic polypeptide	Prkdc	I	15689763	377	17	367
16	546	hypothetical protein LOC224008	310008H04R	I	15958050	324	20	314
16	547	eukaryotic translation initiation factor 4, gamma 1 isofo...	Eif4g1	I	20691747	343	18	333
		eukaryotic translation initiation factor 4, gamma 1 isofo...						356
16	548	syntaxin binding protein 5-like	Stxbp5l	I	37334856	563	24	553
16	549	CD200 receptor 3	Cd200	I	44976537	462	9	452
16	550	interphotoreceptor matrix proteoglycan 2	Impg2	I	56262772	346	20	336
16	551	protein S (alpha)	Pros1	I	62915111	232	25	222
16	552	similar to zinc finger protein 654		I	64792644	373	25	363
16	553	hypothetical protein LOC74478	LOC546679	I	11643739	358	34	348
16	554	kelch-like 22	Klhl22	I	17782326	516	28	506
16	555	mitogen-activated protein kinase kinase kinase 13	Map3k13	I	21913164	550	15	540
16	556	LIM domain containing preferred translocation partner in ...	Lpp	I	24870927	420	28	410
16	557	hypothetical protein LOC239789	Gm606	I	26977688	366	25	356
16	558	protein tyrosine phosphatase-like protein PTPLB	Ptplb	I	35053863	332	26	322
16	559	EG433016 protein	Gm5483	I	36187161	393	32	383
16	560	similar to stefin A2	LOC639118	I	36309147	384	20	374
16	561	syntaxin binding protein 5-like	Stxbp5l	I	37282362	283	12	273
16	562	pleckstrin homology-like domain, family B, member 2	Phldb2	E	45824665	439	30	429
16	563	TBC1 domain family, member 23	Tbc1d23	I	57229479	338	18	328
16	564	hypothetical protein		I	58073593	451	14	441
16	565	Eph receptor A6	Epha6	I	60452664	323	20	313
16	566	Eph receptor A3	Epha3	I	63563576	388	6	378
16	567	glucan (1,4-alpha-), branching enzyme 1	Gbe1	I	70390635	358	25	348

16	568	glucan (1,4-alpha-), branching enzyme 1	Gbe1	I	70565132	307	19	297
16	569	roundabout homolog 2	Robo2	I	74289987	394	8	384
16	570	dual-specificity tyrosine-(Y)-phosphorylation regulated k...	Mapk1	I	94902969	398	31	388
16	571	mitochondrial ribosomal protein L39	Mrpl39	I	84728583	391	33	381
16	572	tetratricopeptide repeat domain 3	Ttc3	I	94619269	426	23	416
16	573	cell adhesion molecule JCAM	Igsf5	I	96605291	502	25	492
17	574	hypothetical protein		I	6672015	384	9	374
17	575	hypothetical protein		I	6877217	384	9	374
17	576	AT rich interactive domain 1B (Swi1 like) isoform 1	LOC634040	I	5258829	424	17	414
		AT rich interactive domain 1B (Swi1 like)						445
17	577	synaptojanin 2	Synj2	E	6040365	455	28	
17	578	similar to UNC-93A protein	LOC545233	I	7571465	233	12	223
17	579	solute carrier family 22 member 1	Slc22a1	I	12842534	391	21	
17	580	parkin	Park2	I	11357706	536	16	
17	581	unc-93 homolog A	Unc93a	I	13305803	222	12	381
17	582	similar to putative pheromone receptor	LOC636884	I	19796707	367	23	357
17	583	BTB (POZ) domain containing 9	Btbd9	I	30559824	504	26	350
17	584	TBC1 domain family, member 5	Tbc1d5	I	50909323	534	31	524
17	585	TBC1 domain family, member 5	Tbc1d5	I	50920992	384	14	374
17	586	EGF-like module containing, mucin-like, hormone receptor-...	Emr4	I	57602531	405	11	395
17	587	contactin associated protein-like 5C	Cntnap5c	I	58275671	319	26	309
17	588	latent transforming growth factor beta binding protein 1 ...	Ltbp1	I	75627238	416	7	406
		latent transforming growth factor beta binding protein 1 ...						142
17	589	CCAAT/enhancer binding protein zeta	Cebpz	I	79323093	152	22	
17	590	solute carrier family 8 (sodium/calcium exchanger), member 1	Slc8a1	I	81942896	364	16	354
17	591	thyroid adenoma associated	Thada	I	84681549	262	16	252
17	592	leucine-rich PPR motif-containing protein	Lrpprc	I	85128981	390	30	380
17	593	hypothetical protein LOC73582	'00106N22F	I	85808749	315	15	305
17	594	potassium channel, subfamily K, member 12	Kcnk12	I	88195029	538	7	528
17	595	secreted modular calcium-binding protein 2	Smoc2	I	14450548	367	33	357
17	596	similar to putative pheromone receptor	LOC636884	I	19196229	409	26	399
17	597	similar to putative pheromone receptor	LOC636884	I	19812769	361	28	351
17	598	serine/threonine kinase 38	Stk38	I	29129187	379	31	369
17	599	runt related transcription factor 2	Runx2	I	44809710	272	12	262
17	600	hypothetical protein		I	51422743	304	13	294
17	601	discs large homolog-associated protein 1 isoform 1	Dlgap1	I	71093228	355	19	345
		discs large homolog-associated protein 1 isoform 2						

17	602	hypothetical protein LOC73582	700106N22R	I	85541278	189	15	179
17	603	neurexin I	Nrxn1	I	91189233	402	12	392
18	604	expressed sequence AW060714	AW060714	I	34636297	256	20	246
18	605	heat shock protein 9A	Hspa9	I	35108239	401	26	391
18	606	matrin 3	Matr3	I	35735465	365	30	355
18	607	IK cytokine	Ik	I	36905526	421	13	411
18	608	Yip1 domain family, member 5	Yipf5	I	40373433	476	21	466
18	609	protein phosphatase 2 (formerly 2A), regulatory subunit B...	Ppp2r2b	I	42923282	315	14	305
		protein phosphatase 2 (formerly 2A), regulatory subunit B...						
18	610	hypothetical protein LOC74574		I	47039351	309	18	299
18	611	GRAM domain containing 3	Gramd3	I	56643931	283	28	273
18	612	deleted in colorectal carcinoma	Dcc	I	71861094	391	14	381
18	613	deleted in colorectal carcinoma	Dcc	I	72442999	474	14	464
18	614	hypothetical protein LOC71531	933408B17R	I	62891808	267	34	257
18	615	hypothetical protein LOC52662	D18Ertd653e	I	63731041	475	24	465
18	616	Msx-interacting-zinc finger	Pias2	I	76043090	417	23	407
18		Msx-interacting-zinc finger						
18	617	ring finger protein 165	Rnf165	I	77305377	406	20	396
18	618	zinc finger protein 407	Rnf165	I	81959529	580	7	570
18	619	platelet and T cell activation antigen 1 isoform b	Cd226	I	82590332	291	27	281
		platelet and T cell activation antigen 1 isoform a						
18	620	F-box protein 15	Fbxo15	I	82759001	601	29	591
19	621	suppressor of variegation 4-20 homolog 1	Suv420h1	I	3805940	605	21	595
19	622	spectrin beta 3	Spnb3	I	4728528	462	11	452
19	623	hypothetical protein LOC434674	Gm5631	I	8204852	402	14	
19	624	integral membrane transport protein UST1R	730048C13R	I	8475461	327	14	
19	625	transient receptor potential cation channel, subfamily M,...	Trpm3	I	18931629	381	21	371
19	626	RAR-related orphan receptor beta isoform 1	Rorb	I	19071353	460	11	450
		RAR-related orphan receptor beta isoform 2		I				
19	627	very low density lipoprotein receptor	Vldlr	I	27319539	455	8	445
19	628	hypothetical protein LOC381218	430402I18R	I	29027075	364	25	354
19	629	adenylate kinase 3	Ak3	I	29114367	393	13	383
19	630	membrane-spanning 4-domains, subfamily A, member 4D	Ms4a4d	E	11632295	455	10	445
19	631	cell division cycle 37 homolog (S. cerevisiae)-like 1	Cdc37l1	I	29072869	397	30	387
19	632	protein kinase, cGMP-dependent, type I beta isoform	Prkg1	I	31557872	379	30	369
		protein kinase, cGMP-dependent, type I alpha isoform		I				
19	633	cytoplasmic polyadenylation element binding protein 3	Cpeb3	I	37153964	383	21	373

19	634	leucine-rich repeat LGI family, member 1	Lgi1	E	38339340	343	24	333
19	635	cyclin M2	Cnnm2	I	46896833	391	26	381
19	636	SORCS receptor 1	Sorcs1	I	50380435	413	31	403
19	637	Max interactor 1 isoform b	Mxi1	I	53430348	513	34	503
		Max interactor 1 isoform a						353
19	638	chondroitin sulfate proteoglycan 6	Smc3	I	53693119	363	23	
19	639	NHL repeat containing 2	Nhlrc2	I	56653429	244	26	234
19	640	similar to Sm protein G	OC1000449(	I	57660698	585	12	575
19	641	attractin-like 1	Atrnl1	I	58055673	432	13	422
X	642	shroom family member 4	Shroom4	I	6016343	265	19	255
X	643	shroom family member 4	Shroom4	I	6042014	367	15	357
X	644	ubiquitously transcribed tetratricopeptide repeat gene, X...	Kdm6a	I	17852438	359	9	349
X	645	cytochrome b-245, beta polypeptide	Cybb	E	9026407	365	26	355
X	646	ubiquitin specific protease 9, X chromosome	Usp9x	I	12728403	398	19	388
X	647	ubiquitously transcribed tetratricopeptide repeat gene, X...	Kdm6a	I	17800937	374	29	364
X	648	glypican 3	Gpc3	I	49683579	434	29	424
X	649	glutamate receptor, ionotropic, AMPA3 (alpha 3)	Gria3	I	38910147	432	28	422
X	650	odd Oz/ten-m homolog 1	Odz1	I	40282787	298	19	288
X	651	odd Oz/ten-m homolog 1	Odz1	I	40382240	312	21	302
X	652	serine/threonine protein kinase MST4	310018G03R	I	48218018	364	9	354
X	653	glypican 3	Gpc3	I	49817936	345	23	335
X	654	solute carrier family 9 (sodium/hydrogen exchanger), isof...	Slc9a6	I	53869691	390	27	380
X	655	gamma-aminobutyric acid (GABA-A) receptor, subunit alpha 3	Gabra3	I	69682927	364	30	354
X	656	c6.1a protein	Brcc3	I	72664755	374	12	364
X	657	von Hippel-Lindau binding protein 1	Vbp1	E	72776372	408	8	398
X	658	hypothetical protein		I	76666292	324	22	314
X	659	polymerase (DNA directed), alpha 1	Pola1	I	90650142	364	25	354
X	660	eukaryotic translation initiation factor 2, subunit 3, st...	Eif2s3x	E	91441513	470	31	460
X	661	G protein-coupled receptor 112	Gpr112	I	54193993	358	9	348
X	662	Atpase, class VI, type 11C isoform b	Atp11c	I	57493333	355	29	345
		Atpase, class VI, type 11C isoform a						
X	663	growth factor receptor bound protein 2-associated protein 3	Gab3	I	72301545	407	24	397
		growth factor receptor bound protein 2-associated protein 3						
X	664	c6.1a protein	Brcc3	I	72690339	570	22	560
X	665	dystrophin, muscular dystrophy	Dmd	I	80479163	207	17	197
X	666	dystrophin, muscular dystrophy	Dmd	I	81283341	360	15	350
X	667	dystrophin, muscular dystrophy	Dmd	E	81719295	462	15	452

X	668	glycerol kinase isoform 1	Gyk	I	82949975	370	7	360
		glycerol kinase isoform 2		I				
X	669	similar to interleukin 1 receptor accessory protein-like ...	LOC279557	I	84340169	384	12	374
X	670	similar to interleukin 1 receptor accessory protein-like ...	LOC279557	I	84460975	369	24	359
X	671	polymerase (DNA directed), alpha 1	Pola1	I	90628589	376	25	366
X	672	pyruvate dehydrogenase kinase, isoenzyme 3	Pdk3	I	91070509	411	24	401
X	673	dachshund 2	Dach2	I	110805240	463	21	453
X	674	similar to testis expressed gene 16	OC1000461	I	109131759	254	18	244
X	675	hypothetical protein LOC67715	)10106E10R	I	109664817	287	18	277
X	676	choroidermia	Chm	I	110187239	365	23	355
X	677	diaphanous homolog 2	Diap2	I	126753608	362	9	352
X	678	zinc finger, matrin type 1	Zmat1	I	131529228	304	31	294
X	679	interleukin 1 receptor accessory protein-like 2	Il1rapl2	I	134555317	511	8	501
X	680	hypothetical protein LOC331537	230019M04R	I	136614510	188	13	178
X	681	cleavage stimulation factor, 3' pre-RNA subunit 2	Cstf2	E	130607365	588	17	578
X	682	interleukin 1 receptor accessory protein-like 2	Il1rapl2	I	134885608	373	9	363
X	683	ring finger protein 128	Rnf128	I	136152453	344	33	334
X	684	RNA binding motif protein 41	Rbm41	I	136513200	412	13	402
X	685	glucocorticoid-induced leucine zipper isoform 1	Tsc22d3	I	137078896	384	16	374
X	686	acyl-CoA synthetase long-chain family member 4 isoform 1	Acsl4	E	138773955	277	30	
		acyl-CoA synthetase long-chain family member 4 isoform 2		I				436
X	687	p21 (CDKN1A)-activated kinase 3		I	140151397	446	27	354
X	688	doublecortin	Dcx	I	140343625	364	30	170
X	689	transient receptor potential cation channel, subfamily C,...	Trpc5	I	140836850	180	25	
X	690	5-hydroxytryptamine (serotonin) receptor 2C	Htr2c	I	143542689	347	22	337
X	691	connector enhancer of kinase suppressor of Ras 2	Cnksr2	I	154283275	424	31	414
X	692	protein phosphatase with EF hand calcium-binding domain 1	Ppef1	I	157087588	168	15	158
X	693	PDZ domain containing 10	Frmpd4	I	164231663	483	31	473
X	694	PDZ domain containing 10	Frmpd4	I	164330477	390	17	380
X	695	Rho GTPase activating protein 6 isoform a	Arhgap6	I	165322945	420	27	410
X	696	Rho GTPase activating protein 6 isoform a	Arhgap6	I	165524835	423	14	413
		Rho GTPase activating protein 6 isoform b						
X	697	procollagen, type IV, alpha 5	Col4a5	I	137935411	549	9	539
X	698	Nance-Horan syndrome	Nhs	I	158438559	474	18	464
X	699	gamma-taxilin	)32441K18R	I	159262560	362	11	352
X	700	cytidine triphosphate synthase II	Ctps2	I	159454313	448	26	438
X	701	BMX non-receptor tyrosine kinase	Bmx	E	160685537	186	12	176

X	702	Rho GTPase activating protein 6 isoform a	Arhgap6	I	165448644	427	22	417

Supplemental Table S 9: analysis of the number of cogenes mapping within genes.

Numbers of genes with X cogenes	X cogenes	rate
449	1	84,56%
66	2	12,43%
10	3	1,88%
3	4	0,56%
0	5	0,00%
2	6	0,38%
1	12	0,19%

Category	Term	Count	%	PValue	fold Enrichment	Bonferroni	Benjamini
SP_PIR_KEYWORDS	alternative splicing	227	44.773175542406314	4.6427441348876877E-29	512:46:00	1.4763926348942845E-26	.4763926348942845E-26
UP_SEQ_FEATURE	splice variant	223	43.98422090729783	1.4635869966318711E-24	856:13:00	2.3666201735537354E-21	.3666201735537354E-2
UP_TISSUE	Brain	273	53.84615384615385	1.4416143634642768E-17	125:06:00	2.277750694273557E-15	2.277750694273557E-15
UP_SEQ_FEATURE	domain:EGF-like 1	21	4.142011834319527	8.940513850675767E-12	1041:44:00	1.4456812635899041E-8	7.228406317949521E-9
SP_PIR_KEYWORDS	phosphoprotein	229	45.16765285996055	3.6311340539997333E-10	969:06:00	1.1547006351086964E-7	5.7735033420769355E-8
UP_SEQ_FEATURE	domain:EGF-like 2	17	3.353057199211045	3.6451379159606417E-10	602:49:00	5.894185641786009E-7	1.964728932879467E-7
GOTERM_BP_FAT	GO:0007155~cell adhesion	43	8.481262327416173	4.995287935423373E-10	465:18:00	8.317150259617989E-7	8.317150259617989E-7
GOTERM_BP_FAT	GO:0022610~biological adhesion	43	8.481262327416173	5.302398271387377E-10	99:54:00	8.828489641565795E-7	4.4142457955587133E-7
SP_PIR_KEYWORDS	egf-like domain	26	5.128205128205128	7.541869857922776E-10	382:45:00	2.3983141672623276E-7	7.994381190368216E-8
UP_TISSUE	Cerebellum	80	15.779092702169626	1.3296832736434266E-9	376:02:00	2.1008993189575165E-7	1.0504497149899095E-7
UP_SEQ_FEATURE	compositionally biased region:Ser-rich	34	6.70611439842209	5.780202058866013E-9	742:44:00	9.346543135002605E-6	2.3366439736438593E-6
INTERPRO	IPR013032:EGF-like region, conserved site	29	5.719921104536489	9.538820383381808E-9	481:53:00	8.251045645502053E-6	8.251045645502053E-6
INTERPRO	IPR006210:EGF-like	23	4.536489151873767	1.4587534912419551E-8	407:47:00	1.26181382210655E-5	6.30908901289029E-6
INTERPRO	IPR000742:EGF-like, type 3	22	4.339250493096647	4.9566806372432665E-8	901:27:00	4.2874369402644566E-5	1.429166071809096E-5
INTERPRO	IPR006209:EGF	17	3.353057199211045	1.9421569535272787E-7	242:24:00	1.6798248211424838E-4	4.1998266236076276E-5
SP_PIR_KEYWORDS	cell adhesion	30	5.9171597633136095	2.33172871925803E-7	642:15:00	7.414623294099698E-5	1.8537073663504877E-5
SP_PIR_KEYWORDS	glycoprotein	139	27.416173570019726	3.7326789558502797E-7	140:05:00	1.1869216847892883E-4	2.373956080248263E-5
UP_TISSUE	Brain cortex	38	7.495069033530571	5.624766443818292E-7	744:15:00	8.886738586477705E-5	2.9623339488527023E-5
GOTERM_MF_FAT	GO:0005509~calcium ion binding	47	9.270216962524655	8.340762642158162E-7	656:20:00	4.1194897204444025E-4	4.1194897204444025E-4
GOTERM_CC_FAT	GO:0005886~plasma membrane	118	23.274161735700197	1.0328083970172322E-6	950:39:00	3.2218447370879755E-4	3.2218447370879755E-4
UP_SEQ_FEATURE	topological domain:Extracellular	97	19.13214990138067	1.2026590691168901E-6	681:36:00	1033:14:00	3.888645494062315E-4
SP_PIR_KEYWORDS	membrane	191	37.67258382642998	1.651741766592107E-6	956:01:00	5.251163936671466E-4	8.75385542048468E-5
UP_SEQ_FEATURE	domain:EGF-like 3	12	2.366863905325444	1.6667009441297183E-6	161:08:00	868:30:00	4.49075414206912E-4
GOTERM_CC_FAT	GO:0045202~synapse	26	5.128205128205128	1.8402322553838105E-6	1061:35:00	5.739881976704053E-4	2.870352934650766E-4
SMART	SM00181:EGF	23	4.536489151873767	2.212354153557736E-6	732:59:00	4.5121891667498915E-4	4.5121891667498915E-4
UP_SEQ_FEATURE	domain:EGF-like 8	8	1.5779092702169626	4.772036284928634E-6	805:11:00	1073:20:00	165:59:00
INTERPRO	IPR012680:Laminin G, subdomain 2	9	1.7751479289940828	5.600206201827588E-6	128:01:00	847:27:00	9.683692133772848E-4
SP_PIR_KEYWORDS	ion transport	34	6.70611439842209	5.63007018134218E-6	995:36:00	433:42:00	2.557340598456914E-4
UP_SEQ_FEATURE	domain:Fibronectin type-III 1	15	2.9585798816568047	6.028776287425965E-6	436:22:00	931:13:00	632:53:00
GOTERM_MF_FAT	GO:0022838~substrate specific channel activit	26	5.128205128205128	6.552413642849687E-6	253:02:00	149:26:00	694:45:00
GOTERM_CC_FAT	GO:0005887~integral to plasma membrane	34	6.70611439842209	7.171833632829141E-6	925:15:00	568:11:00	7.455952780617281E-4
GOTERM_MF_FAT	GO:0015267~channel activity	26	5.128205128205128	8.151154291355393E-6	749:40:00	963:43:00	52:58:00
GOTERM_MF_FAT	GO:0022803~passive transmembrane transpo	26	5.128205128205128	8.151154291355393E-6	749:40:00	963:43:00	52:58:00
INTERPRO	IPR003961:Fibronectin, type III	18	3.5502958579881656	9.641843928693978E-6	367:55:00	291:05:00	633:43:00
UP_TISSUE	Diencephalon	25	4.930966469428008	1.0502925253888818E-5	993:12:00	565:44:00	4.1478168047737185E-4
GOTERM_MF_FAT	GO:0005216~ion channel activity	25	4.930966469428008	1.1549613368148355E-5	49:23:00	193:29:00	436:59:00
SP_PIR_KEYWORDS	synapse	19	3.7475345167652856	1.177224153908045E-5	150:34:00	648:16:00	4.6783988436494006E-4
SP_PIR_KEYWORDS	ionic channel	23	4.536489151873767	1.3389440257936286E-5	571:59:00	199:34:00	4.729848304302342E-4
GOTERM_CC_FAT	GO:0044459~plasma membrane part	73	14.398422090729785	1.3878362627431631E-5	1030:59:00	974:39:00	04:19:00
GOTERM_CC_FAT	GO:0031226~intrinsic to plasma membrane	34	6.70611439842209	1.6000458068029487E-5	937:20:00	528:54:00	9.979382992398866E-4
INTERPRO	IPR001791:Laminin G	9	1.7751479289940828	1.7133889116869464E-5	563:17:00	217:02:00	994:11:00

SP_PIR_KEYWORDS	calcium	40	7.889546351084813	1.756364853964047E-5	723:58:00	785:27:00	5.583729802318693E-4
GOTERM_CC_FAT	GO:0044456~synapse part	19	3.7475345167652856	1.7975228091412067E-5	31:46:00	729:01:00	9.3428354678049E-4
INTERPRO	IPR000421:Coagulation factor 5/8 type, C-term	7	1.3806706114398422	1.976819221266106E-5	320:17:00	204:34:00	188:15:00
GOTERM_BP_FAT	GO:0007267~cell-cell signaling	22	4.339250493096647	2.1376134765353594E-5	1090:19:00	375:41:00	966:44:00
UP_SEQ_FEATURE	domain:Fibronectin type-III 2	14	2.7613412228796843	2.6366159420154017E-5	838:59:00	873:24:00	297:51:00
GOTERM_BP_FAT	GO:0006796~phosphate metabolic process	44	8.678500986193294	2.728550049705614E-5	573:29:00	116:02:00	542:12:00
GOTERM_BP_FAT	GO:0006793~phosphorus metabolic process	44	8.678500986193294	2.728550049705614E-5	573:29:00	116:02:00	542:12:00
INTERPRO	IPR013320:Concanavalin A-like lectin/glucanas	11	2.1696252465483234	2.7550269136734724E-5	1026:29:00	417:09:00	71:59:00
GOTERM_CC_FAT	GO:0045211~postsynaptic membrane	14	2.7613412228796843	3.567111284781752E-5	796:41:00	268:52:00	322:33:00
GOTERM_MF_FAT	GO:0022836~gated channel activity	21	4.142011834319527	3.877546391435018E-5	910:08:00	797:58:00	651:00:00
UP_SEQ_FEATURE	glycosylation site:N-linked (GlcNAc...)	131	25.83826429980276	4.085362418502988E-5	853:57:00	506:51:00	1077:47:00
GOTERM_BP_FAT	GO:0016337~cell-cell adhesion	19	3.7475345167652856	4.2267139339169746E-5	565:04:00	445:15:00	757:01:00
SP_PIR_KEYWORDS	cell membrane	72	14.201183431952662	4.720407719723564E-5	404:44:00	315:43:00	922:36:00
UP_SEQ_FEATURE	domain:EGF-like 7	7	1.3806706114398422	4.7720887921960266E-5	218:09:00	911:23:00	865:50:00
GOTERM_BP_FAT	GO:0006468~protein amino acid phosphorylati	35	6.903353057199212	5.434006171722484E-5	749:21:00	867:49:00	316:46:00
GOTERM_BP_FAT	GO:0007268~synaptic transmission	16	3.155818540433925	5.935332277282263E-5	663:27:00	547:32:00	54:54:00
GOTERM_MF_FAT	GO:0008092~cytoskeletal protein binding	26	5.128205128205128	6.724642346715097E-5	327:53:00	900:18:00	167:29:00
KEGG_PATHWAY	mmu05412:Arrhythmogenic right ventricular ca	10	1.9723865877712032	7.162616032748928E-5	773:48:00	980:40:00	980:40:00
UP_SEQ_FEATURE	topological domain:Cytoplasmic	109	21.499013806706113	7.622400559001152E-5	760:12:00	976:23:00	1051:02:00
INTERPRO	IPR013111:EGF, extracellular	10	1.9723865877712032	8.406240621163201E-5	874:29:00	1002:52:00	540:11:00
INTERPRO	IPR001245:Tyrosine protein kinase	13	2.564102564102564	8.75466300063645E-5	475:28:00	1020:46:00	68:45:00
UP_TISSUE	Olfactory brain	18	3.5502958579881656	8.79387011137347E-5	532:31:00	386:41:00	555:10:00
INTERPRO	IPR008957:Fibronectin, type III-like fold	16	3.155818540433925	1.0543651589278379E-4	154:35:00	365:08:00	717:29:00
SMART	SM00231:FA58C	7	1.3806706114398422	1.0582065830491764E-4	491:35:00	533:14:00	202:39:00
INTERPRO	IPR013098:Immunoglobulin I-set	13	2.564102564102564	1.1027626222350169E-4	50:15:00	310:52:00	1031:52:00
GOTERM_CC_FAT	GO:0043005~neuron projection	19	3.7475345167652856	1.199918528708406E-4	732:12:00	244:49:00	407:24:00
SP_PIR_KEYWORDS	postsynaptic cell membrane	12	2.366863905325444	1.342694342862374E-4	909:40:00	476:06:00	06:57:00
SMART	SM00282:LamG	9	1.7751479289940828	1.4160311271268225E-4	863:35:00	64:44:00	245:17:00
INTERPRO	IPR000152:EGF-type aspartate/asparagine hy	11	2.1696252465483234	1.5383126466188957E-4	563:03:00	481:55:00	252:26:00
GOTERM_MF_FAT	GO:0030695~GTPase regulator activity	23	4.536489151873767	1.6151259639650775E-4	865:12:00	489:30:00	959:47:00
GOTERM_BP_FAT	GO:0031175~neuron projection development	17	3.353057199211045	1.7454846509913372E-4	233:30:00	650:41:00	203:08:00
GOTERM_CC_FAT	GO:0030054~cell junction	28	5.522682445759369	1.8646576533855964E-4	107:36:00	706:03:00	663:42:00
GOTERM_BP_FAT	GO:0006811~ion transport	36	7.100591715976331	1.933951643420524E-4	350:38:00	162:57:00	335:59:00
GOTERM_CC_FAT	GO:0042995~cell projection	32	6.31163708086785	1.9407864006446853E-4	712:02:00	272:30:00	608:47:00
GOTERM_MF_FAT	GO:0060589~nucleoside-triphosphatase regula	23	4.536489151873767	1.9923703046150962E-4	284:12:00	134:04:00	606:46:00
INTERPRO	IPR001828:Extracellular ligand-binding recepto	8	1.5779092702169626	2.1353906355970812E-4	872:03:00	354:37:00	622:19:00
GOTERM_BP_FAT	GO:0016310~phosphorylation	36	7.100591715976331	2.2597397246061187E-4	486:33:00	107:10:00	35:15:00
GOTERM_MF_FAT	GO:0004672~protein kinase activity	31	6.11439842209073	2.2979702464974284E-4	155:36:00	551:22:00	749:22:00
SP_PIR_KEYWORDS	cytoplasm	109	21.499013806706113	2.369909981732898E-4	574:26:00	142:12:00	242:59:00
SP_PIR_KEYWORDS	cell junction	24	4.733727810650888	2.494263110411946E-4	475:39:00	820:29:00	609:31:00
KEGG_PATHWAY	mmu04360:Axon guidance	12	2.366863905325444	3.003736776649717E-4	530:59:00	966:53:00	69:32:00
GOTERM_MF_FAT	GO:0004713~protein tyrosine kinase activity	14	2.7613412228796843	3.1303502140898627E-4	876:36:00	355:43:00	234:28:00
UP_TISSUE	Medulla oblongata	28	5.522682445759369	3.2371946687882383E-4	509:08:00	529:34:00	360:07:00
UP_TISSUE	Corpora quadrigemina	29	5.719921104536489	3.4078465847800206E-4	498:30:00	1084:50:00	16:34:00

KEGG_PATHWAY	mmu05414:Dilated cardiomyopathy	10	1.9723865877712032	3.496355134836699E-4	304:32:00	914:44:00	508:28:00
SMART	SM00060:FN3	18	3.5502958579881656	3.6585233001187325E-4	966:59:00	615:53:00	778:27:00
GOTERM_MF_FAT	GO:0008066~glutamate receptor activity	7	1.3806706114398422	3.769666446353763E-4	985:20:00	767:15:00	342:20:00
GOTERM_MF_FAT	GO:0015276~ligand-gated ion channel activity	11	2.1696252465483234	3.897717189580962E-4	597:13:00	737:34:00	1050:47:00
GOTERM_MF_FAT	GO:0022834~ligand-gated channel activity	11	2.1696252465483234	3.897717189580962E-4	597:13:00	737:34:00	1050:47:00
GOTERM_CC_FAT	GO:0014069~postsynaptic density	8	1.5779092702169626	4.1248218471140723E-4	558:56:00	744:02:00	686:27:00
SP_PIR_KEYWORDS	kinase	35	6.903353057199212	4.2105089925979176E-4	303:05:00	741:28:00	714:42:00
INTERPRO	IPR000719:Protein kinase, core	27	5.325443786982249	4.614843311645163E-4	1013:16:00	119:54:00	737:12:00
GOTERM_MF_FAT	GO:0001882~nucleoside binding	62	12.22879684418146	4.644201563309256E-4	1006:17:00	265:02:00	631:34:00
UP_SEQ_FEATURE	domain:EGF-like 6	7	1.3806706114398422	4.737372433381086E-4	982:15:00	1065:57:00	614:44:00
GOTERM_MF_FAT	GO:0030554~adenyl nucleotide binding	61	12.03155818540434	5.409840507024636E-4	216:41:00	289:22:00	01:08:00
GOTERM_MF_FAT	GO:0005524~ATP binding	58	11.439842209072978	5.968656373142921E-4	796:25:00	494:49:00	840:32:00
GOTERM_BP_FAT	GO:0048666~neuron development	19	3.7475345167652856	5.973985260171583E-4	353:34:00	69:47:00	1091:29:00
GOTERM_BP_FAT	GO:0046903~secretion	16	3.155818540433925	6.293309609414735E-4	754:21:00	393:39:00	673:05:00
UP_SEQ_FEATURE	domain:Laminin G-like 2	6	1.183431952662722	6.299170577548416E-4	419:33:00	517:21:00	673:22:00
UP_SEQ_FEATURE	domain:Laminin G-like 1	6	1.183431952662722	6.299170577548416E-4	419:33:00	517:21:00	673:22:00
UP_SEQ_FEATURE	domain:EGF-like 5	7	1.3806706114398422	6.347063921081768E-4	1003:05:00	1085:44:00	223:29:00
PIR_SUPERFAMILY	PIRSF002504:cadherin	5	0.9861932938856016	6.361023268411918E-4	783:52:00	968:53:00	968:53:00
UP_SEQ_FEATURE	domain:Ig-like C2-type 3	10	1.9723865877712032	6.420936443568575E-4	871:26:00	1023:12:00	241:50:00
GOTERM_BP_FAT	GO:0030030~cell projection organization	20	3.9447731755424065	6.456003807086591E-4	900:10:00	451:54:00	185:33:00
GOTERM_MF_FAT	GO:0001883~purine nucleoside binding	61	12.03155818540434	6.825443772591933E-4	374:52:00	639:34:00	1089:01:00
GOTERM_BP_FAT	GO:0030182~neuron differentiation	23	4.536489151873767	7.02371197046171E-4	201:29:00	324:39:00	285:36:00
GOTERM_BP_FAT	GO:0044057~regulation of system process	15	2.9585798816568047	7.397716872211619E-4	473:57:00	492:58:00	364:49:00
GOTERM_BP_FAT	GO:0007242~intracellular signaling cascade	41	8.086785009861932	7.427096023466387E-4	692:40:00	200:34:00	87:26:00
GOTERM_BP_FAT	GO:0048858~cell projection morphogenesis	15	2.9585798816568047	7.766972308070924E-4	901:04:00	18:15:00	142:01:00
GOTERM_MF_FAT	GO:0032559~adenyl ribonucleotide binding	58	11.439842209072978	7.896357107252106E-4	286:21:00	152:28:00	820:53:00
UP_SEQ_FEATURE	short sequence motif:PDZ-binding	9	1.7751479289940828	7.922936608443345E-4	832:47:00	280:21:00	880:26:00
GOTERM_BP_FAT	GO:0019226~transmission of nerve impulse	16	3.155818540433925	7.977148044870727E-4	254:55:00	41:57:00	57:32:00
GOTERM_BP_FAT	GO:0006303~double-strand break repair via nc	4	0.7889546351084813	8.689592768058796E-4	1085:19:00	412:54:00	665:06:00
GOTERM_BP_FAT	GO:0000726~non-recombinational repair	4	0.7889546351084813	8.689592768058796E-4	1085:19:00	412:54:00	665:06:00
KEGG_PATHWAY	mmu05410:Hypertrophic cardiomyopathy (HCM	9	1.7751479289940828	8.9572004135602E-4	188:34:00	1048:00:00	326:58:00
SP_PIR_KEYWORDS	signal	104	20.51282051282051	9.097979198293663E-4	878:54:00	963:44:00	735:27:00
GOTERM_BP_FAT	GO:0007411~axon guidance	10	1.9723865877712032	9.638440080093935E-4	129:53:00	237:14:00	837:36:00
SP_PIR_KEYWORDS	atp-binding	53	10.453648915187378	9.695440484593931E-4	25:03:00	86:59:00	388:50:00
GOTERM_MF_FAT	GO:0005234~extracellular-glutamate-gated ion	5	0.9861932938856016	9.804640813241765E-4	1087:07:00	123:38:00	99:39:00
GOTERM_MF_FAT	GO:0004970~ionotropic glutamate receptor act	5	0.9861932938856016	9.804640813241765E-4	1087:07:00	123:38:00	99:39:00
INTERPRO	IPR019594:Glutamate receptor, L-glutamate/gl	5	0.9861932938856016	494:26:00	361:09:00	788:31:00	1082:02:00
INTERPRO	IPR015683:Glutamate receptor-related	5	0.9861932938856016	494:26:00	361:09:00	788:31:00	1082:02:00
INTERPRO	IPR001320:Ionotropic glutamate receptor	5	0.9861932938856016	494:26:00	361:09:00	788:31:00	1082:02:00
INTERPRO	IPR001508:NMDA receptor	5	0.9861932938856016	494:26:00	361:09:00	788:31:00	1082:02:00
UP_SEQ_FEATURE	binding site:Glutamate	4	0.7889546351084813	293:54:00	640:50:00	307:52:00	426:05:00
UP_SEQ_FEATURE	region of interest:Glutamate binding	4	0.7889546351084813	293:54:00	640:50:00	307:52:00	426:05:00
INTERPRO	IPR017441:Protein kinase, ATP binding site	25	4.930966469428008	50:10:00	519:03:00	1064:02:00	983:42:00
GOTERM_BP_FAT	GO:0032990~cell part morphogenesis	15	2.9585798816568047	900:30:00	705:13:00	1050:23:00	725:20:00

GOTERM_BP_FAT	GO:0007243~protein kinase cascade	16	3.155818540433925	1015:16:00	272:59:00	107:33:00	148:47:00
GOTERM_MF_FAT	GO:0003779~actin binding	18	3.5502958579881656	360:31:00	323:17:00	316:46:00	314:40:00
SMART	SM00219:TyrKc	13	2.564102564102564	455:15:00	217:12:00	873:22:00	743:12:00
GOTERM_MF_FAT	GO:0043167~ion binding	128	25.2465483234714	864:51:00	84:21:00	812:42:00	800:44:00
GOTERM_MF_FAT	GO:0046873~metal ion transmembrane transp	18	3.5502958579881656	1021:39:00	821:25:00	415:37:00	1034:50:00
GOTERM_MF_FAT	GO:0019904~protein domain specific binding	14	2.7613412228796843	300:16:00	1028:12:00	409:24:00	1088:58:00
GOTERM_BP_FAT	GO:0003001~generation of a signal involved ir	9	1.7751479289940828	927:06:00	564:02:00	595:54:00	438:42:00
GOTERM_MF_FAT	GO:0005230~extracellular ligand-gated ion cha	8	1.5779092702169626	258:40:00	986:00:00	478:14:00	00:05:00
UP_TISSUE	Olfactory bulb	15	2.9585798816568047	260:19:00	993:57:00	636:12:00	123:12:00
GOTERM_CC_FAT	GO:0005856~cytoskeleton	48	9.467455621301776	195:58:00	68:17:00	1008:02:00	275:52:00
UP_SEQ_FEATURE	repeat:WD 6	13	2.564102564102564	960:35:00	02:34:00	425:28:00	713:11:00
GOTERM_MF_FAT	GO:0000166~nucleotide binding	78	15.384615384615385	237:33:00	603:27:00	610:51:00	64:53:00
GOTERM_MF_FAT	GO:0005262~calcium channel activity	8	1.5779092702169626	06:22:00	341:43:00	864:44:00	377:03:00
GOTERM_BP_FAT	GO:0051969~regulation of transmission of ner	10	1.9723865877712032	230:15:00	657:07:00	243:32:00	731:41:00
GOTERM_MF_FAT	GO:0005083~small GTPase regulator activity	15	2.9585798816568047	356:23:00	33:27:00	188:06:00	210:24:00
GOTERM_MF_FAT	GO:0005261~cation channel activity	16	3.155818540433925	524:00:00	635:53:00	71:17:00	616:42:00
UP_SEQ_FEATURE	domain:Cadherin 1	7	1.3806706114398422	852:55:00	502:35:00	1027:35:00	590:53:00
UP_SEQ_FEATURE	domain:Cadherin 2	7	1.3806706114398422	852:55:00	502:35:00	1027:35:00	590:53:00
GOTERM_BP_FAT	GO:0048812~neuron projection morphogenesi	13	2.564102564102564	855:57:00	954:11:00	494:42:00	806:25:00
UP_SEQ_FEATURE	transmembrane region	141	27.810650887573964	586:11:00	683:57:00	435:34:00	251:51:00
GOTERM_CC_FAT	GO:0019898~extrinsic to membrane	25	4.930966469428008	687:21:00	634:01:00	713:20:00	78:53:00
UP_SEQ_FEATURE	repeat:WD 7	11	2.1696252465483234	216:52:00	135:03:00	107:02:00	755:32:00
GOTERM_MF_FAT	GO:0043169~cation binding	125	24.65483234714004	269:25:00	616:26:00	511:32:00	172:14:00
PIR_SUPERFAMILY	PIRSF000666:TyrPK_ephrin_receptor	4	0.7889546351084813	610:12:00	593:33:00	314:05:00	922:12:00
GOTERM_MF_FAT	GO:0046872~metal ion binding	124	24.45759368836292	628:46:00	1077:34:00	61:23:00	732:52:00
INTERPRO	IPR000048:IQ calmodulin-binding region	9	1.7751479289940828	786:11:00	486:06:00	382:39:00	673:16:00
GOTERM_BP_FAT	GO:0031644~regulation of neurological systen	10	1.9723865877712032	362:01:00	707:25:00	538:24:00	125:59:00
GOTERM_BP_FAT	GO:0030534~adult behavior	9	1.7751479289940828	27:27:00	1039:52:00	485:23:00	156:37:00
GOTERM_BP_FAT	GO:0043087~regulation of GTPase activity	9	1.7751479289940828	27:27:00	1039:52:00	485:23:00	156:37:00
INTERPRO	IPR011042:Six-bladed beta-propeller, TolB-like	6	1.183431952662722	575:13:00	257:42:00	58:54:00	718:07:00
GOTERM_BP_FAT	GO:0000902~cell morphogenesis	18	3.5502958579881656	125:37:00	543:37:00	225:50:00	609:05:00
UP_SEQ_FEATURE	repeat:NHL 5	4	0.7889546351084813	89:13:00	1050:53:00	190:04:00	159:26:00
INTERPRO	IPR018097:EGF-like calcium-binding, conserve	9	1.7751479289940828	601:14:00	397:34:00	817:45:00	746:39:00
GOTERM_MF_FAT	GO:0004714~transmembrane receptor protein	7	1.3806706114398422	04:02:00	646:34:00	710:36:00	992:07:00
SMART	SM00079:PBPe	5	0.9861932938856016	572:33:00	1015:45:00	332:47:00	260:23:00
UP_SEQ_FEATURE	domain:Ig-like C2-type 1	11	2.1696252465483234	89:58:00	573:24:00	941:46:00	02:21:00
UP_SEQ_FEATURE	domain:IQ	7	1.3806706114398422	21:09:00	65:01:00	555:58:00	402:07:00
UP_SEQ_FEATURE	domain:Cadherin 5	6	1.183431952662722	1028:00:00	652:50:00	787:19:00	996:39:00
INTERPRO	IPR003599:Immunoglobulin subtype	18	3.5502958579881656	274:12:00	204:22:00	1013:53:00	704:27:00
GOTERM_BP_FAT	GO:0010648~negative regulation of cell comm	13	2.564102564102564	1077:08:00	833:48:00	523:09:00	287:47:00
GOTERM_BP_FAT	GO:0032940~secretion by cell	13	2.564102564102564	1077:08:00	833:48:00	523:09:00	287:47:00
INTERPRO	IPR001849:Pleckstrin homology	16	3.155818540433925	851:51:00	1063:49:00	485:16:00	433:48:00
GOTERM_BP_FAT	GO:0007156~homophilic cell adhesion	10	1.9723865877712032	808:30:00	613:37:00	1035:02:00	219:13:00
UP_SEQ_FEATURE	domain:Protein kinase	25	4.930966469428008	453:07:00	541:11:00	132:29:00	1053:48:00

UP_SEQ_FEATURE	domain:Ig-like C2-type 2	11	2.1696252465483234	81:04:00	88:11:00	242:39:00	785:17:00
GOTERM_BP_FAT	GO:0007409~axonogenesis	12	2.366863905325444	723:01:00	162:52:00	525:30:00	959:36:00
INTERPRO	IPR003598:Immunoglobulin subtype 2	13	2.564102564102564	724:00:00	638:46:00	568:35:00	340:59:00
UP_TISSUE	Hypothalamus	22	4.339250493096647	94:07:00	180:47:00	937:46:00	742:44:00
GOTERM_MF_FAT	GO:0030165~PDZ domain binding	5	0.9861932938856016	923:36:00	954:53:00	893:48:00	380:01:00
INTERPRO	IPR011993:Pleckstrin homology-type	17	3.353057199211045	394:23:00	23:33:00	607:07:00	186:04:00
UP_SEQ_FEATURE	repeat:NHL 1	4	0.7889546351084813	897:47:00	63:08:00	792:24:00	115:50:00
UP_SEQ_FEATURE	domain:Laminin G-like 4	4	0.7889546351084813	897:47:00	63:08:00	792:24:00	115:50:00
UP_SEQ_FEATURE	repeat:NHL 2	4	0.7889546351084813	897:47:00	63:08:00	792:24:00	115:50:00
UP_SEQ_FEATURE	repeat:NHL 4	4	0.7889546351084813	897:47:00	63:08:00	792:24:00	115:50:00
UP_SEQ_FEATURE	repeat:NHL 3	4	0.7889546351084813	897:47:00	63:08:00	792:24:00	115:50:00
INTERPRO	IPR009471:Teneurin intracellular, N-terminal	3	0.591715976331361	727:02:00	1011:15:00	118:43:00	270:16:00
INTERPRO	IPR006530:YD repeat	3	0.591715976331361	727:02:00	1011:15:00	118:43:00	270:16:00
UP_TISSUE	Fetal brain	18	3.5502958579881656	816:05:00	270:27:00	603:19:00	978:23:00
INTERPRO	IPR016257:Tyrosine-protein kinase, ephrin rec	4	0.7889546351084813	1039:27:00	247:49:00	404:33:00	260:01:00
INTERPRO	IPR000884:Thrombospondin, type 1 repeat	7	1.3806706114398422	175:44:00	854:14:00	174:46:00	85:44:00
UP_SEQ_FEATURE	domain:Fibronectin type-III 3	8	1.5779092702169626	500:58:00	1086:16:00	1046:52:00	282:07:00
GOTERM_BP_FAT	GO:0033554~cellular response to stress	21	4.142011834319527	720:25:00	302:37:00	258:52:00	205:51:00
UP_SEQ_FEATURE	compositionally biased region:Poly-Ser	21	4.142011834319527	929:27:00	862:47:00	621:50:00	953:55:00
GOTERM_BP_FAT	GO:0050804~regulation of synaptic transmissi	9	1.7751479289940828	802:18:00	862:45:00	737:54:00	407:10:00
UP_SEQ_FEATURE	repeat:YD 7	3	0.591715976331361	370:37:00	961:15:00	12:43:00	228:57:00
UP_SEQ_FEATURE	repeat:YD 8	3	0.591715976331361	370:37:00	961:15:00	12:43:00	228:57:00
UP_SEQ_FEATURE	repeat:YD 20	3	0.591715976331361	370:37:00	961:15:00	12:43:00	228:57:00
UP_SEQ_FEATURE	repeat:YD 14	3	0.591715976331361	370:37:00	961:15:00	12:43:00	228:57:00
UP_SEQ_FEATURE	repeat:YD 16	3	0.591715976331361	370:37:00	961:15:00	12:43:00	228:57:00
UP_SEQ_FEATURE	repeat:YD 13	3	0.591715976331361	370:37:00	961:15:00	12:43:00	228:57:00
UP_SEQ_FEATURE	repeat:YD 21	3	0.591715976331361	370:37:00	961:15:00	12:43:00	228:57:00
UP_SEQ_FEATURE	repeat:YD 4	3	0.591715976331361	370:37:00	961:15:00	12:43:00	228:57:00
UP_SEQ_FEATURE	repeat:YD 23	3	0.591715976331361	370:37:00	961:15:00	12:43:00	228:57:00
UP_SEQ_FEATURE	repeat:YD 1	3	0.591715976331361	370:37:00	961:15:00	12:43:00	228:57:00
UP_SEQ_FEATURE	domain:Teneurin N-terminal	3	0.591715976331361	370:37:00	961:15:00	12:43:00	228:57:00
UP_SEQ_FEATURE	repeat:YD 19	3	0.591715976331361	370:37:00	961:15:00	12:43:00	228:57:00
UP_SEQ_FEATURE	repeat:YD 2	3	0.591715976331361	370:37:00	961:15:00	12:43:00	228:57:00
UP_SEQ_FEATURE	repeat:YD 5	3	0.591715976331361	370:37:00	961:15:00	12:43:00	228:57:00
UP_SEQ_FEATURE	repeat:YD 6	3	0.591715976331361	370:37:00	961:15:00	12:43:00	228:57:00
UP_SEQ_FEATURE	repeat:YD 18	3	0.591715976331361	370:37:00	961:15:00	12:43:00	228:57:00
UP_SEQ_FEATURE	repeat:YD 9	3	0.591715976331361	370:37:00	961:15:00	12:43:00	228:57:00
UP_SEQ_FEATURE	repeat:YD 12	3	0.591715976331361	370:37:00	961:15:00	12:43:00	228:57:00
UP_SEQ_FEATURE	repeat:YD 15	3	0.591715976331361	370:37:00	961:15:00	12:43:00	228:57:00
UP_SEQ_FEATURE	repeat:YD 22	3	0.591715976331361	370:37:00	961:15:00	12:43:00	228:57:00
UP_SEQ_FEATURE	repeat:YD 3	3	0.591715976331361	370:37:00	961:15:00	12:43:00	228:57:00
UP_SEQ_FEATURE	repeat:YD 17	3	0.591715976331361	370:37:00	961:15:00	12:43:00	228:57:00
UP_SEQ_FEATURE	repeat:YD 11	3	0.591715976331361	370:37:00	961:15:00	12:43:00	228:57:00
UP_SEQ_FEATURE	repeat:YD 10	3	0.591715976331361	370:37:00	961:15:00	12:43:00	228:57:00

GOTERM_BP_FAT	GO:0032989~cellular component morphogene:	19	3.7475345167652856	456:11:00	94:41:00	03:37:00	92:41:00
UP_TISSUE	Head	59	11.637080867850099	802:13:00	884:13:00	254:33:00	716:31:00
KEGG_PATHWAY	mmu04010:MAPK signaling pathway	15	2.9585798816568047	975:34:00	1083:15:00	208:21:00	684:15:00
UP_SEQ_FEATURE	binding site:ATP	27	5.325443786982249	857:01:00	515:17:00	136:26:00	537:49:00
GOTERM_BP_FAT	GO:0006302~double-strand break repair	6	1.183431952662722	829:12:00	486:56:00	864:10:00	249:22:00
SP_PIR_KEYWORDS	DNA damage	13	2.564102564102564	193:41:00	535:03:00	956:58:00	1030:43:00
INTERPRO	IPR002126:Cadherin	9	1.7751479289940828	1060:41:00	35:20:00	424:50:00	185:27:00
GOTERM_BP_FAT	GO:0009968~negative regulation of signal tran	12	2.366863905325444	609:15:00	477:07:00	459:36:00	1056:05:00
GOTERM_MF_FAT	GO:0005003~ephrin receptor activity	4	0.7889546351084813	80:46:00	564:40:00	922:15:00	426:17:00
INTERPRO	IPR001090:Ephrin receptor, ligand binding	4	0.7889546351084813	580:47:00	899:37:00	716:16:00	764:03:00
INTERPRO	IPR001258:NHL repeat	4	0.7889546351084813	580:47:00	899:37:00	716:16:00	764:03:00
INTERPRO	IPR001426:Receptor tyrosine kinase, class V, c	4	0.7889546351084813	580:47:00	899:37:00	716:16:00	764:03:00
GOTERM_BP_FAT	GO:0008344~adult locomotory behavior	7	1.3806706114398422	800:58:00	600:42:00	134:10:00	973:29:00
GOTERM_BP_FAT	GO:0043065~positive regulation of apoptosis	15	2.9585798816568047	435:46:00	951:54:00	168:02:00	877:21:00
GOTERM_BP_FAT	GO:0043068~positive regulation of programme	15	2.9585798816568047	71:08:00	939:15:00	436:53:00	414:17:00
UP_SEQ_FEATURE	signal peptide	104	20.51282051282051	381:45:00	58:15:00	125:01:00	60:19:00
INTERPRO	IPR015943:WD40/YVTN repeat-like	17	3.353057199211045	1061:27:00	268:56:00	933:14:00	02:11:00
UP_TISSUE	Spinal cord	24	4.733727810650888	514:35:00	985:28:00	282:30:00	443:38:00
GOTERM_BP_FAT	GO:0010942~positive regulation of cell death	15	2.9585798816568047	321:13:00	406:41:00	67:09:00	712:55:00
GOTERM_MF_FAT	GO:0017076~purine nucleotide binding	66	13.017751479289942	625:42:00	620:16:00	143:07:00	529:57:00
UP_TISSUE	Ovary	18	3.5502958579881656	433:10:00	297:30:00	773:38:00	165:45:00
SP_PIR_KEYWORDS	actin-binding	14	2.7613412228796843	906:46:00	643:21:00	909:49:00	825:43:00
GOTERM_BP_FAT	GO:0033152~immunoglobulin V(D)J recombina	3	0.591715976331361	885:52:00	172:31:00	427:10:00	107:40:00
GOTERM_BP_FAT	GO:0051056~regulation of small GTPase medi	14	2.7613412228796843	660:46:00	611:52:00	489:51:00	545:14:00
GOTERM_CC_FAT	GO:0042734~presynaptic membrane	5	0.9861932938856016	189:22:00	873:21:00	1085:12:00	730:45:00
INTERPRO	IPR013151:Immunoglobulin	12	2.366863905325444	241:09:00	830:45:00	1003:09:00	41:38:00
UP_SEQ_FEATURE	nucleotide phosphate-binding region:ATP	39	7.6923076923076925	210:58:00	14:01:00	496:11:00	1026:21:00
UP_SEQ_FEATURE	zinc finger region:RING-type; atypical	5	0.9861932938856016	937:49:00	475:38:00	621:05:00	691:20:00
UP_SEQ_FEATURE	domain:Cadherin 4	6	1.183431952662722	1042:18:00	824:21:00	646:24:00	440:57:00
INTERPRO	IPR001660:Sterile alpha motif SAM	8	1.5779092702169626	443:44:00	1049:36:00	105:13:00	943:04:00
GOTERM_BP_FAT	GO:0046578~regulation of Ras protein signal t	12	2.366863905325444	1006:44:00	802:12:00	793:14:00	719:10:00
UP_SEQ_FEATURE	domain:Cadherin 3	6	1.183431952662722	41:48:00	826:36:00	792:22:00	929:45:00
GOTERM_BP_FAT	GO:0006887~exocytosis	9	1.7751479289940828	194:53:00	07:05:00	814:45:00	531:28:00
GOTERM_BP_FAT	GO:0048667~cell morphogenesis involved in n	12	2.366863905325444	1030:00:00	182:11:00	820:26:00	360:23:00
GOTERM_BP_FAT	GO:0006974~response to DNA damage stimul	16	3.155818540433925	394:09:00	1020:44:00	826:19:00	54:14:00
GOTERM_MF_FAT	GO:0032555~purine ribonucleotide binding	63	12.42603550295858	1067:03:00	649:21:00	1078:30:00	234:27:00
GOTERM_MF_FAT	GO:0032553~ribonucleotide binding	63	12.42603550295858	1067:03:00	649:21:00	1078:30:00	234:27:00
SP_PIR_KEYWORDS	coiled coil	62	12.22879684418146	915:08:00	796:49:00	629:32:00	293:53:00
GOTERM_CC_FAT	GO:0030424~axon	9	1.7751479289940828	1013:22:00	140:21:00	388:24:00	906:57:00
SP_PIR_KEYWORDS	nucleotide-binding	59	11.637080867850099	456:59:00	317:07:00	891:02:00	556:03:00
GOTERM_CC_FAT	GO:0019717~synaptosome	7	1.3806706114398422	711:32:00	699:25:00	514:13:00	745:31:00
INTERPRO	IPR018159:Spectrin/alpha-actinin	5	0.9861932938856016	09:33:00	1070:22:00	931:42:00	391:00:00
GOTERM_BP_FAT	GO:0001964~startle response	4	0.7889546351084813	599:12:00	729:13:00	855:56:00	783:02:00
INTERPRO	IPR001881:EGF-like calcium-binding	8	1.5779092702169626	539:14:00	193:47:00	342:57:00	766:53:00

GOTERM_MF_FAT	GO:0000287~magnesium ion binding	20	3.9447731755424065	481:42:00	18:05:00	656:00:00	198:11:00
GOTERM_BP_FAT	GO:0007626~locomotory behavior	14	2.7613412228796843	840:22:00	1002:36:00	859:10:00	543:54:00
SP_PIR_KEYWORDS	calcium transport	7	1.3806706114398422	391:22:00	96:15:00	497:20:00	948:11:00
GOTERM_BP_FAT	GO:0016192~vesicle-mediated transport	22	4.339250493096647	608:20:00	228:08:00	860:24:00	1061:03:00
UP_TISSUE	Spinal ganglion	19	3.7475345167652856	64:23:00	255:09:00	1016:45:00	734:18:00
INTERPRO	IPR001202:WW/Rsp5/WWP	6	1.183431952662722	278:22:00	47:06:00	1057:37:00	944:19:00
SP_PIR_KEYWORDS	disulfide bond	83	16.370808678500985	975:39:00	900:34:00	766:27:00	866:39:00
UP_SEQ_FEATURE	repeat:WD 5	13	2.564102564102564	458:29:00	696:11:00	860:56:00	151:22:00
UP_SEQ_FEATURE	domain:F5/8 type C 2	3	0.591715976331361	994:27:00	640:50:00	861:15:00	36:35:00
UP_SEQ_FEATURE	domain:F5/8 type C 1	3	0.591715976331361	994:27:00	640:50:00	861:15:00	36:35:00
UP_SEQ_FEATURE	domain:Laminin G-like 3	4	0.7889546351084813	738:30:00	1098:15:00	861:17:00	466:23:00
INTERPRO	IPR013761:Sterile alpha motif-type	7	1.3806706114398422	270:57:00	939:08:00	298:07:00	601:13:00
GOTERM_BP_FAT	GO:0043067~regulation of programmed cell de	25	4.930966469428008	328:17:00	1008:30:00	861:33:00	792:43:00
GOTERM_BP_FAT	GO:0007010~cytoskeleton organization	17	3.353057199211045	127:14:00	443:39:00	861:38:00	843:11:00
GOTERM_BP_FAT	GO:0030001~metal ion transport	21	4.142011834319527	742:51:00	559:15:00	861:40:00	771:07:00
UP_SEQ_FEATURE	domain:SAM	7	1.3806706114398422	766:17:00	296:31:00	861:32:00	282:04:00
SP_PIR_KEYWORDS	calcium channel	6	1.183431952662722	51:43:00	151:38:00	987:35:00	532:38:00
GOTERM_BP_FAT	GO:0051656~establishment of organelle localiz	5	0.9861932938856016	1054:55:00	247:20:00	861:42:00	1071:59:00
UP_SEQ_FEATURE	domain:EGF-like 4; calcium-binding	5	0.9861932938856016	119:51:00	767:55:00	861:36:00	228:21:00
GOTERM_BP_FAT	GO:0010941~regulation of cell death	25	4.930966469428008	1038:52:00	878:15:00	861:46:00	611:40:00
SMART	SM00615:EPH_lbd	4	0.7889546351084813	42:08:00	992:18:00	812:40:00	787:50:00
GOTERM_BP_FAT	GO:0007167~enzyme linked receptor protein s	15	2.9585798816568047	463:20:00	815:12:00	861:48:00	542:23:00
UP_SEQ_FEATURE	repeat:WD 3	14	2.7613412228796843	634:49:00	883:14:00	861:47:00	467:36:00
UP_SEQ_FEATURE	domain:EGF-like 4	6	1.183431952662722	441:05:00	399:36:00	861:47:00	647:48:00
UP_TISSUE	In vitro fertilized eggs	13	2.564102564102564	358:11:00	266:24:00	474:07:00	745:18:00
INTERPRO	IPR003604:Zinc finger, U1-type	4	0.7889546351084813	973:47:00	707:41:00	430:39:00	928:10:00
GOTERM_BP_FAT	GO:0042417~dopamine metabolic process	4	0.7889546351084813	595:58:00	301:11:00	861:50:00	534:11:00
COG_ONTOLOGY	Cell envelope biogenesis, outer membrane	4	0.7889546351084813	887:22:00	335:55:00	623:42:00	623:42:00
UP_TISSUE	Visual cortex	13	2.564102564102564	251:19:00	913:57:00	71:14:00	207:40:00
UP_TISSUE	Eye	52	10.256410256410255	275:28:00	523:13:00	183:01:00	590:06:00
GOTERM_BP_FAT	GO:0007269~neurotransmitter secretion	5	0.9861932938856016	728:12:00	1026:03:00	861:51:00	660:15:00
UP_SEQ_FEATURE	repeat:WD 1	14	2.7613412228796843	319:37:00	309:54:00	861:51:00	78:30:00
UP_SEQ_FEATURE	repeat:WD 2	14	2.7613412228796843	319:37:00	309:54:00	861:51:00	78:30:00
SP_PIR_KEYWORDS	cell projection	12	2.366863905325444	202:20:00	475:39:00	720:44:00	898:36:00
SP_PIR_KEYWORDS	transport	56	11.045364891518737	176:40:00	283:09:00	12:52:00	924:16:00
GOTERM_MF_FAT	GO:0005548~phospholipid transporter activity	4	0.7889546351084813	137:01:00	954:53:00	1045:57:00	267:18:00
GOTERM_BP_FAT	GO:0006812~cation transport	23	4.536489151873767	497:22:00	147:43:00	861:51:00	562:12:00
SP_PIR_KEYWORDS	motor protein	9	1.7751479289940828	212:51:00	531:27:00	99:01:00	1025:10:00
SMART	SM00015:IQ	9	1.7751479289940828	919:41:00	646:33:00	934:55:00	477:07:00
GOTERM_BP_FAT	GO:0006928~cell motion	18	3.5502958579881656	317:28:00	730:59:00	861:51:00	877:36:00
UP_TISSUE	Retina	26	5.128205128205128	280:11:00	103:06:00	181:41:00	617:12:00
SP_PIR_KEYWORDS	dna repair	11	2.1696252465483234	1089:39:00	739:13:00	111:22:00	620:20:00
INTERPRO	IPR008266:Tyrosine protein kinase, active site	8	1.5779092702169626	562:52:00	210:59:00	841:27:00	953:48:00
GOTERM_BP_FAT	GO:0033044~regulation of chromosome organ	4	0.7889546351084813	350:48:00	566:24:00	861:51:00	35:10:00

SMART	SM00209:TSP1	7	1.3806706114398422	1020:29:00	288:06:00	240:38:00	398:19:00
INTERPRO	IPR019775:WD40 repeat, conserved site	14	2.7613412228796843	580:49:00	414:08:00	854:40:00	904:15:00
GOTERM_BP_FAT	GO:0042981~regulation of apoptosis	24	4.733727810650888	628:58:00	696:31:00	861:51:00	472:00:00
GOTERM_BP_FAT	GO:0006836~neurotransmitter transport	7	1.3806706114398422	604:59:00	251:15:00	861:51:00	740:46:00
GOTERM_BP_FAT	GO:0032318~regulation of Ras GTPase activit	7	1.3806706114398422	604:59:00	251:15:00	861:51:00	740:46:00
INTERPRO	IPR001680:WD40 repeat	14	2.7613412228796843	563:56:00	219:28:00	857:08:00	666:30:00
GOTERM_BP_FAT	GO:0016358~dendrite development	5	0.9861932938856016	16:00:00	804:44:00	861:51:00	291:40:00
GOTERM_MF_FAT	GO:0003712~transcription cofactor activity	11	2.1696252465483234	87:46:00	957:28:00	747:24:00	230:20:00
UP_SEQ_FEATURE	repeat:WD 4	13	2.564102564102564	956:01:00	178:59:00	861:51:00	195:49:00
INTERPRO	IPR002017:Spectrin repeat	4	0.7889546351084813	709:15:00	572:29:00	859:48:00	748:43:00
SP_PIR_KEYWORDS	ubl conjugation	23	4.536489151873767	626:12:00	419:11:00	476:58:00	407:14:00
PIR_SUPERFAMILY	PIRSF002437:ionotropic glutamate receptor, AI	3	0.591715976331361	585:18:00	949:12:00	963:35:00	385:20:00
UP_SEQ_FEATURE	domain:Fibronectin type-III 4	6	1.183431952662722	978:11:00	146:32:00	861:51:00	750:30:00
UP_SEQ_FEATURE	domain:Ig-like C2-type 4	6	1.183431952662722	978:11:00	146:32:00	861:51:00	750:30:00
GOTERM_MF_FAT	GO:0008509~anion transmembrane transporte	9	1.7751479289940828	468:18:00	778:46:00	575:50:00	22:20:00
UP_SEQ_FEATURE	compositionally biased region:Poly-Ala	15	2.9585798816568047	917:06:00	202:36:00	861:51:00	602:51:00
INTERPRO	IPR000008:C2 calcium-dependent membrane t	9	1.7751479289940828	552:19:00	601:12:00	861:11:00	333:09:00
SP_PIR_KEYWORDS	neurotransmitter receptor	5	0.9861932938856016	885:07:00	724:18:00	33:35:00	267:50:00
INTERPRO	IPR018029:C2 membrane targeting protein	8	1.5779092702169626	264:01:00	929:41:00	861:22:00	16:17:00
UP_SEQ_FEATURE	domain:C2 2	6	1.183431952662722	492:35:00	558:33:00	861:51:00	268:48:00
UP_SEQ_FEATURE	domain:C2 1	6	1.183431952662722	492:35:00	558:33:00	861:51:00	268:48:00
SP_PIR_KEYWORDS	wd repeat	14	2.7613412228796843	234:51:00	343:05:00	515:25:00	715:58:00
GOTERM_MF_FAT	GO:0016564~transcription repressor activity	12	2.366863905325444	27:41:00	979:56:00	241:08:00	1060:26:00
INTERPRO	IPR002290:Serine/threonine protein kinase	14	2.7613412228796843	657:50:00	232:19:00	861:34:00	734:06:00
INTERPRO	IPR000233:Cadherin cytoplasmic region	4	0.7889546351084813	189:16:00	897:05:00	861:36:00	882:12:00
GOTERM_CC_FAT	GO:0030864~cortical actin cytoskeleton	4	0.7889546351084813	609:15:00	149:03:00	786:25:00	1089:04:00
GOTERM_BP_FAT	GO:0015914~phospholipid transport	4	0.7889546351084813	877:37:00	236:39:00	861:51:00	767:00:00
UP_TISSUE	Aorta and vein	11	2.1696252465483234	1004:03:00	406:58:00	152:34:00	762:52:00
GOTERM_BP_FAT	GO:0033151~V(D)J recombination	3	0.591715976331361	980:49:00	933:22:00	861:51:00	463:18:00
GOTERM_BP_FAT	GO:0007017~microtubule-based process	12	2.366863905325444	23:46:00	149:57:00	861:51:00	829:17:00
GOTERM_BP_FAT	GO:0001505~regulation of neurotransmitter lev	6	1.183431952662722	541:45:00	1039:52:00	861:51:00	656:57:00
GOTERM_BP_FAT	GO:0000904~cell morphogenesis involved in d	12	2.366863905325444	886:32:00	1088:05:00	861:51:00	1086:25:00
GOTERM_MF_FAT	GO:0005544~calcium-dependent phospholipid	4	0.7889546351084813	909:05:00	652:16:00	668:55:00	384:33:00
SP_PIR_KEYWORDS	Immunoglobulin domain	20	3.9447731755424065	437:48:00	452:06:00	798:13:00	190:16:00
UP_SEQ_FEATURE	compositionally biased region:Gln-rich	9	1.7751479289940828	997:03:00	97:41:00	01:00:00	445:13:00
GOTERM_CC_FAT	GO:0000922~spindle pole	4	0.7889546351084813	96:17:00	1057:45:00	881:54:00	373:25:00
SP_PIR_KEYWORDS	serine/threonine-protein kinase	18	3.5502958579881656	848:23:00	155:54:00	423:22:00	209:48:00
SMART	SM00150:SPEC	5	0.9861932938856016	730:43:00	107:57:00	259:06:00	462:34:00
UP_SEQ_FEATURE	domain:TSP type-1 4	4	0.7889546351084813	866:16:00	1069:19:00	01:00:00	846:48:00
UP_SEQ_FEATURE	domain:EGF-like 9	4	0.7889546351084813	866:16:00	1069:19:00	01:00:00	846:48:00
UP_SEQ_FEATURE	domain:PH	12	2.366863905325444	375:51:00	210:50:00	01:00:00	301:51:00
GOTERM_MF_FAT	GO:0004674~protein serine/threonine kinase a	19	3.7475345167652856	831:01:00	493:52:00	780:05:00	126:50:00
INTERPRO	IPR008271:Serine/threonine protein kinase, ac	17	3.353057199211045	56:48:00	194:19:00	861:51:00	1054:26:00
INTERPRO	IPR011500:GPCR, family 3, nine cysteines reg	4	0.7889546351084813	840:30:00	1008:48:00	861:51:00	988:33:00

GOTERM_MF_FAT	GO:0003774~motor activity	9	1.7751479289940828	112:48:00	364:42:00	822:31:00	467:14:00
SMART	SM00112:CA	9	1.7751479289940828	139:11:00	78:10:00	796:38:00	415:26:00
UP_TISSUE	Pituitary	12	2.366863905325444	170:09:00	612:57:00	483:19:00	584:35:00
GOTERM_MF_FAT	GO:0005096~GTPase activator activity	11	2.1696252465483234	773:57:00	516:57:00	833:20:00	799:27:00
KEGG_PATHWAY	mmu04720:Long-term potentiation	6	1.183431952662722	295:14:00	759:56:00	533:14:00	697:23:00
SMART	SM00451:ZnF_U1	4	0.7889546351084813	96:50:00	600:58:00	840:30:00	880:25:00
GOTERM_MF_FAT	GO:0005516~calmodulin binding	8	1.5779092702169626	289:27:00	842:42:00	844:03:00	1051:54:00
GOTERM_BP_FAT	GO:0007169~transmembrane receptor protein	11	2.1696252465483234	998:16:00	929:36:00	01:00:00	129:36:00
SP_PIR_KEYWORDS	transmembrane	156	30.76923076923077	954:20:00	17:52:00	824:01:00	21:28:00
INTERPRO	IPR001589:Actinin-type, actin-binding, conserv	4	0.7889546351084813	895:27:00	08:12:00	861:51:00	263:21:00
INTERPRO	IPR006652:Kelch repeat type 1	6	1.183431952662722	196:20:00	759:06:00	861:51:00	962:59:00
INTERPRO	IPR015915:Kelch-type beta propeller	6	1.183431952662722	196:20:00	759:06:00	861:51:00	962:59:00
GOTERM_CC_FAT	GO:0044430~cytoskeletal part	31	6.11439842209073	435:23:00	96:41:00	500:05:00	1038:01:00
GOTERM_BP_FAT	GO:0006281~DNA repair	12	2.366863905325444	578:12:00	587:26:00	01:00:00	754:16:00
KEGG_PATHWAY	mmu04730:Long-term depression	6	1.183431952662722	498:10:00	727:46:00	122:47:00	163:09:00
INTERPRO	IPR002172:Low density lipoprotein-receptor, cl	5	0.9861932938856016	366:40:00	961:06:00	861:51:00	326:59:00
SMART	SM00408:IGc2	13	2.564102564102564	102:02:00	722:44:00	335:31:00	842:09:00
GOTERM_MF_FAT	GO:0045296~cadherin binding	3	0.591715976331361	801:06:00	811:32:00	858:55:00	594:03:00
GOTERM_BP_FAT	GO:0051336~regulation of hydrolase activity	11	2.1696252465483234	328:22:00	960:08:00	01:00:00	302:24:00
GOTERM_BP_FAT	GO:0021952~central nervous system projectio	3	0.591715976331361	869:17:00	11:21:00	01:00:00	278:02:00
SP_PIR_KEYWORDS	tyrosine-protein kinase	8	1.5779092702169626	792:50:00	502:23:00	220:44:00	341:17:00
INTERPRO	IPR000569:HECT	4	0.7889546351084813	765:31:00	333:56:00	861:51:00	306:33:00
SP_PIR_KEYWORDS	kelch repeat	6	1.183431952662722	940:07:00	680:36:00	231:51:00	498:55:00
GOTERM_BP_FAT	GO:0048168~regulation of neuronal synaptic p	4	0.7889546351084813	970:13:00	944:30:00	01:00:00	141:03:00
GOTERM_BP_FAT	GO:0007628~adult walking behavior	4	0.7889546351084813	970:13:00	944:30:00	01:00:00	141:03:00
SMART	SM00456:WW	6	1.183431952662722	1050:06:00	181:22:00	385:23:00	253:49:00
SMART	SM00454:SAM	8	1.5779092702169626	265:08:00	225:10:00	148:12:00	349:16:00
UP_SEQ_FEATURE	repeat:Kelch 6	5	0.9861932938856016	251:47:00	730:28:00	01:00:00	712:55:00
GOTERM_BP_FAT	GO:0015833~peptide transport	5	0.9861932938856016	166:26:00	710:38:00	01:00:00	46:18:00
GOTERM_BP_FAT	GO:0007610~behavior	18	3.5502958579881656	61:05:00	58:25:00	01:00:00	66:00:00
SP_PIR_KEYWORDS	magnesium	18	3.5502958579881656	71:34:00	636:29:00	625:40:00	450:31:00
GOTERM_BP_FAT	GO:0001764~neuron migration	6	1.183431952662722	715:18:00	438:37:00	01:00:00	420:49:00
INTERPRO	IPR002181:Fibrinogen, alpha/beta/gamma cha	4	0.7889546351084813	289:08:00	1026:29:00	861:51:00	653:51:00
GOTERM_BP_FAT	GO:0007270~nerve-nerve synaptic transmissic	4	0.7889546351084813	148:21:00	366:57:00	01:00:00	476:49:00
GOTERM_BP_FAT	GO:0010212~response to ionizing radiation	5	0.9861932938856016	548:52:00	423:35:00	01:00:00	1057:14:00
GOTERM_BP_FAT	GO:0048167~regulation of synaptic plasticity	5	0.9861932938856016	548:52:00	423:35:00	01:00:00	1057:14:00
GOTERM_MF_FAT	GO:0005089~Rho guanyl-nucleotide exchange	6	1.183431952662722	549:36:00	705:33:00	861:34:00	308:58:00
PIR_SUPERFAMILY	PIRSF005608:metabotropic glutamate recepto	3	0.591715976331361	670:07:00	143:46:00	298:15:00	519:16:00
GOTERM_MF_FAT	GO:0005246~calcium channel regulator activity	3	0.591715976331361	887:35:00	432:16:00	861:41:00	846:34:00
INTERPRO	IPR001862:Membrane attack complex compon	3	0.591715976331361	106:02:00	215:49:00	861:51:00	89:36:00
GOTERM_BP_FAT	GO:0044065~regulation of respiratory system	3	0.591715976331361	755:09:00	973:05:00	01:00:00	420:29:00
GOTERM_BP_FAT	GO:0002087~regulation of respiratory gaseous	3	0.591715976331361	755:09:00	973:05:00	01:00:00	420:29:00
UP_SEQ_FEATURE	domain:Fibrinogen C-terminal	4	0.7889546351084813	424:05:00	804:17:00	01:00:00	06:31:00
UP_SEQ_FEATURE	domain:TSP type-1 3	4	0.7889546351084813	424:05:00	804:17:00	01:00:00	06:31:00

GOTERM_MF_FAT	GO:0003714~transcription corepressor activity	6	1.183431952662722	373:58:00	872:16:00	861:45:00	649:09:00
GOTERM_BP_FAT	GO:0006816~calcium ion transport	8	1.5779092702169626	1023:30:00	169:22:00	01:00:00	789:15:00
GOTERM_BP_FAT	GO:0048489~synaptic vesicle transport	4	0.7889546351084813	1041:55:00	808:30:00	01:00:00	953:43:00
GOTERM_BP_FAT	GO:0007613~memory	4	0.7889546351084813	1041:55:00	808:30:00	01:00:00	953:43:00
KEGG_PATHWAY	mmu03450:Non-homologous end-joining	3	0.591715976331361	761:03:00	243:06:00	545:45:00	995:14:00
GOTERM_MF_FAT	GO:0003777~microtubule motor activity	6	1.183431952662722	363:43:00	142:38:00	861:49:00	1055:31:00
SMART	SM00179:EGF_CA	8	1.5779092702169626	98:02:00	379:39:00	823:59:00	551:36:00
INTERPRO	IPR011705:BTB/Kelch-associated	5	0.9861932938856016	541:26:00	165:56:00	861:51:00	585:37:00
GOTERM_BP_FAT	GO:0022604~regulation of cell morphogenesis	7	1.3806706114398422	334:07:00	333:57:00	01:00:00	758:28:00
COG_ONTOLOGY	Signal transduction mechanisms	3	0.591715976331361	849:07:00	776:23:00	238:59:00	1005:04:00
UP_SEQ_FEATURE	repeat:WD 8	5	0.9861932938856016	78:07:00	940:34:00	01:00:00	137:40:00
SMART	SM00233:PH	16	3.155818540433925	808:53:00	437:04:00	376:48:00	230:45:00
UP_SEQ_FEATURE	domain:WW 2	4	0.7889546351084813	1031:42:00	946:04:00	01:00:00	643:29:00
UP_SEQ_FEATURE	domain:WW 1	4	0.7889546351084813	1031:42:00	946:04:00	01:00:00	643:29:00
GOTERM_CC_FAT	GO:0044463~cell projection part	10	1.9723865877712032	491:51:00	728:51:00	830:47:00	316:09:00
GOTERM_MF_FAT	GO:0005244~voltage-gated ion channel activity	10	1.9723865877712032	679:59:00	19:00:00	861:51:00	838:48:00
GOTERM_MF_FAT	GO:0022832~voltage-gated channel activity	10	1.9723865877712032	679:59:00	19:00:00	861:51:00	838:48:00
GOTERM_BP_FAT	GO:0018958~phenol metabolic process	4	0.7889546351084813	120:23:00	874:29:00	01:00:00	945:04:00
GOTERM_BP_FAT	GO:0006584~catecholamine metabolic process	4	0.7889546351084813	120:23:00	874:29:00	01:00:00	945:04:00
GOTERM_BP_FAT	GO:0034311~diol metabolic process	4	0.7889546351084813	120:23:00	874:29:00	01:00:00	945:04:00
GOTERM_BP_FAT	GO:0009712~catechol metabolic process	4	0.7889546351084813	120:23:00	874:29:00	01:00:00	945:04:00
UP_SEQ_FEATURE	domain:Ig-like C2-type 5	5	0.9861932938856016	438:43:00	70:43:00	01:00:00	939:54:00
PIR_SUPERFAMILY	PIRSF036344:neurexin	2	0.3944773175542406	690:27:00	636:58:00	726:28:00	78:49:00
PIR_SUPERFAMILY	PIRSF500998:transient receptor potential catio	2	0.3944773175542406	690:27:00	636:58:00	726:28:00	78:49:00
INTERPRO	IPR014847:FERM adjacent (FA)	3	0.591715976331361	543:19:00	128:01:00	01:00:00	379:44:00
GOTERM_MF_FAT	GO:0016791~phosphatase activity	12	2.366863905325444	351:21:00	449:17:00	861:51:00	893:46:00
GOTERM_BP_FAT	GO:0080135~regulation of cellular response to	6	1.183431952662722	909:00:00	524:41:00	01:00:00	684:43:00
KEGG_PATHWAY	mmu04530:Tight junction	8	1.5779092702169626	17:35:00	777:56:00	83:02:00	131:58:00
SMART	SM00409:IG	18	3.5502958579881656	709:13:00	207:32:00	1055:10:00	863:07:00
GOTERM_CC_FAT	GO:0044448~cell cortex part	6	1.183431952662722	440:54:00	998:16:00	857:22:00	955:53:00
UP_SEQ_FEATURE	domain:MAM	3	0.591715976331361	683:53:00	419:33:00	01:00:00	127:05:00
INTERPRO	IPR000203:GPS	4	0.7889546351084813	1035:22:00	1084:28:00	01:00:00	442:08:00
INTERPRO	IPR003585:Neurexin/syndecan/glycophorin C	3	0.591715976331361	587:47:00	563:10:00	01:00:00	1000:30:00
SP_PIR_KEYWORDS	transferase	47	9.270216962524655	789:53:00	690:56:00	860:11:00	366:55:00
KEGG_PATHWAY	mmu04080:Neuroactive ligand-receptor interac	12	2.366863905325444	178:47:00	518:51:00	1044:50:00	243:44:00
GOTERM_BP_FAT	GO:0051640~organelle localization	5	0.9861932938856016	88:27:00	970:59:00	01:00:00	106:27:00
INTERPRO	IPR005121:Phenylalanyl-tRNA synthetase, bet	2	0.3944773175542406	442:56:00	233:49:00	01:00:00	316:11:00
UP_TISSUE	Hippocampus	36	7.100591715976331	894:00:00	201:02:00	1061:53:00	549:52:00
SP_PIR_KEYWORDS	calmodulin-binding	7	1.3806706114398422	303:21:00	841:10:00	861:02:00	675:06:00
GOTERM_CC_FAT	GO:0015629~actin cytoskeleton	11	2.1696252465483234	1057:09:00	323:51:00	860:53:00	96:56:00
INTERPRO	IPR003962:Fibronectin, type III subdomain	4	0.7889546351084813	336:18:00	252:11:00	01:00:00	368:23:00
UP_SEQ_FEATURE	domain:GPS	4	0.7889546351084813	1019:47:00	1005:58:00	01:00:00	537:55:00
GOTERM_CC_FAT	GO:0048787~presynaptic active zone membra	2	0.3944773175542406	152:08:00	421:44:00	861:14:00	23:02:00
GOTERM_CC_FAT	GO:0008328~ionotropic glutamate receptor co	3	0.591715976331361	875:56:00	783:17:00	861:16:00	399:43:00

GOTERM_BP_FAT	GO:0060284~regulation of cell development	9	1.7751479289940828	754:55:00	1088:05:00	01:00:00	260:38:00
UP_SEQ_FEATURE	domain:FDX-ACB	2	0.3944773175542406	22:50:00	1034:22:00	01:00:00	893:53:00
UP_SEQ_FEATURE	region of interest:DACHbox-C	2	0.3944773175542406	22:50:00	1034:22:00	01:00:00	893:53:00
UP_SEQ_FEATURE	region of interest:DACHbox-N	2	0.3944773175542406	22:50:00	1034:22:00	01:00:00	893:53:00
UP_SEQ_FEATURE	region of interest:Essential for binding to NHEF	2	0.3944773175542406	22:50:00	1034:22:00	01:00:00	893:53:00
UP_SEQ_FEATURE	disulfide bond	79	15.581854043392504	1071:02:00	471:42:00	01:00:00	97:57:00
UP_SEQ_FEATURE	repeat:Kelch 5	5	0.9861932938856016	759:14:00	370:45:00	01:00:00	337:31:00
UP_SEQ_FEATURE	domain:F5/8 type C	3	0.591715976331361	1041:43:00	524:28:00	01:00:00	776:58:00
INTERPRO	IPR006539:ATPase, P-type, phospholipid-trans	3	0.591715976331361	14:52:00	563:17:00	01:00:00	1038:03:00
INTERPRO	IPR012675:Beta-grasp fold, ferredoxin-type	3	0.591715976331361	14:52:00	563:17:00	01:00:00	1038:03:00
GOTERM_BP_FAT	GO:0043523~regulation of neuron apoptosis	6	1.183431952662722	885:00:00	239:10:00	01:00:00	461:28:00
GOTERM_BP_FAT	GO:0021955~central nervous system neuron a	3	0.591715976331361	897:29:00	295:17:00	01:00:00	367:32:00
GOTERM_BP_FAT	GO:0031056~regulation of histone modificatior	3	0.591715976331361	897:29:00	295:17:00	01:00:00	367:32:00
GOTERM_BP_FAT	GO:0007015~actin filament organization	5	0.9861932938856016	94:23:00	63:53:00	01:00:00	643:39:00
KEGG_PATHWAY	mmu04540:Gap junction	6	1.183431952662722	1068:56:00	490:54:00	509:25:00	191:48:00
GOTERM_MF_FAT	GO:0008047~enzyme activator activity	12	2.366863905325444	196:01:00	952:53:00	861:51:00	350:04:00
GOTERM_CC_FAT	GO:0005654~nucleoplasm	24	4.733727810650888	05:15:00	640:16:00	861:42:00	261:19:00
GOTERM_BP_FAT	GO:0015674~di-, tri-valent inorganic cation trai	9	1.7751479289940828	51:57:00	283:48:00	01:00:00	373:55:00
GOTERM_MF_FAT	GO:0017016~Ras GTPase binding	5	0.9861932938856016	873:27:00	76:48:00	861:51:00	132:28:00
UP_TISSUE	Embryonic body below diaphragm region	3	0.591715976331361	55:40:00	314:44:00	718:39:00	233:06:00
GOTERM_BP_FAT	GO:0042770~DNA damage response, signal tr	5	0.9861932938856016	847:37:00	439:02:00	01:00:00	125:46:00
SP_PIR_KEYWORDS	sh3-binding	5	0.9861932938856016	27:14:00	725:24:00	861:49:00	1085:01:00
GOTERM_MF_FAT	GO:0016879~ligase activity, forming carbon-nit	10	1.9723865877712032	928:46:00	870:46:00	861:51:00	978:36:00
GOTERM_MF_FAT	GO:0008289~lipid binding	15	2.9585798816568047	1006:53:00	894:11:00	861:51:00	64:26:00
UP_SEQ_FEATURE	domain:Rab-GAP TBC	4	0.7889546351084813	442:21:00	835:36:00	01:00:00	327:04:00
GOTERM_MF_FAT	GO:0005088~Ras guanyl-nucleotide exchange	6	1.183431952662722	348:59:00	998:54:00	861:51:00	292:58:00
SP_PIR_KEYWORDS	voltage-gated channel	8	1.5779092702169626	35:07:00	634:22:00	861:50:00	678:54:00
GOTERM_MF_FAT	GO:0004012~phospholipid-translocating ATPa	3	0.591715976331361	487:54:00	108:59:00	861:51:00	512:37:00
GOTERM_MF_FAT	GO:0050839~cell adhesion molecule binding	3	0.591715976331361	487:54:00	108:59:00	861:51:00	512:37:00
GOTERM_MF_FAT	GO:0015247~aminophospholipid transporter a	3	0.591715976331361	487:54:00	108:59:00	861:51:00	512:37:00
UP_SEQ_FEATURE	domain:FAD-binding FR-type	3	0.591715976331361	209:09:00	379:02:00	01:00:00	870:31:00
UP_SEQ_FEATURE	domain:TSP type-1 6	3	0.591715976331361	209:09:00	379:02:00	01:00:00	870:31:00
GOTERM_BP_FAT	GO:0043576~regulation of respiratory gaseous	3	0.591715976331361	675:00:00	514:36:00	01:00:00	133:03:00
GOTERM_BP_FAT	GO:0007416~synaptogenesis	3	0.591715976331361	675:00:00	514:36:00	01:00:00	133:03:00
UP_TISSUE	Mammary tumor. Metallothionien-TGF alpha m	14	2.7613412228796843	765:53:00	379:13:00	290:33:00	136:50:00
SP_PIR_KEYWORDS	chromatin regulator	10	1.9723865877712032	404:20:00	557:46:00	861:51:00	1054:47:00
PIR_SUPERFAMILY	PIRSF037625:HERC protein	2	0.3944773175542406	1044:38:00	788:44:00	834:31:00	679:40:00
SMART	SM00457:MACPF	3	0.591715976331361	865:35:00	868:15:00	837:29:00	433:44:00
PIR_TISSUE_SPECIF	Expressed in testis.	3	0.591715976331361	774:27:00	1089:34:00	375:25:00	375:25:00
GOTERM_MF_FAT	GO:0016881~acid-amino acid ligase activity	9	1.7751479289940828	13:14:00	438:47:00	861:51:00	1087:39:00
UP_SEQ_FEATURE	repeat:Kelch 4	5	0.9861932938856016	1061:57:00	626:42:00	01:00:00	494:56:00
GOTERM_MF_FAT	GO:0031267~small GTPase binding	5	0.9861932938856016	710:40:00	190:18:00	861:51:00	29:53:00
GOTERM_BP_FAT	GO:0002790~peptide secretion	4	0.7889546351084813	910:19:00	1039:18:00	01:00:00	159:51:00
SMART	SM00119:HECTc	4	0.7889546351084813	853:53:00	603:26:00	848:48:00	958:36:00

INTERPRO	IPR005821:Ion transport	7	1.3806706114398422	384:12:00	533:06:00	01:00:00	124:48:00
GOTERM_BP_FAT	GO:0000226~microtubule cytoskeleton organiz	7	1.3806706114398422	159:51:00	84:31:00	01:00:00	584:26:00
GOTERM_MF_FAT	GO:0008134~transcription factor binding	13	2.564102564102564	1070:41:00	373:21:00	861:51:00	604:13:00
UP_SEQ_FEATURE	active site:Proton acceptor	26	5.128205128205128	513:24:00	393:38:00	01:00:00	894:07:00
GOTERM_BP_FAT	GO:0050905~neuromuscular process	5	0.9861932938856016	611:24:00	1052:32:00	01:00:00	697:53:00
GOTERM_MF_FAT	GO:0043130~ubiquitin binding	3	0.591715976331361	190:56:00	1058:43:00	861:51:00	62:25:00
GOTERM_BP_FAT	GO:0016079~synaptic vesicle exocytosis	3	0.591715976331361	1004:36:00	733:43:00	01:00:00	128:59:00
GOTERM_BP_FAT	GO:0042060~wound healing	7	1.3806706114398422	698:40:00	458:29:00	01:00:00	1070:18:00
UP_SEQ_FEATURE	domain:BAR	3	0.591715976331361	804:46:00	703:03:00	01:00:00	166:26:00
UP_TISSUE	Testis	103	20.315581854043394	973:18:00	1003:15:00	704:53:00	714:06:00
GOTERM_MF_FAT	GO:0019899~enzyme binding	11	2.1696252465483234	55:05:00	324:30:00	861:51:00	599:41:00
UP_TISSUE	Eyeball	13	2.564102564102564	1090:36:00	303:35:00	719:34:00	902:50:00
UP_SEQ_FEATURE	repeat:Kelch 3	5	0.9861932938856016	741:26:00	687:26:00	01:00:00	469:26:00
UP_SEQ_FEATURE	repeat:Kelch 1	5	0.9861932938856016	741:26:00	687:26:00	01:00:00	469:26:00
UP_SEQ_FEATURE	repeat:Kelch 2	5	0.9861932938856016	741:26:00	687:26:00	01:00:00	469:26:00
GOTERM_BP_FAT	GO:0035023~regulation of Rho protein signal t	6	1.183431952662722	620:39:00	127:46:00	01:00:00	478:06:00
INTERPRO	IPR017986:WD40 repeat, region	11	2.1696252465483234	993:56:00	541:50:00	01:00:00	1058:34:00
GOTERM_CC_FAT	GO:0016528~sarcoplasm	4	0.7889546351084813	934:22:00	796:41:00	861:51:00	621:22:00
SMART	SM00186:FBG	4	0.7889546351084813	644:06:00	496:09:00	858:28:00	869:01:00
GOTERM_CC_FAT	GO:0043235~receptor complex	6	1.183431952662722	798:36:00	206:55:00	861:51:00	707:30:00
INTERPRO	IPR000195:RabGAP/TBC	4	0.7889546351084813	1061:32:00	899:58:00	01:00:00	374:21:00
INTERPRO	IPR016130:Protein-tyrosine phosphatase, activ	6	1.183431952662722	612:49:00	1002:25:00	01:00:00	677:48:00
GOTERM_MF_FAT	GO:0051020~GTPase binding	5	0.9861932938856016	283:53:00	886:40:00	01:00:00	794:50:00
GOTERM_MF_FAT	GO:0050543~icosatetraenoic acid binding	2	).3944773175542406E	222:38:00	655:57:00	01:00:00	761:59:00
GOTERM_MF_FAT	GO:0050542~icosanoid binding	2	).3944773175542406E	222:38:00	655:57:00	01:00:00	761:59:00
GOTERM_MF_FAT	GO:0050544~arachidonic acid binding	2	).3944773175542406E	222:38:00	655:57:00	01:00:00	761:59:00
INTERPRO	IPR002319:Phenylalanyl-tRNA synthetase, clas	2	).3944773175542406E	947:43:00	442:50:00	01:00:00	724:54:00
INTERPRO	IPR002861:Reeler region	2	).3944773175542406E	947:43:00	442:50:00	01:00:00	724:54:00
GOTERM_BP_FAT	GO:0050882~voluntary musculoskeletal mover	2	).3944773175542406E	850:17:00	1005:52:00	01:00:00	949:20:00
UP_SEQ_FEATURE	domain:EF-hand 3	6	1.183431952662722	446:17:00	701:31:00	01:00:00	30:14:00
SMART	SM00192:LDLa	5	0.9861932938856016	428:58:00	546:05:00	860:08:00	951:24:00
UP_SEQ_FEATURE	domain:TSP type-1 1	4	0.7889546351084813	60:19:00	21:02:00	01:00:00	1068:11:00
UP_SEQ_FEATURE	domain:TSP type-1 2	4	0.7889546351084813	60:19:00	21:02:00	01:00:00	1068:11:00
GOTERM_CC_FAT	GO:0045177~apical part of cell	8	1.5779092702169626	503:04:00	728:51:00	861:51:00	485:28:00
GOTERM_MF_FAT	GO:0032182~small conjugating protein bindinc	3	0.591715976331361	397:32:00	652:16:00	01:00:00	261:14:00
UP_SEQ_FEATURE	compositionally biased region:Cys-rich	8	1.5779092702169626	199:49:00	906:16:00	01:00:00	404:07:00
INTERPRO	IPR000998:MAM	3	0.591715976331361	1016:22:00	872:03:00	01:00:00	187:03:00
INTERPRO	IPR017927:Ferredoxin reductase-type FAD-bin	3	0.591715976331361	1016:22:00	872:03:00	01:00:00	187:03:00
GOTERM_BP_FAT	GO:0016447~somatic recombination of immun	3	0.591715976331361	972:49:00	1012:49:00	01:00:00	135:29:00
GOTERM_BP_FAT	GO:0015844~monoamine transport	3	0.591715976331361	972:49:00	1012:49:00	01:00:00	135:29:00
UP_SEQ_FEATURE	domain:N-terminal Ras-GEF	3	0.591715976331361	159:29:00	550:37:00	01:00:00	705:26:00
INTERPRO	IPR001487:Bromodomain	4	0.7889546351084813	64:15:00	292:25:00	01:00:00	819:48:00
GOTERM_CC_FAT	GO:0000151~ubiquitin ligase complex	5	0.9861932938856016	735:55:00	870:34:00	861:51:00	45:22:00
GOTERM_BP_FAT	GO:0032313~regulation of Rab GTPase activit	4	0.7889546351084813	286:02:00	379:03:00	01:00:00	419:29:00

GOTERM_BP_FAT	GO:0032483~regulation of Rab protein signal t	4	0.7889546351084813	286:02:00	379:03:00	01:00:00	419:29:00
UP_SEQ_FEATURE	domain:Sushi 28	2	0.3944773175542406	711:21:00	126:16:00	01:00:00	350:02:00
UP_SEQ_FEATURE	region of interest:Interaction with C1D	2	0.3944773175542406	711:21:00	126:16:00	01:00:00	350:02:00
UP_SEQ_FEATURE	domain:Sushi 27	2	0.3944773175542406	711:21:00	126:16:00	01:00:00	350:02:00
UP_SEQ_FEATURE	domain:Sushi 25	2	0.3944773175542406	711:21:00	126:16:00	01:00:00	350:02:00
UP_SEQ_FEATURE	domain:Reelin	2	0.3944773175542406	711:21:00	126:16:00	01:00:00	350:02:00
UP_SEQ_FEATURE	domain:Sushi 26	2	0.3944773175542406	711:21:00	126:16:00	01:00:00	350:02:00
UP_SEQ_FEATURE	domain:Sushi 23	2	0.3944773175542406	711:21:00	126:16:00	01:00:00	350:02:00
UP_SEQ_FEATURE	domain:Sushi 24	2	0.3944773175542406	711:21:00	126:16:00	01:00:00	350:02:00
UP_SEQ_FEATURE	domain:Sushi 21	2	0.3944773175542406	711:21:00	126:16:00	01:00:00	350:02:00
UP_SEQ_FEATURE	domain:Sushi 22	2	0.3944773175542406	711:21:00	126:16:00	01:00:00	350:02:00
GOTERM_BP_FAT	GO:0007611~learning or memory	6	1.183431952662722	500:51:00	985:52:00	01:00:00	308:02:00
GOTERM_BP_FAT	GO:0050878~regulation of body fluid levels	6	1.183431952662722	500:51:00	985:52:00	01:00:00	308:02:00
GOTERM_CC_FAT	GO:0005626~insoluble fraction	21	4.142011834319527	219:33:00	96:49:00	861:51:00	606:08:00
SP_PIR_KEYWORDS	neurogenesis	8	1.5779092702169626	868:30:00	74:28:00	861:51:00	42:22:00
UP_SEQ_FEATURE	compositionally biased region:Poly-Pro	15	2.9585798816568047	108:49:00	276:16:00	01:00:00	154:26:00
GOTERM_CC_FAT	GO:0031252~cell leading edge	7	1.3806706114398422	71:11:00	719:26:00	861:51:00	878:25:00
UP_SEQ_FEATURE	calcium-binding region:2	7	1.3806706114398422	920:08:00	415:33:00	01:00:00	109:52:00
PIR_SUPERFAMILY	PIRSF037309:PP2A_PR55	2	0.3944773175542406	304:49:00	202:47:00	861:32:00	212:06:00
PIR_SUPERFAMILY	PIRSF037309:serine/threonine-protein phosph	2	0.3944773175542406	304:49:00	202:47:00	861:32:00	212:06:00
PIR_SUPERFAMILY	PIRSF002446:voltage-dependent calcium char	2	0.3944773175542406	304:49:00	202:47:00	861:32:00	212:06:00
SMART	SM00294:4.1m	3	0.591715976331361	1001:36:00	744:13:00	861:35:00	450:17:00
SMART	SM00612:Kelch	6	1.183431952662722	21:41:00	512:36:00	861:36:00	481:04:00
GOTERM_MF_FAT	GO:0005085~guanyl-nucleotide exchange fact	8	1.5779092702169626	1013:51:00	200:06:00	01:00:00	562:06:00
INTERPRO	IPR019781:WD40 repeat, subgroup	11	2.1696252465483234	394:52:00	960:11:00	01:00:00	171:11:00
SP_PIR_KEYWORDS	sh3 domain	10	1.9723865877712032	721:40:00	345:12:00	861:51:00	333:51:00
SMART	SM00239:C2	9	1.7751479289940828	371:28:00	316:06:00	861:38:00	1035:52:00
INTERPRO	IPR000798:Ezrin/radixin/moesin ERM	3	0.591715976331361	700:26:00	257:42:00	01:00:00	586:59:00
GOTERM_BP_FAT	GO:0019217~regulation of fatty acid metabolic	3	0.591715976331361	887:30:00	498:57:00	01:00:00	57:52:00
GOTERM_MF_FAT	GO:0005099~Ras GTPase activator activity	5	0.9861932938856016	765:58:00	207:04:00	01:00:00	827:43:00
GOTERM_MF_FAT	GO:0005543~phospholipid binding	7	1.3806706114398422	258:12:00	716:59:00	01:00:00	71:36:00
SP_PIR_KEYWORDS	repressor	16	3.155818540433925	899:15:00	193:26:00	861:51:00	698:24:00
INTERPRO	IPR000219:Dbl homology (DH) domain	5	0.9861932938856016	834:36:00	980:40:00	01:00:00	01:13:00
GOTERM_BP_FAT	GO:0016568~chromatin modification	11	2.1696252465483234	271:13:00	783:34:00	01:00:00	720:06:00
SP_PIR_KEYWORDS	lipid-binding	6	1.183431952662722	16:08:00	644:41:00	861:51:00	649:31:00
UP_TISSUE	Adult pancreatic islet	5	0.9861932938856016	838:58:00	36:40:00	853:54:00	310:56:00
UP_TISSUE	Parthenogenote	7	1.3806706114398422	114:31:00	375:14:00	853:55:00	844:58:00
INTERPRO	IPR001478:PDZ/DHR/GLGF	8	1.5779092702169626	16:19:00	219:28:00	01:00:00	515:20:00
SP_PIR_KEYWORDS	zinc-finger	40	7.889546351084813	352:18:00	373:51:00	861:51:00	247:47:00
GOTERM_BP_FAT	GO:0051960~regulation of nervous system dev	8	1.5779092702169626	915:09:00	587:26:00	01:00:00	352:14:00
GOTERM_BP_FAT	GO:0001558~regulation of cell growth	6	1.183431952662722	733:01:00	30:53:00	01:00:00	664:53:00
GOTERM_CC_FAT	GO:0030027~lamellipodium	5	0.9861932938856016	1088:41:00	719:20:00	861:51:00	497:44:00
KEGG_PATHWAY	mmu05200:Pathways in cancer	13	2.564102564102564	755:04:00	876:35:00	596:41:00	198:16:00
INTERPRO	IPR009091:Regulator of chromosome condens	3	0.591715976331361	692:41:00	910:44:00	01:00:00	1020:08:00

GOTERM_BP_FAT	GO:0046928~regulation of neurotransmitter se	3	0.591715976331361	88:38:00	709:45:00	01:00:00	232:54:00
GOTERM_BP_FAT	GO:0016445~somatic diversification of immunc	3	0.591715976331361	88:38:00	709:45:00	01:00:00	232:54:00
GOTERM_BP_FAT	GO:0016477~cell migration	11	2.1696252465483234	374:18:00	155:25:00	01:00:00	336:07:00
GOTERM_BP_FAT	GO:0006915~apoptosis	18	3.5502958579881656	971:59:00	151:12:00	01:00:00	1014:38:00
UP_TISSUE	Fetal intestine	2	0.3944773175542406	269:03:00	739:50:00	859:41:00	218:30:00
GOTERM_BP_FAT	GO:0016044~membrane organization	12	2.366863905325444	693:15:00	613:51:00	01:00:00	719:51:00
UP_TISSUE	Adipose tissue	6	1.183431952662722	549:45:00	371:01:00	859:57:00	323:56:00
INTERPRO	IPR001452:Src homology-3 domain	10	1.9723865877712032	344:36:00	260:34:00	01:00:00	547:02:00
GOTERM_MF_FAT	GO:0004721~phosphoprotein phosphatase act	8	1.5779092702169626	74:58:00	905:40:00	01:00:00	1072:54:00
COG_ONTOLOGY	Cell division and chromosome partitioning / Cyt	3	0.591715976331361	959:21:00	900:48:00	1046:54:00	592:39:00
GOTERM_MF_FAT	GO:0005097~Rab GTPase activator activity	4	0.7889546351084813	1044:38:00	638:57:00	01:00:00	960:52:00
KEGG_PATHWAY	mmu04320:Dorso-ventral axis formation	3	0.591715976331361	251:53:00	914:02:00	758:47:00	572:55:00
GOTERM_CC_FAT	GO:0005624~membrane fraction	20	3.9447731755424065	35:48:00	628:01:00	861:51:00	315:42:00
INTERPRO	IPR000504:RNA recognition motif, RNP-1	10	1.9723865877712032	311:40:00	250:08:00	01:00:00	622:59:00
GOTERM_MF_FAT	GO:0042805~actinin binding	2	0.3944773175542406	1046:38:00	1095:56:00	01:00:00	350:52:00
INTERPRO	IPR015447:Neurexin	2	0.3944773175542406	256:21:00	663:02:00	01:00:00	777:40:00
INTERPRO	IPR000633:Vinculin, conserved site	2	0.3944773175542406	256:21:00	663:02:00	01:00:00	777:40:00
INTERPRO	IPR019578:Serine-threonine phosphatase 2A,	2	0.3944773175542406	256:21:00	663:02:00	01:00:00	777:40:00
INTERPRO	IPR000009:Protein phosphatase 2A, regulatory	2	0.3944773175542406	256:21:00	663:02:00	01:00:00	777:40:00
INTERPRO	IPR018067:Protein phosphatase 2A, regulatory	2	0.3944773175542406	256:21:00	663:02:00	01:00:00	777:40:00
INTERPRO	IPR019573:Serine/threonine-protein phosphata	2	0.3944773175542406	256:21:00	663:02:00	01:00:00	777:40:00
INTERPRO	IPR000778:Cytochrome b245, heavy chain	2	0.3944773175542406	256:21:00	663:02:00	01:00:00	777:40:00
INTERPRO	IPR001033:Alpha-catenin	2	0.3944773175542406	256:21:00	663:02:00	01:00:00	777:40:00
INTERPRO	IPR019316:G8 domain	2	0.3944773175542406	256:21:00	663:02:00	01:00:00	777:40:00
GOTERM_BP_FAT	GO:0006432~phenylalanyl-tRNA aminoacylatic	2	0.3944773175542406	571:14:00	1085:19:00	01:00:00	737:47:00
GOTERM_BP_FAT	GO:0007019~microtubule depolymerization	2	0.3944773175542406	571:14:00	1085:19:00	01:00:00	737:47:00
GOTERM_BP_FAT	GO:0001956~positive regulation of neurotransı	2	0.3944773175542406	571:14:00	1085:19:00	01:00:00	737:47:00
GOTERM_BP_FAT	GO:0045600~positive regulation of fat cell diffe	2	0.3944773175542406	571:14:00	1085:19:00	01:00:00	737:47:00
GOTERM_BP_FAT	GO:0016080~synaptic vesicle targeting	2	0.3944773175542406	571:14:00	1085:19:00	01:00:00	737:47:00
SMART	SM00303:GPS	4	0.7889546351084813	897:08:00	695:27:00	861:51:00	54:16:00

Supplementary Table S11: Human transcripts that might have a mouse homolog

A2BP1
ADAMTS6
ANGPT1
ARHGAP17
ARHGAP6
ASTN1
ATP11B
ATP8A2
ATRNL1
AUTS2
BRCC3
BRE
CACNA2D1
CACNA2D3
CALN1
CDH12
CDH13
CDH8
CHM
CNTN4
CNTN5
CNTNAP2
COL22A1
COL4A5
CPEB4
CPNE4
CSMD1
CSPP1
CTNNA2
DACH1
DACH2
DCC
DLGAP1
DMD
DOCK4
DPP10
DYNC2H1
EMR4
EPHA3
EXOC6B
FAF1
FAM19A1
FARP1
FAT3
FBXL13/LRRC17
FHIT
FOXP1
FRMPD4
FRYL
GABRB3
GDPD4
GRIA1
GRIN2A
GRM5
GRM7

HIVEP1
IL1RAPL2
KCNIP4
KCNQ5
KLHL32
LDB2
LDLRAD3
LPP
LTBP1
LUZP2
MAGI2
MDGA2
MGA
MKLN1
MYO1D
NEGR1
NHS
NIN
NLGN1
NOX4
NRG3
NRXN1
NRXN3
ODZ1
ODZ2
PAPPA2
PARK2
PCDH17
PCDH9
PCLO
PGCP
PLD5
PLXDC2
POLA1
PRKG1
PTPRD
PTPRT
RABGAP1L
RELN
RGS7
RIMS2
ROBO2
RUNX2
SEC16B
SEMA3E
SLC25A21
SLC35F1
SLC39A10
SLC39A11
SMYD3
SNTG2
SORCS1
SPAG16
SPON1
STAG1
STK3
STXBP5L
SYT14

TBC1D23
TRPC4
TRPM3
USH2A
VPS13B
WDR70
XRCC4
ZMAT1

Supplemental table S12: Sequence identity analysis of human *vs* mouse KCNIP IV protein

KCNIP4 I (NP_079497) ↓
MNVRRVESISAQLEEASSTGGFLYAQNSTKRSIKERLMKLLPCSAAKTSSPAIQNSVEDELEM
ATVRHRP EALELLEAQSKFTKKELQILYRGFKNECPSGVVNEETFKEIYSQFFPQGDSTTYAH
FLFNAFDTDHNGAV SFEDFIKGLSILLRGTVQEKLNWAFNLYDINKDGYITKEEMLDIMKAIY
DMMGKCTYPVLKEDAPRQHVE TFFQKMDKNKDGVVTIDEFIESCQKDENIMRSMQLFENV I

mKCNIP4 A (AAH51130) ↓
MNVRRVESISAQLEEASSTGGFLYAQNNTKRSIKERLMKLLPCSAAKTSSPAIQNSVEDELEM
ATVRHRPEALELLEAQSKFTKKELQILYRGFKNECPSGVVNEETFKEIYSQFFPQGDSTTYAH
FLFNAFDTDHNGAVSFEDFIKGLSILLRGTVQEKLNWAFNLYDINKDGYITKEEMLDIMKAIY
DMMGKCTYPVLKEDAPRQHVETFFQKMDKNKDGVVTIDEFIESCQKDENIMRSMQLFENVI

KCNIP4 I and mKCNIP4 A 99% identities

KCNIP4 IV (NP_671712)
MNLEGLEMIAVLIVIVLFVKLLEQFGLIEAGLEDSVEDELEMATVRHRPEALELLEAQSKFTK
KELQILY RGFKNECPSGVVNEETFKEIYSQFFPQGDSTTYAHFLFNAFDTDHNGAVSFEDFIK
GLSILLRGTVQEKL NWAFNLYDINKDGYITKEMLDIMKAIYDMMGKCTYPVLKEDAPRQHVE
TFFQKMDKNKDGVVTIDEFIE SCQKDENIMRSMQLFENVI

mKCNIP4 C (NP_084541)
MNLEGLEMIAVLIVIVLFVKLLEQFGLIEAGLEDSVEDELEMATVRHRPEALELLEAQSKFTK
KELQILYRGFKNECPSGVVNEETFKEIYSQFFPQGDSTTYAHFLFNAFDTDHNGAVSFEDFIK
GLSILLRGTVQEKLNWAFNLYDINKDGYITKEEMLDIMKAIYDMMGKCTYPVLKEDAPRQHVE
TFFQKMDKNKDGVVTIDEFIESCQKDENIMRSMQLFENVI

KCNIP4 IV and mKCNIP4 C 100% identities
